# Aldehyde dehydrogenase 1a3 defines a subset of failing pancreatic β cells in diabetic mice

**DOI:** 10.1038/ncomms12631

**Published:** 2016-08-30

**Authors:** Ja Young Kim-Muller, Jason Fan, Young Jung R. Kim, Seung-Ah Lee, Emi Ishida, William S. Blaner, Domenico Accili

**Affiliations:** 1 Naomi Berrie Diabetes Center and Department of Medicine, Columbia University, New York, New York 10032, USA; 2 Department of Genetics and Integrated Program in Cellular, Molecular and Biomedical Studies, Columbia University, New York, New York 10032, USA

## Abstract

Insulin-producing β cells become dedifferentiated during diabetes progression. An impaired ability to select substrates for oxidative phosphorylation, or metabolic inflexibility, initiates progression from β-cell dysfunction to β-cell dedifferentiation. The identification of pathways involved in dedifferentiation may provide clues to its reversal. Here we isolate and functionally characterize failing β cells from various experimental models of diabetes and report a striking enrichment in the expression of aldehyde dehydrogenase 1 isoform A3 (ALDH^+^) as β cells become dedifferentiated. Flow-sorted ALDH^+^ islet cells demonstrate impaired glucose-induced insulin secretion, are depleted of Foxo1 and MafA, and include a Neurogenin3-positive subset. RNA sequencing analysis demonstrates that ALDH^+^ cells are characterized by: (i) impaired oxidative phosphorylation and mitochondrial complex I, IV and V; (ii) activated RICTOR; and (*iii*) progenitor cell markers. We propose that impaired mitochondrial function marks the progression from metabolic inflexibility to dedifferentiation in the natural history of β-cell failure.

## Abstract (web summary)

Diabetes is associated with the de-differentiation of β-cells into a more progenitor-like cell type. Here, the authors identify Aldh3 as a marker of de-differentiating β-cell in animal models of diabetes, and show Aldh3^+^ cells have impaired insulin secretion and mitochondrial dysfunction.

## Graphical abstract

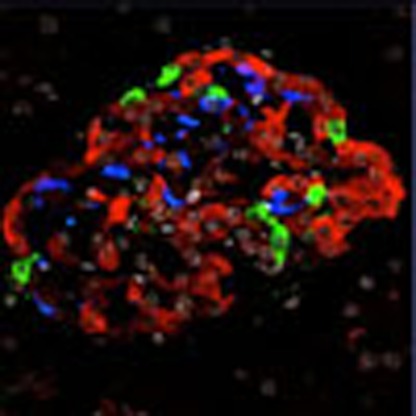

Diabetes arises as a consequence of combined abnormalities in insulin production and function^1^. Although alterations in either arm of this homeostatic loop can result in full-blown disease, in most individuals, the two abnormalities coexist. While target organs show an impaired response to insulin (so-called insulin resistance), β-cells of diabetics show a blunted and mistimed response to nutrients^2^. During the natural history of the disease, β-cell function markedly deteriorates^3^. In fact, an intrinsic susceptibility of the β cell to functional exhaustion-commonly referred to as ‘β-cell failure'-sets apart individuals who go on to develop diabetes from those that, at the same level of insulin resistance, do not^3^. Abnormalities of islet cell function in diabetes include an impaired insulin response to stimulus, a reduced number of β cells, and an inappropriate glucagon response^4^. This occurs despite the fact that reversal of hyperglycemia can partly restore β-cell function, even in patients with advanced disease^5^. Treatments range from preserving β-cell function by reducing the metabolic demand on β-cells, to increasing β-cell performance to meet the increased metabolic demand^2^. Notwithstanding this evidence, it is unclear whether the two primary components of β-cell failure, impaired insulin secretion and reduced β-cell mass, are mechanistically linked. We have shown that genetic ablation of Foxo function in β-cells impairs metabolic flexibility, that is, the ability to switch from glucose to lipids as a source of acetyl-CoA for mitochondrial oxidative phosphorylation, paving the way for β-cell dedifferentiation^6^,^7^,^8^,^9^. These two processes bookend β-cell failure, but we do not know what happens in between.

To address this question it's necessary to first discover biomarkers that can be used to isolate and characterize ‘failing' β cells. In this study, we report the discovery of an isoform of the enzyme aldehyde dehydrogenase 1 isoform A3 (ALDH1A3) as a biomarker of dysfunctional β cells. We isolated and characterized ALDH1A3-expressing islet cells, and compared their gene expression profiles in normal and diabetic mice. The data indicate that two reciprocal processes unfold in failing β cells: a decrease of mitochondrial function with presumptive activation of RICTOR, likely compensatory in nature, associated with progenitor cell-like features. We identify a narrow set of candidate genes that may affect the transition from a healthy to a dysfunctional β cell. The significance of this work consists in the discovery of a biomarker of β-cell dysfunction that can also be used to isolate failing cells; and in the identification of a pathogenic mechanism and a narrow set of potential effectors that can be tested for therapeutic relevance.

## Results

### Elevated ALDH1A3 is a common feature of diabetic β cells

**Figure 1:**
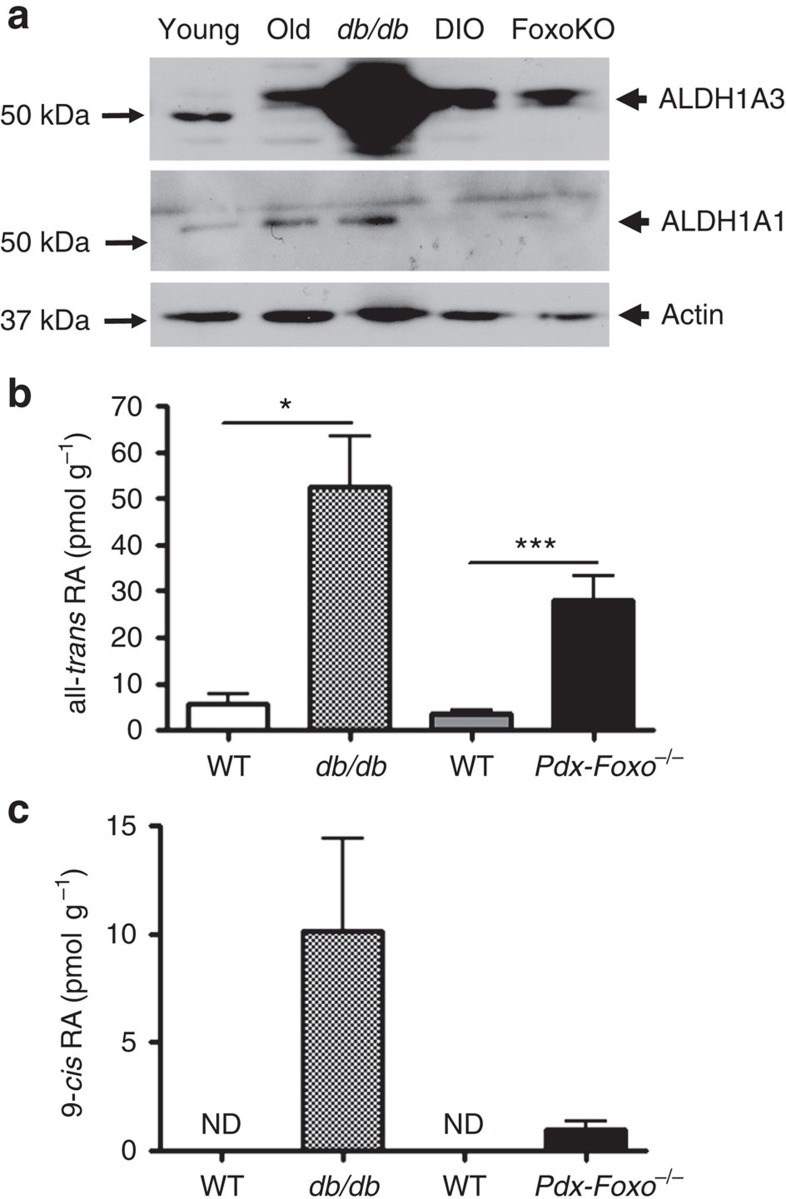
Increased levels and activity of ALDH1A3 in diabetic mice. (**a**) Western blot of ALDH1A3 in islets isolated from different models of wild-type and diabetic mice. The lower molecular weight band in young (3-month-old) mice is a non-specific band commonly observed with Aldh1a3 immunodetection. (**b**,**c**) All-*trans* (**b**) and 9-*cis* retinoic acid (**c**) in whole pancreas of control and diabetic mice. Shaded bars: *db/db* mice and their wild-type controls. Filled bars: Pdx-cre Foxo knockout mice and their wild-type controls (*n*=5 for each group). One asterisk indicates *P*<0.05 by one-way ANOVA. Error bars indicate s.e.m.

We reasoned that critical changes in gene expression during β-cell failure would be shared across multiple models of diabetes. We used two permutations of a genetic approach involving triple Foxo knockouts (Foxo1, 3a and 4) at two distinct developmental stages: (i) in pancreatic precursors (generated using *Pdx1-cre*-mediated gene knockout)^9^; (ii) in terminally differentiated β cells (generated using *Ins-cre*)^7^. The triple Foxo knockout faithfully replicates human MODY, a genetic form of diabetes caused by an intrinsic β-cell abnormality^10^. When we compared transcriptomes of islets from these models, a narrow selection of genes was uniformly affected across the board. Among them was ALDH1A3, expression of which increased three to sixfold with robust adjusted *P* values (Table 1). We tested the expression of ALDH1A3 in other models of diabetes including aging, diet-induced and *db/db* mutants, and found it to be increased too (Fig. 1a). We sought independent confirmation of this observation in the literature, and found that similar increases of ALDH1A3 had been observed in diabetic Nkx6.1 (ref. 11) and MafA knockout mice^12^, as well as in a cross of diabetes-sensitive versus resistant mice^13^. ALDH1A3 is notably absent from normal β cells^14^. In a recent study inspired by these findings, we found that ALDH1A3 is also elevated in islets from patients with type 2 diabetes^15^.

ALDH1A3 had two attractive features that justified further studies: ALDH1 activity marks human cancer progenitor cells^16^,^17^,^18^,^19^, and ALDH1A3 has been recognized as the isoform conveying increased ALDH1 activity in lung, ovary, breast, head and neck cancer and melanoma^20^,^21^,^22^. This observation is consistent with the notion that dedifferentiating β cells have progenitor-like features^11^,^23^,^24^. Moreover, ALDH-expressing cells can be readily isolated using live cell assays^25^. ALDH1A3 is one of 20 murine genes encoding NAD(P)^+^-dependent enzymes that catalyse aldehyde oxidation. ALDHs also have additional catalytic (for example, esterase and reductase) and non-catalytic activities. ALDH1A3 is also known as retinaldehyde dehydrogenase (RALDH3) owing to its ability to synthesize retinoic acid (RA) from retinal.

**Figure 2:**
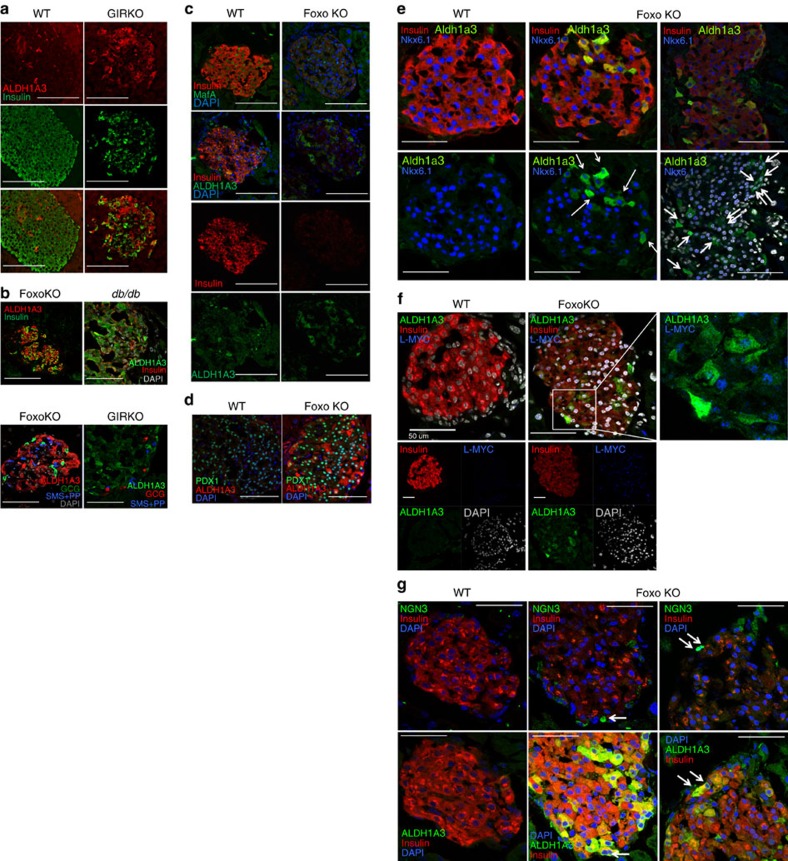
Localization of ALDH1A3 in mouse islets. (**a**) ALDH1A3 immunoreactivity in islets from normal and diabetic GIRKO mice. (**b**) Co-immunostaining of ALDH1A3 and insulin or glucagon, somatostatin (Sms), and Pp in *db/db, GIRKO,* and Pdx1-cre-driven Foxo knockout mice. (**c**–**e**) Co-immunostaining of ALDH1A3 with MafA (**c**), Pdx1 (**d**), or Nkx6.1 (**e**). ALDH^+^/Nkx6.1^−^ cells are indicated by the white arrows. MafA/ALDH1A3 (**c**) immunohistochemistry was performed on consecutive sections, whereas Pdx1/ALDH1A3 and NKX6.1/ALDH1A3 immunohistochemistry was performed on the same section. (**f**,**g**) Co-immunohistochemistry of ALDH1A3 with progenitor cell markers, L-myc (**f**) and neurogenin3 (**g**). ALDH1A3+/ Neurog3^+^ cells are indicated by the white arrows. Neurog3/ALDH1a3 immunohistochemistry was performed on consecutive sections. To better assess Neurog3/ALDH1A3-positive cells, we provide two representative sections from Foxo knockout mice. Scale bar, 100 μM in (**a**,**c**), scale bar, 50 μM in (**d**–**g**). In (**b**) left panel scale bar, 100 μM, right panel scale bar, 50 μM. DAPI, 4,6-diamidino-2-phenylindole.

The increase was specific to ALDH1A3, as other isoforms showed little if any change (Fig. 1a). Measurements of all-*trans*-RA and 9-*cis*-RA production in islets confirmed a correlation between ALDH1A3 levels and RA generation, indicating that the enzyme is catalytically active (Fig. 1b,c). We localized ALDH1A3 in islets using immunohistochemistry. ALDH1A3-positive cells were rare in normal islets (Fig. 2a). We studied a classic model of diabetes secondary to extreme obesity, *db/db* mice, as well as mice that develop diabetes as a consequence of extreme peripheral insulin resistance, brought about by targeted knockout of insulin receptor in muscle, fat and brain (GIRKO)^26^. Of note, the latter mice are lean and have no intrinsic β-cell abnormalities, but develop diabetes as a result of their inability to compensate for insulin resistance. In both models, the number of ALDH1A3-expressing cells rose considerably (Fig. 2a,b). There was heterogeneity of immunohistochemical signal intensity among ALDH1A3-expressing cells. We empirically defined them as ALDH1A3^low^ and ALDH1A3^hi^ cells. ALDH1A3 immunoreactivity showed a reciprocal pattern with insulin immunoreactivity such that ALDH1A3^hi^ cells were insulin-negative, while ALDH1A3^low^ cells retained some insulin immunoreactivity (Fig. 2a,b). We did not detect strongly insulin-immunoreactive cells that were also ALDH1A3-positive, nor did we detect any other endocrine cell type that co-localized with ALDH1A3 in mouse islets (Fig. 2b). These data show that ALDH1A3-positive cells are heterogeneous and are comprised of insulin-producing cells, as well as hormone-negative cells that can potentially represent a progenitor-like population.

We tested the expression of various β-cell markers in ALDH1A3-positive cells. They had weak MafA immunoreactivity (Fig. 2c), but retained Pdx1 immunoreactivity (Fig. 2d). Nkx6.1 was generally reduced in ALDH1A3-positive cells (Fig. 2e), with Nkx6.1 absent in a subset of ∼10% ALDH1A3-positive cells (Fig. 2e, right panels, white arrows). We also examined two progenitor cell markers, L-myc and Neurogenin3. Consistent with previous results, we found that L-myc expression increased in Foxo knockout islets and that ALDH1A3-positive cells were L-myc-positive (Fig. 2f). Moreover, there was a subset of ALDH1A3^+^/Neurog3^+^ cells (Fig. 2g, white arrows). In Foxo knockout islets, ALDH1A3^+^/Neurog3^+^ cells accounted for 5.2% of ALDH1A3^+^ cells (7/134, *n*=9 sections from 3 mice) while in wild-type islets, we found no Neurog3^+^ cells and hence no ALDH1A3^+^/ Neurog3^+^ cells. The staining method was validated in E12.5 fetal pancreas sections containing endocrine progenitors (Supplementary Fig. 1). These data provide immunohistochemical evidence that ALDH1A3 marks a heterogeneous cell population, with features of incipient β-cell failure (reduced insulin), and includes a subset of dedifferentiating (low MafA or Nkx6.1) or dedifferentiated cells (L-myc and Neurog3-expressing)^7^,^11^,^24^.

### ALDH1A3 overexpression does not impair insulin secretion

**Figure 3:**
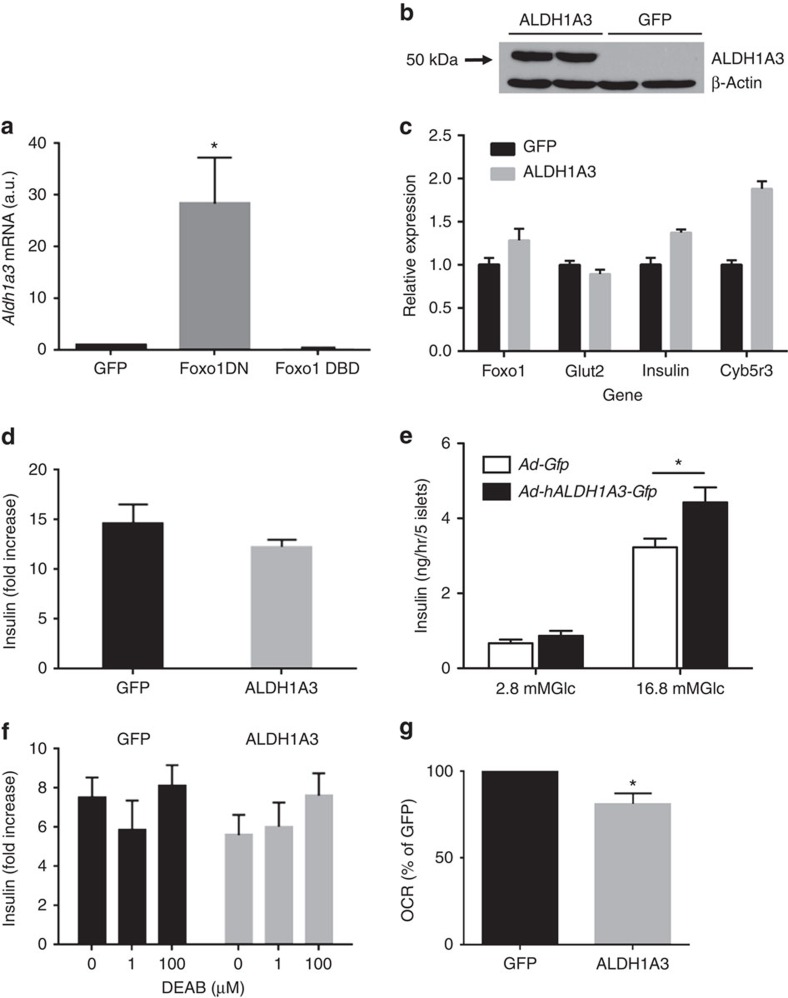
ALDH gain-of-function in β cells. (**a**) Effect of Foxo1 overexpression on *Aldh1a3* mRNA in Min6 cells. Foxo-DN is a truncated mutant that is unable to drive gene expression and competes with endogenous Foxo for DNA binding. Foxo- DNA-binding deficient (DBD) is a mutant unable to bind the Foxo response element, but can still function as a coregulator of gene expression^28^. (**b**) Western blot analysis of ALDH1A3 levels following lentiviral transduction in MIN6 cells. (**c**) Gene expression in Min6 cells stably expressing GFP or ALDH1a3. (**d**) Insulin secretion expressed as fold-increase from 5 to 20 mM glucose in MIN6 cells expressing either GFP or ALDH1A3 (*n*=8). (**e**) Insulin secretion in MIN6 cells stably transfected with ALDH1A3 (*n*=3). (**f**) Insulin secretion (expressed as in panel **d**) in islets isolated from *db/db* mice and their wild-type controls following treatment with the ALDH inhibitor N,N-diethylaminobenzaldehyde (DEAB) at the doses indicated (*n*=4). Each experiment was performed with pooled islets from 5 mice per genotype. (**g**) Area under the curve of oxygen consumption rates measured in Min6 cells stably expressing either GFP or ALDH1A3 (*n*=4 per group). One asterisk indicates *P*<0.05 by one-way ANOVA. Error bars indicate s.e.m.

As Foxo1 loss-of-function is associated with increased ALDH1A3 levels, we asked whether Foxo1 regulates ALDH1A3 in MIN6 insulinoma cells. We transfected wild-type and two different mutant Foxo1 constructs to investigate this point. The first mutant is a dominant-negative that binds to DNA but lacks the transactivation domain, preventing binding of RNA polymerase, hence transcription. When overexpressed, it outcompetes endogenous Foxo (1, 3a and 4) and effectively mimics the effect of a knockout^27^. The second mutant, DNA-binding deficient, does not bind to DNA, and fails to activate Foxo targets for which DNA binding is required^28^. Inhibition of Foxo1 by the dominant-negative mutant resulted in a ∼30-fold increase in *Aldh1a3* messenger RNA (mRNA), while the DNA-binding deficient mutant Foxo1 failed to activate *Aldh1a3* expression (Fig. 3a). This experiment shows that Foxo1 inhibits *Aldh1a3* independently of DNA binding, likely acting as a co-repressor^28^,^29^. These data are consistent with the possibility that activation of ALDH1A3 expression is an early correlate of reduced Foxo1 function.

Reduced RA signalling in islets has been linked to defective insulin secretion^30^. To test whether elevated ALDH1A3 activity affects β-cell function, we overexpressed ALDH1A3 in MIN6 cells using either transient transduction with adenovirus (Fig. 3b) or the derivation of stably transfected clones, and then measured expression of genes that are important for β-cell function or glucose-stimulated insulin secretion. In either case, we found no defects in gene expression (Fig. 3c) or insulin secretion (Fig. 3d). Moreover, we transduced islets of wild-type C57Bl/6J mice with ALDH1A3 adenovirus and found a small, but statistically significant increase of glucose-induced insulin secretion (Fig. 3e). ALDH1A3 activity can be inhibited by the irreversible inhibitor N,N-diethylaminobenzaldehyde^25^. We performed insulin secretion experiments in MIN6 cells overexpressing ALDH1A3, in the presence of N,N-diethylaminobenzaldehyde. But we didnot detect an effect of this compound to change insulin secretion (Fig. 3f). Finally, we measured oxygen consumption in MIN6 cells overexpressing ALDH1A3 as a surrogate of mitochondrial function, and found a modest decrease (Fig. 3g). However, in light of the fact that insulin secretion was normal (in MIN6) or slightly elevated (in primary islets), we suppose that this slight oxidative defect is unlikely to result in a functional change. These data showing that acute gain-of-function of ALDH1A3 doesnot compromise β-cell function suggest that ALDH1A3 is a marker, rather than a cause of β-cell dysfunction.

### Isolation and characterization of ALDH1A3-expressing islet cells

**Figure 4:**
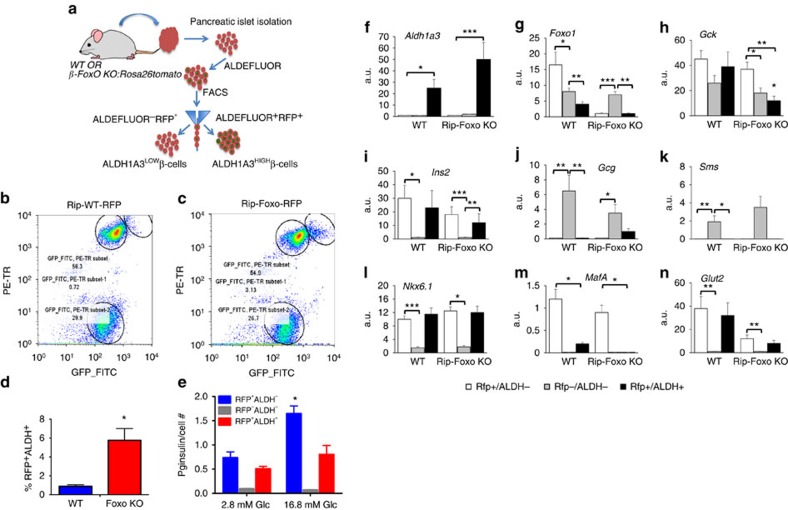
Isolation and characterization of ALDH^+^ cells. (**a**) Enrichment procedure to isolate ALDH-expressing islet cells. β cells are labelled red by Rip-cre-activated Tomato. Cells are incubated with aldefluor, and selected for tomato and aldefluor, yielding ALDH^−^ (low) and ALDH^+^ (high) cells. (**b**,**c**) Experimental validation. Islets from 6-month-old β-cell-specific (Rip-cre) Foxo knockouts and littermate controls were sorted as described. The different circles denote the three cell populations used in further studies: RFP^−^ALDH^−^, RFP^+^ALDH^−^ and RFP^+^ALDH^+^. (**d**) Quantification of RFP^+^ALDH^+^ cells in repeated sorts (*n*=5) of wild-type and Rip-Foxo knockout animals. (**e**), Insulin secretion in RFP^−^ALDH^−^ (GFP_FITC, PE TR subset 2), RFP^+^ALDH^−^ (GFP_FITC, PE TR subset), and RFP^+^ALDH^+^ (GFP_FITC, PE TR subset 1) cells isolated from wild-type mice (*n*=3). (**f**–**n**) Quantitative PCR analysis of selected transcripts in the different fractions isolated from islet cell preparations. One asterisk indicates *P*<0.05 by one-way ANOVA. Error bars indicate s.e.m.

We used a vital assay of ALDH activity to isolate ALDH1A3-expressing cells from mouse islets (Fig. 4a). The cell-permeable fluorescent ALDH substrate BODIPY-aminoacetaldehyde (aldeflour) is metabolized to the non-releasable derivative BODIPY-aminoacetate (BAA), thus permanently labelling ALDH-expressing cells. We used red fluorescent protein (RFP) to label β (or former β) cells by cre-mediated recombination^7^. Thereafter, we incubated cells with aldefluor, and selected for RFP (red) and aldefluor (green) fluorescence, yielding ALDH^−^ and ALDH^+^ cells. The latter should include dysfunctional/dedifferentiating β cells. In wild-type mice, we obtained three sub-populations: RFP^−^ALDH^−^ (non-β cells), RFP^+^ALDH^−^ (healthy β cells), and RFP^+^ALDH^+^ (dysfunctional β cells) (Fig. 4b). The latter represented <1% of total cells in normal islets. In separate experiments, we isolated RFP^+^ALDH^+^ cells from animals with β-cell-specific (Rip-cre) triple Foxo1 knockouts^8^. As predicted, the RFP^+^ALDH^+^ sub-population increased about sevenfold in this model (Fig. 4c,d).

We performed a preliminary characterization of ALDH^−^ and ALDH^+^ cells by measuring insulin secretion and gene expression. The predicted outcome of these experiments was that ALDH^+^ cells would be: (i) enriched in ALDH1A3; (ii) impaired in their ability to secrete insulin; (iii) depleted of markers of functional β cells, including Foxo1 (ref. 8). All predictions were borne out by the data. In glucose-stimulated insulin release experiments using ALDH^−^ versus ALDH^+^ cells, we found that only the former responded to glucose, providing critical evidence for a functional impairment of ALDH^+^ cells (Fig. 4e). *Aldh1a3* mRNA was restricted to the RFP^+^ALDH^+^ population in both wild-type and triple Foxo knockout mice (Fig. 4f). *Foxo1* was reduced by ∼70% in ALDH^+^ cells from wild-type mice (Fig. 4g). *Glucokinase* was nearly equally represented in all fractions, but was decreased in ALDH^+^ cells of triple Foxo knockouts (Fig. 4h), similar to previously reported single knockouts^7^. *Insulin2* and *Nkx6.1* expression were greatly enriched in the RFP^+^ population, while *glucagon* and *somatostatin* were enriched in the RFP^−^ population (Fig. 4i–l), providing another key element to support the identity of these cells. Foxo1 target *MafA* was enriched in the RFP^+^ALDH^−^ population and drastically reduced in RFP^+^ALDH^+^ cells. These data are consistent with the notion that ALDH^+^ cells are β cells that have lost key functional features (Fig. 4m). Finally, *Glut2* expression was restricted to RFP^+^ cells, regardless of their ALDH status, and was significantly decreased in Foxo knockouts, consistent with prior findings (Fig. 4n)^7^.

### Transcriptome of ALDH^+^ cells and progression of β-cell failure

**Figure 5:**
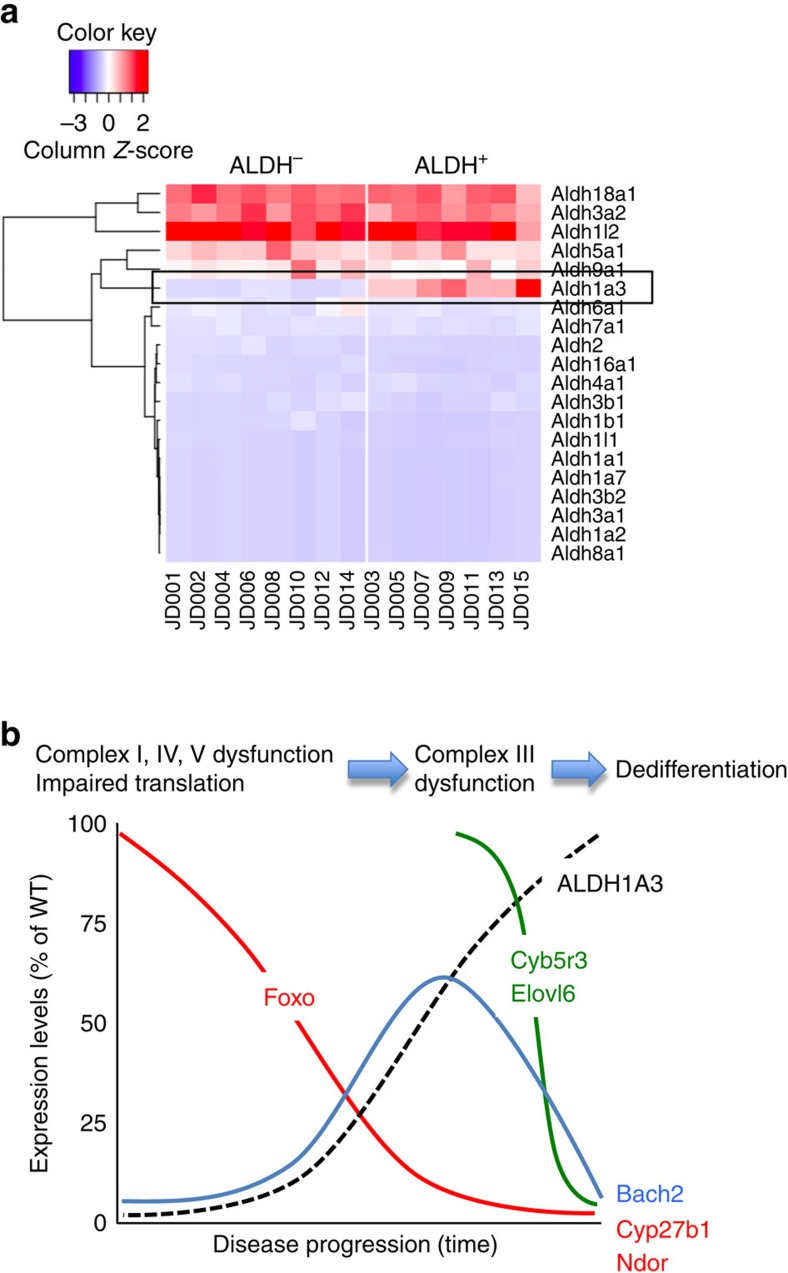
Comprehensive analysis of ALDH isoform expression in flow-sorted ALDH^+^ and ALDH^−^ cells. (**a**) Data from RNA sequencing of all ALDH transcripts are represented as column *Z*-scores, with red indicating high expression and blue indicating low expression. Each row represents a different ALDH isoform, and each column an individual sample used for analysis. RFP^+^ALDH^−^: JD001 through JD014, and RFP^+^ALDH^+^: JD003 through JD015. Aldh1a3 is boxed for reference. (**b**) Model of the relationship between changes in Foxo levels and gene expression signature of ALDH^+^ cells.

We carried out RNA sequencing analyses comparing ALDH^+^ with ALDH^−^ β cells (RFP^+^), as well as other islet cell types (RFP^−^) in wild-type mice. Moreover, we compared wild-type ALDH^+^ cells with triple Foxo-deficient ALDH^+^ cells generated by knocking out Foxo in mature β cells^8^. As a quality control, we interrogated expression of all 20 *Aldh* transcripts, and found that only *Aldh1a3* showed differential expression in the ALDH^+^ population (Fig. 5a). Moreover, in all comparisons between ALDH^+^ and ALDH^−^ cells, *Aldh1a3* was among the top differentially expressed genes (Supplementary Tables 1 and 2). This finding confirms the specificity and robustness of the enrichment technique.

First, we analysed differences in the levels of individual transcripts expressed in ALDH^+^ versus ALDH^−^ cells of wild-type mice. Using *P*<0.05 adjusted for multiple comparisons as threshold, we found 671 differentially expressed transcripts. A complete list is shown in Supplementary Table 1 and a curated sub-list in Supplementary Table 2. The transcripts fell into three broad categories: terminal differentiation of β cells, mitochondrial oxidative phosphorylation and ribosomal subunits. ALDH^+^ cells were depleted of transcripts encoding insulin, IAPP, Cpe, transthyretin, as well as other pancreatic hormones commonly found at low levels in β cells^31^, and were enriched in transcripts encoding markers of uncommitted endocrine progenitors, such as Pax6, Rfx6, Rfx7, Mlxipl, as well as transcription factors associated with progenitor cell differentiation, such as Ncor, Hic1 and Bach2. Next, there was a striking decrease of selected mitochondrial components: ∼30% of complex I NADH dehydrogenase subunits (13 of 41), complex IV cytochrome C oxidase subunits (8 of 25), and complex V F_1_ ATP synthase subunits (15 of 54) were substantially decreased. In addition, ∼30% of genes (28 of 92) encoding ribosomal 40S and 60S subunits were coordinately decreased (Supplementary Tables 1 and 2). Interestingly, 6 of the top 12 differentially expressed transcripts were long noncoding RNAs that have been associated with β-cell dysfunction: Malat1, Neat1, Meg3, Peg3, Sngh11 and Kcnq1ot1 (refs 32, 33). These highly abundant transcripts increased from 2.5- to 12-fold in ALDH^+^ cells (Supplementary Table 2).

We used the ‘upstream regulator analysis' function of the Ingenuity Analysis program to identify contributors to the phenotype of ALDH^+^ cells based on coordinated changes affecting their downstream effectors and regardless of whether the regulator's own expression levels changed. *Z*-scores were used to predict activation or inhibition of individual networks^8^. This analysis confirmed that the main differences between ALDH^+^ and ALDH^−^ cells could be subsumed under mitochondrial oxidative phosphorylation and revealed a strong potential activation of the RICTOR branch of mTOR signalling. Importantly, the same top five pathways were altered in ALDH^+^ cells isolated from wild-type and triple Foxo knockout mice, confirming that most differences between wild-type and Foxo-deficient ALDH^+^ cells are of a quantitative, rather than qualitative nature (Table 2).

Transcription factor network analyses indicated that ALDH^+^ cells have stem/progenitor cell properties, based on the combination of activated GATA, Wnt, Nanog and Neurog3 (ref. 34) and decreased Foxo and Notch signalling (Table 3 and Supplementary Table 3). Of note was also the marked inhibition of two master regulators of mitochondrial biogenesis and function, NFE2L2 and NRF1. NRF1 activates expression of EIF2A1 as well as genes required for mitochondrial biogenesis, function and mitochondrial DNA transcription^35^. The inhibition of NRF1 is consistent with the decrease of Tfam and Eif2 signalling in ALDH^+^ cells (Supplementary Table 3). NFE2L2 is involved in NRF2-mediated oxidative stress and unfolded protein response^36^.

This analysis also indicated activation of RICTOR (mTORC2) signalling. RICTOR promotes β-cell growth and insulin secretion^37^. However, other features of ALDH^+^ cells suggest that the activation of RICTOR is compensatory in nature. For example, ATF4-mediated signalling is inhibited, thus leading to decreased unfolded protein response and apoptotic signalling in response to endoplasmic reticulum stress. There are impairments in insulin and IGF1 receptor signalling, as well as inhibition of the transcriptional network overseen by nuclear receptor NR4A3, which is required for β-cell growth (Supplementary Table 3)^38^. The decrease in insulin/IGF receptor signalling is consistent with the homeostatic role of Foxo in these pathways, such that low Foxo would be expected to result in impaired insulin/IGF receptor signalling^39^. In addition, the mild activation of Src and EGF receptor signalling observed in ALDH^+^ cells suggests that cells are shifting from a fully differentiated phenotype maintained through insulin receptor/Foxo signalling, to a less differentiated phenotype dependent on oncogene signalling with features of progenitor cells (Supplementary Table 3).

Two other features of ALDH^+^ cells deserve mention: the decrease in estrogen receptor signalling, and activation of inflammation pathways, including NFKB1, MYD88, TICAM1, IFRD1, TLR7, CXCL12 and IL6 (Supplementary Table 3).

### Comparing wild-type and Foxo knockout ALDH^+^ cells

Next, we compared ALDH^+^ cells from wild-type and triple Foxo-deficient mice. The rationale was threefold: first, although ALDH1A3 expression is a marker of reduced Foxo activity, Foxo is *not* absent in the majority of these cells, and complete Foxo ablation may exacerbate their phenotype; second, it may reduce heterogeneity of ALDH^+^ cells; and third, because Foxo-deficient mice develop a MODY-like form of diabetes, this comparison might reveal qualitative differences between ALDH^+^ cells isolated from euglycemic versus diabetic animals. One can hypothesize that complete genetic ablation of Foxo mimics the final stages in the progression of the fate of ALDH^+^ cells and that, by analysing differences between wild-type and Foxo-deficient ALDH^+^ cells, it's possible to identify genes that mark the mechanistic progression to an advanced phase of cellular failure, or a tipping point towards dedifferentiation (Fig. 5b).

When we compared transcriptomes of wild-type versus triple Foxo knockout ALDH^+^ cells, we found few differentially expressed genes, as predicted (A partial list is in Table 4 and a complete list in Supplementary Table 4). The dearth of differences between wild-type and Foxo-deficient ALDH^+^ cells is wholly consistent with the concept that in diabetes there is a ‘spontaneous' loss of Foxo^6^,^7^,^8^, and that Foxo normally restrains ALDH1A3 expression (Fig. 3a). Nonetheless, these genes indicated potential pathogenic processes unfolding in failing β cells. A striking aspect of the gene expression profile of Foxo-deficient ALDH^+^ cells is the decrease in Cyb5r3. This gene encodes cytochrome b5 reductase isoform 3, one of four b5 reductase subunits (r1 through 4). Its expression is regulated by Foxo and Nrf, consistent with our findings^40^. Cyb5r3 has a membrane-bound and a soluble form, the latter of which is restricted to erythrocytes. It utilizes NADH and NADPH to synthesize long-chain FAs, and it's also required for mitochondrial complex III function. Cyb5r3-deficient cells show decreased NAD^+^/NADH ratios, mitochondrial respiration rate, ATP production and mitochondrial electron transport^40^. Notably, knockout of the related isoform Cyb5r4 causes early-onset β-cell failure in mice independent of peripheral insulin sensitivity^41^.

Other interesting genes that are specifically altered in Foxo-deficient ALDH^+^ cells include: *Elovl6*, *Ndor* and *Cyp27b1*. *Elovl6* is a long chain fatty acid elongase that has an important role in liver^42^. In β cells, its expression pattern mirrors Cyb5r3, and can potentially act in concert with the latter to synthesize long-chain FA. Similarly, the NAPDH-dependent oxidoreductase *Ndor*, whose expression levels track closely those of Foxo in ALDH^+^ cells, could be involved in mitochondrial processes related to Cyb5r3. Cyp27b1 is required for the synthesis of 1,25-OH vitamin D3, and evidence suggests that it participates in β-cell dysfunction in diabetes^43^. Finally, there were two transcripts that showed opposite changes in wild-type versus Foxo-deficient ALDH^+^ cells: the lncRNA Peg3, a parentally imprinted transcript whose methylation correlates with human islet function^44^, and Bach2, a transcription factor that has been implicated in type 1 diabetes susceptibility^45^,^46^, as well as β-cell stress^47^ (Table 4 and Supplementary Table 5).

## Discussion

The key finding of this work is the identification of a subpopulation of ALDH^+^ islet β cells. Based on their impaired insulin secretory properties and transcriptional signature, we propose that ALDH^+^ cells are failing β cells. They show conjoined features of the two cardinal processes bookending β-cell failure: mitochondrial dysfunction^8^ and progenitor-like features^7^. We propose the following model (Fig. 5b): when β cells are subject to increased demand for insulin production, they increase cellular metabolism and substrate flux through mitochondria. Foxo is activated to maintain normal oxidative function and prevent cellular overwork^6^,^7^. The tradeoff of increased Foxo function is increased Foxo degradation^6^, leading to eventual loss of the protein. As Foxo levels decline, ALDH1A3 is activated; thus, elevated levels of ALDH1A3 are a harbinger of β-cell failure. In the progression of the cellular pathology, mitochondrial complex I, IV and V functions are impaired, leading to reduced ATP production, stalling of protein translation and reactivation of genes that sustain a cellular progenitor program. When Foxo levels reach their nadir (a situation phenocopied by genetic knockout of Foxo), a further subset of genes becomes altered, including Cyb5r3, Elovl6 and Bach2 (Fig. 5b). We propose that these genes have a pathogenic role in β-cell dedifferentiation. Further studies to test their involvement in this process are underway, with the expectation that they are key mediators of progression of β-cell failure, and with the ultimate goal of developing therapeutic approaches to ameliorate β-cell dysfunction based on this model.

The role of ALDH1A3 in β-cell failure will have to be determined through further studies. In oncology, there is no consensus on whether ALDH1A3 is a marker or a pathogenic factor in cancer progression^18^. Our data indicate that ALDH1A3 overexpression does not untowardly affect β-cell function, but these experiments don't capture the complexity of the potential roles of ALDH1A3 in β-cell failure. For example, ALDH1A3 could promote mitochondrial dysfunction-the paramount feature of ALDH^+^ cells-by activating RAR/RXR signalling via RA production. This can result in increased Pparα function, a feature of metabolically inflexible β cells^8^. This effect may require a specific duration or additional contributors, and would have gone undetected in the experiments carried out so far. To address this and other possibilities, we are generating appropriate models of loss- and gain-of-function.

A prominent aspect of the gene expression profile of ALDH^+^ cells is the extent of impairment of mitochondrial gene expression. In addition to decreased complex I, IV and V subunit expression, ablation of Foxo also causes a profound decrease of Cyb5r3. The latter is likely a Foxo target^40^. Cyb5r3 mutations in humans cause methemoglobinemia^40^. The membrane-bound form of Cyb5r3 localizes to mitochondria and endoplasmic reticulum, where it catalyses desaturation and elongation of fatty acids, as well as cholesterol biosynthesis. Cyb5r3 generates reducing equivalents NAD^+^ and NADP^+^, and utilizes malonyl CoA and NADPH to make 18:1, 20:1 and 18:0 fatty acids in ratios of 65:20:15. Cyb5r3-deficient cells have decreased NAD^+^/NADH ratios, mitochondrial respiration, ATP production and mitochondrial electron transport. This results in higher oxidative stress and senescence^40^.

Cyb5r3 is a striking candidate as a β-cell failure gene. One can envision that in the context of already impaired mitochondrial complex I, IV and V function in ALDH^+^ cells, the drop in Cyb5r3 would result in the additional loss of NADH and NADPH reductase activity at the level of complex III, decreasing levels of reduced cytochrome B and C. This may lead to a drop in NAD+ levels to the point where glycolysis is effectively stalled for lack of reducing equivalents. Consistent with this idea, knockout of the related isoform, Cyb5r4, in mice causes early-onset β-cell failure independent of peripheral insulin sensitivity^41^. In yeast, the Cyb5r3 ortholog NQR1 extends lifespan in a Sirt1-dependent manner and increases oxidative metabolism^48^. Mitochondrial Cyb5r3 activity is increased by calorie restriction^48^ (a condition in which β cells oxidize more FA) and decreased by exposure to elevated glucose levels^49^.

In addition to its role in complex III function, Cyb5r3 could prevent β-cell failure through its role in fatty acid synthesis, effectively shunting away excess fatty acyl-CoA from mitochondria. This would be achieved in part through activation of the peroxisome proliferator-activated receptor (Ppar) program, an interesting feature consistent with the paradoxical increase of Pparγ function in metabolically inflexible β cells^8^. A decrease of Cyb5r3 levels can lead to fatty acid accumulation in the oxidative pathway, and worsen mitochondrial stress through formation of peroxides or other superoxide products^50^. In this regard, it should be noted that another gene specifically affected by Foxo knockout in ALDH^+^ cells is Elovl6, whose function to increase long-chain fatty acid synthesis complements that of Cyb5r3. While isolated knockout of Elovl6 has no apparent detrimental effect in β cells^51^, it remains to be seen whether this is also true in the context of the failing β cell and in the absence (or deficiency) of Cyb5r3.

An additional interesting candidate emerging from the analysis of Foxo-deficient ALDH^+^ cells is Bach2. It increases in wild-type ALDH^+^ cells, but decreases in Foxo knockout ALDH^+^ cells (Fig. 5b). Our interpretation of these data is that Bach2 is induced by Foxo when the latter undergoes nuclear translocation^6^,^7^, and decreases as Foxo is cleared from β cells. A transcriptional repressor first identified as a lineage selector of B-lymphocytes^52^, Bach2 has emerged as a genetic susceptibility locus in genome-wide association studies of human type 1 diabetes^45^,^46^, and has been found to protect β cells from apoptosis and oxidative stress^47^. In addition, its ability to regulate differentiation in the haematopoietic lineage^52^ raises the question of whether it has similar effects in endocrine cells, a hypothesis consistent with Bach2's ability to drive transcription from Maf sites, which are known to confer β-cell transcriptional features^12^,^53^.

ALDH^+^ cells are strikingly enriched in selected lncRNAs: 6 of the 12 top differentially expressed transcripts belong to this category. At least three of these transcripts have previously been linked to human β-cell dysfunction: Malat1, Meg3 and Kcnq1ot1. Malat1 is encoded in an enhancer cluster associated with β-cell-specific transcription factors^54^. Meg3 is part of an imprinted locus that confers susceptibility to type 1 diabetes^55^ and includes the atypical Notch ligand Dlk1, a negative regulator of adipocyte differentiation, as well as another gene, Rtl1, whose transcripts are also among the top enriched mRNAs in ALDH^+^ cells (Supplementary Table 1)^33^. Finally, Kcn1qot1 is part of an imprinted locus that includes IGF2 and the Beckwith-Wiedemann locus^32^ and has been linked to type 2 diabetes susceptibility^56^. We don't know the targets, let alone the functional consequences, of these changes in the lncRNA profile of ALDH^+^ cells, but we envision them to herald epigenetic changes leading to dedifferentiation.

In sum, the present work advances our understanding of β-cell failure and provides a series of testable targets to explain mechanisms of progression from impaired insulin secretion to cellular dysfunction and dedifferentiation.

## Methods

### Animal experiments

We performed genotyping as described^57^,^58^. Mice were maintained on a *mixed* 129J-C57BL/6 background. We derived control genotypes from the same litters. Owing to the complexity of genotyping the five mutant alleles (three Foxo alleles, Rosa26-Tomato and Rip-cre), we used different combinations of Foxo1, 3 and 4 floxed mice without Rip-cre transgene or Rip-cre mice (Jax Stock #003573) without Foxo floxed alleles as controls^59^. These mice were indistinguishable from *mixed* 129J-C57BL/6 mice in all metabolic tests. All mice were fed normal chow and maintained on a 12-hour light–dark cycle (lights on at 7:00AM). Sample size calculations were based on the variance observed in prior experiments^8^,^60^. The Columbia University Institutional Animal Care and Utilization Committee approved all experiments.

### Cell lines

We used the mouse insulinoma cell line MIN6, obtained from American Type Culture Collection, and previously characterized for its ability to secrete insulin^6^.

### RNA profiling

We performed RNA-sequencing using Illumina library preparation, Illumina 2000/2500 sequencing instrument, and standard bioinformatics. We determined differential expression by statistical testing based on negative binomial distribution using `DESeq' package of the R software. We visualized transcript reads with Integrative Genome Viewer (Broad Institute), and performed pathway analyses with Ingenuity Pathway Analysis (Ingenuity Systems).

### Fluorescence-activated cell sorting

We isolated islets by collagenase digestion from 6-month-old *Rip-cre Foxo1,3a,4*^*lox/lox*^;*RFP (ROSA-Tomato)* and *Rip-cre Foxo1,3a,4*^+/+^;*RFP* mice (10–20 animals per genotype). Briefly, after clamping the common bile duct at its entrance to the duodenum, we injected 3 ml of M199 medium containing 1 mg of collagenase P (Roche Molecular Biochemicals, Indianapolis, Ind.) per ml into the duct. We removed the pancreas and incubated it at 37 °C for 17 min. Thereafter, we added 30 ml of ice-cold M199 medium containing 10% newborn calf serum to stop the reaction. We dispersed the digested pancreata by pipetting and rinsed twice with 30 ml of the same medium. After filtering the tissue suspension through a Spectra-mesh (408 μm; Spectrum Laboratories, Inc.), we resuspended the digested tissue in 10 ml of Histopaque and overlaid it with 10 ml of M199 medium. We centrifuged the sample at 1,700*g* for 20 min, and collected the islets from the interface^61^. We dispersed the islets using tryspin digestion, washed twice with cold M199 medium, and incubated cells with the fluorescent ALDH substrate BODIPY-aminoacetaldehyde (aldeflour) for 1 h before flow cytometry. Thereafter, cells were applied to a BD Influx sorter and analysed with a BD LSRII instrument. We gated cells for RFP (red) and aldefluor (green) fluorescence, yielding RFP^−^ALDH^−^ (GFP_FITC, PE TR subset 2), RFP^+^ALDH^−^ (GFP_FITC, PE TR subset) and RFP^+^ALDH^+^ cells (GFP_FITC, PE TR subset 1). We obtained three sub-populations: RFP^−^ALDH^−^ (non-β cells), RFP^+^ALDH^−^ (β cells) and RFP^+^ALDH^+^ (ALDH-positive β cells).

### Immunoblot and immunohistochemistry

We performed immunoblotting and immunohistochemistry as previously described^7^. We used the following antibodies: rabbit primary antibodies to FoxO1 (Santa Cruz, 1:100, Cell Signaling), Somatostatin (DAKO, 1:2000), Aldh1a3 (Novus, 1:100), Neurog3 (Beta Cell Biology Consortium, 1:100) and MafA (Bethyl, 1:200); guinea pig primary antibodies to Insulin (DAKO, 1:2000) and Glucagon (DAKO, 1:1000) and Pdx1 (Millipore, 1:100); sheep primary antibody to Somatostatin (Novus, 1:200); goat primary antibodies to Pancreatic Polypeptide (Novus 1:500), Somatostatin (Chemicon, 1:250), Nkx6.1 (Santa Cruz, 1:100) and L-Myc (R&D, 1:100); and mouse primary antibodies to Aldh1a3 (LSBio, 1:100) and Glucagon (Sigma, 1:500; ref. 57).

### Mitochondrial function

We used the XF24-3 respirometer (Seahorse Bioscience) with 24-well plates. We used the F_1_F_0_ ATP synthase inhibitor oligomycin to assess uncoupling, carbonyl cyanide-p-trifluoromethoxyphenylhydrazone (FCCP) to estimate maximum respiration, and rotenone to measure non-mitochondrial respiration^62^.

### RNA measurements

We used standard techniques for mRNA isolation and quantitative PCR. PCR primer sequences have been published^7^.

### Statistical analyses and general methods

Sample sizes were estimated from expected effect size based on previous experiments. No randomization or blinding was used. We present data as means±s.e.m. We used two-tailed Student's *t*-test, one-way analysis of variance (ANOVA) or two-way ANOVA for data analysis, and the customary threshold of *P*<0.05 to declare statistically significant differences.

### Data availability

The RNA sequencing data that support the findings of this study have been deposited in the NCBI Gene Expression Omnibus as GSE78966.

## Additional information

**How to cite this article:** Kim-Muller, J.Y. *et al*. Aldehyde dehydrogenase 1a3 defines a subset of failing pancreatic β cells in diabetic mice. *Nat. Commun.* 7:12631 doi: 10.1038/ncomms12631 (2016).

## Supplementary Material

Supplementary Information Supplementary Figures 1-2, Supplementary Tables 1-5

## Figures and Tables

**Table 1:** Comparison of the top ten transcripts in two models of Foxo knockout β cells.

**β-cell-specific triple FoxO knockout (Rip-cre)**				**Pan-pancreatic triple FoxO knockout (Pdx-cre)**			
Gene symbol	RefSeq	*P*	Fold change	Gene symbol	RefSeq	*P*	Fold change
Serpina7	NM_177920	0.0057	6.10	Serpina7	NM_177920	0.005	11.23
Rsl1	NM_001013769	0.0645	4.99	Penk	NM_001013769	1.45 × 10^−5^	9.24
Tcea1	NM_011541	0.0804	4.72	Aldh1a3	NM_053080	5.18 × 10^−7^	5.59
Ly96	NM_016923	0.0210	3.54	Aass	NM_013930	0.0008	5.29
Asb11	NM_026853	0.0011	2.93	Rsl1	NM_001013769	0.0645	4.63
Tc2n	NM_001082976	0.0428	2.91	Fabp3	NM_010174	0.0070	3.49
Aldh1a3	NM_053080	5.18 × 10^−7^	2.87	Zfp423	NM_033327	5.70 × 10^−6^	3.40
Fabp3	NM_010174	0.0070	2.70	Ly96	NM_016923	0.0210	3.39
Bet1	NM_009748	0.0558	2.69	Asb11	NM_026853	0.0011	3.36
Naa38	NM_133939	0.04334	2.68	Tmed6	NM_025458	0.0097	3.29

List of the 10 most overexpressed genes from RNA sequencing analysis of β cells isolated from β-cell-specific and pan-pancreatic Foxo triple Foxo knockouts compared with their relevant wild-type controls.

**Table 2:** Pathway analysis of RNA sequencing in wild-type and Foxo knockout β cells.

	***P***
Wild-type ALDH^−^ versus ALDH^+^	
Oxidative phosphorylation	8.58E^−27^
Mitochondrial dysfunction	1.02E^−23^
EIF2 signalling	1.31E^−15^
mTOR signalling	1.81E^−10^
Regulation of eIF4 and p70S6K signalling	7.17E^−10^
	
Foxo knockout ALDH^−^ versus ALDH^+^	
EIF2 signalling	6.88E^−08^
Oxidative phosphorylation	1.29E^−07^
Mitochondrial dysfunction	2.14E^−06^
Regulation of eIF4 and p70S6K signalling	1.05E^−04^
mTOR signalling	3.78E^−04^

The table summarizes top pathways from transcriptome analysis of ALDH^−^ versus ALDH^+^ cells.

**Table 3:** Progenitor-like features of ALDH^+^ cells.

**Transcription factor**	***Z*****-score**	***P***
GATA4	2.607	1.00 × 10^−1^
GATA6	2.111	1.00 × 10^−1^
NKX6.1	1.969	2.68 × 10^−2^
PDX1	1.575	2.11 × 10^−6^
NANOG	1.508	1.62 × 10^−2^
GLIS3	1.384	8.57 × 10^−4^
CTNNB1	1.366	2.66 × 10^−2^
HNF1A	1.028	1.39 × 10^−2^
NEUROD1	0.741	4.70 × 10^−4^
NEUROG3	0.791	1.38 × 10^−4^
RBPJ	−2.130	1.00 × 10^−1^
FOXO1	−1.811	4.91 × 10^−3^
FOXO3	−1.400	2.92 × 10^−3^
FOXO4	−0.640	1.87 × 10^−2^
HNF4A	−1.212	1.39 × 10^−2^
NKX2.2	−1.000	2.65 × 10^−4^

*Z*-score analysis of transcriptional networks involved in pancreas development in ALDH^+^ cells.

**Table 4:** Top 25 differentially expressed transcripts in ALDH^+^ cells from wild-type and Foxo knockout mice.

**Gene**	**Wild-type**	**Foxo knockout**	**Fold Change**	**Log**_**2**_ **fold change**	***P***	**Adjusted** ***P***
Foxo1	1005.01	131.37	0.13	−2.94	2.23E-11	3.47E-07
Cyb5r3	5076.01	1742.73	0.34	−1.54	8.29E-09	6.46E-05
Cyp27b1	206.80	4.83	0.02	−5.42	5.10E-07	0.002649712
Elovl7	384.62	38.27	0.10	−3.33	1.46E-06	0.005684329
Hip1r	1463.56	467.44	0.32	−1.65	2.98E-06	0.009288892
Bach2	211.79	11.73	0.06	−4.17	2.45E-05	0.052959279
Ctsl	2272.77	5237.71	2.30	1.20	3.39E-05	0.052959279
Etl4	1573.45	590.80	0.38	−1.41	3.21E-05	0.052959279
Muc4	3932.14	763.76	0.19	−2.36	3.32E-05	0.052959279
Ptprt	753.26	181.42	0.24	−2.05	2.71E-05	0.052959279
Dnahc17	112.89	1.32	0.01	−6.41	3.87E-05	0.054903167
Spp1	3933.14	1896.47	0.48	−1.05	0.000104638	0.136037989
Gpc6	72.93	0.00	0.00	NA	0.000119737	0.14369393
Cxcl13	71.93	0.00	0.00	NA	0.000135081	0.150528447
Prnd	93.91	1.06	0.01	−6.47	0.000149671	0.155667934
2010015L04Rik	249.75	32.76	0.13	−2.93	0.000188906	0.173360099
Ncam1	2471.57	1148.98	0.46	−1.11	0.000183578	0.173360099
Jam2	437.57	97.38	0.22	−2.17	0.000207049	0.179454216
Galntl4	316.69	53.81	0.17	−2.56	0.000328609	0.269822705
D0H4S114	613.40	190.91	0.31	−1.68	0.000492415	0.351638321
Hcn1	76.92	0.60	0.01	−7.01	0.000495868	0.351638321
Nog	61.94	0.00	0.00	NA	0.000451442	0.351638321
Cox6b1	229.77	695.81	3.03	1.60	0.000553818	0.362501015
Krba1	424.58	108.79	0.26	−1.96	0.000565259	0.362501015

NA, not applicable.

This table lists a subset of genes differentially expressed between wild-type and triple Foxo-deficient ALDH^+^ cells, arranged by *P* value.
