# Supplementary material for: Aldehyde dehydrogenase 1a3 defines a subset of failing pancreatic β cells in diabetic mice
Source: Nat Commun. 2016 Aug 30;7:12631. doi: 10.1038/ncomms12631 (PMC5013715; doi:10.1038/ncomms12631)
Supplement: Supplementary Information — Supplementary Figures 1-2, Supplementary Tables 1-5 [file ncomms12631-s1.pdf]

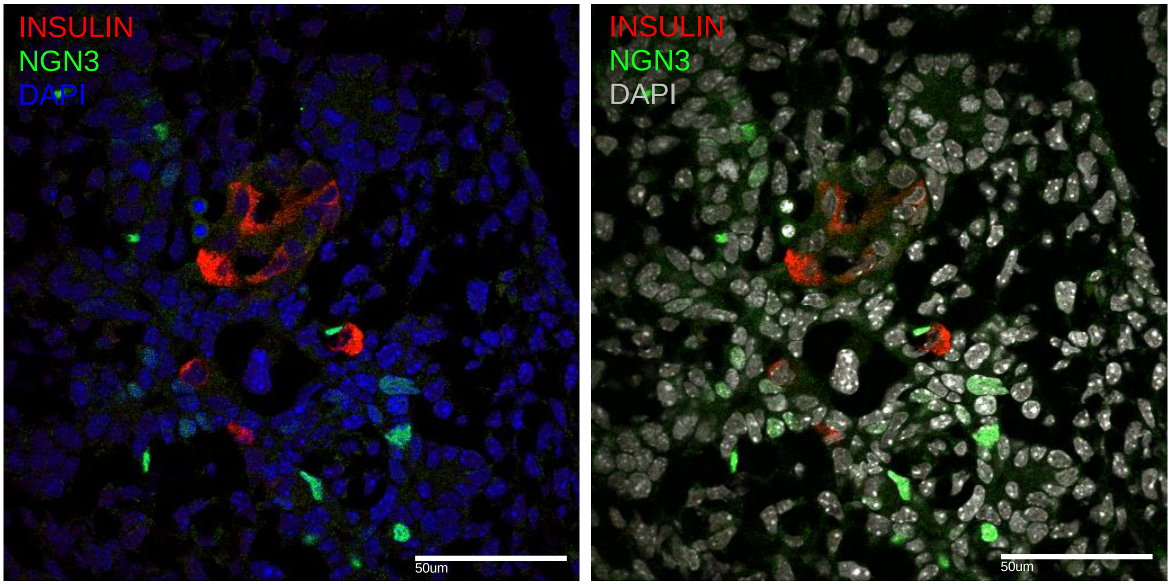

Supplementary Figure 1. Neurogenin3 localization in mouse E12.5 pancreas. Neurogenin3 immunohistochemistry in E12.5 mouse embryos.

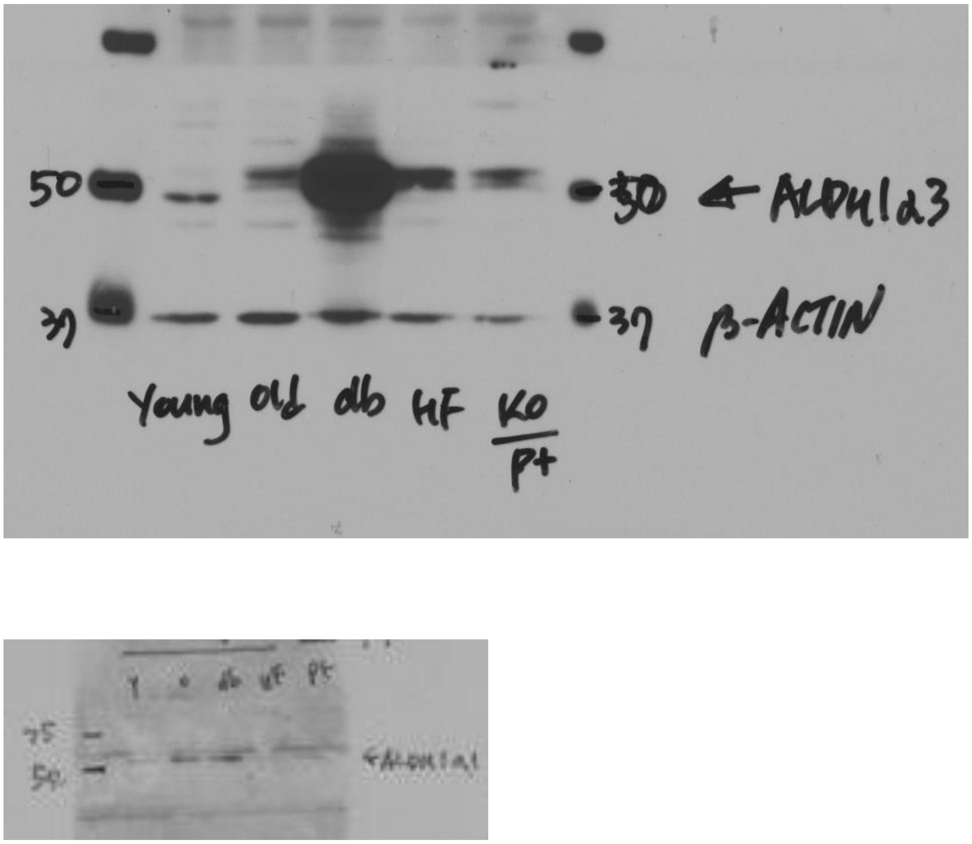

Supplementary Figure 2. Original Western Blots for Figure 1a.

Supplementary Table 1 Complete list of differentially expressed transcripts in wild-type ALDH⁻ vs. ALDH⁺ cells

Gene	baseMean all	baseMean ALDH–	baseMean ALDH+	Fold-Change	log2 Fold-Change	p-value	Adjusted p-value
Malat1	124697.0147	28383.6624	317323.7194	11.17980178	3.482822704	3.39E-60	5.14E-56
Meg3	5885.114667	1394.275924	14866.79215	10.66273318	3.414505386	3.32E-52	2.52E-48
Eml5	3444.730595	1336.696052	7660.799682	5.731145589	2.518823546	9.41E-46	4.76E-42
Snhg11	894.283641	142.4906105	2397.869702	16.82826464	4.072814506	4.52E-43	1.71E-39
Pyy	3861.622973	5362.393977	860.0809636	0.16039123	-2.640332839	3.61E-37	1.10E-33
Mlxipl	3287.320318	1521.052155	6819.856646	4.483644184	2.164671792	1.35E-33	3.42E-30
Neat1	3757.126356	1293.0687	8685.241668	6.716767383	2.747767066	1.34E-30	2.91E-27
Kcnq1ot1	2433.872718	775.6169559	5750.384243	7.413948598	2.890242113	1.90E-30	3.61E-27
Aldh1a3	368.1170314	43.89471581	1016.561662	23.15908974	4.533506645	6.71E-24	1.13E-20
Leng8	1779.943272	852.3747044	3635.080408	4.264650733	2.092427592	6.78E-22	9.35E-19
Peg3	16042.40826	10847.18501	26432.85474	2.436840039	1.285011551	6.41E-22	9.35E-19
Ppy	1932.691505	2650.807083	496.4603468	0.187286487	-2.416681283	1.23E-21	1.55E-18
Rtl1	345.7922558	47.55764639	942.2614745	19.81303841	4.308378235	4.43E-21	5.17E-18
Pclo	14100.27809	9753.78149	22793.2713	2.336865073	1.224574438	1.66E-19	1.80E-16
Srek1	1259.611237	591.4150864	2596.003537	4.389478044	2.134049398	6.07E-18	6.14E-15
Muc4	2414.456104	1406.187643	4430.993027	3.151068102	1.655840935	3.13E-17	2.97E-14
Ddx17	4121.159918	2713.272011	6936.93573	2.556668001	1.35426483	1.22E-15	1.09E-12
Kcnh6	747.3977997	300.980231	1640.232937	5.44963678	2.446160077	1.50E-15	1.27E-12
Ttc14	1143.975357	574.4419657	2283.042139	3.974365167	1.990724434	8.00E-15	6.39E-12
Mll3	4965.016558	3398.727474	8097.594726	2.382537225	1.252498755	9.11E-15	6.91E-12
Rps5	3173.536935	4075.843065	1368.924675	0.33586295	-1.574055439	1.36E-14	9.85E-12
Atp5e	1168.175777	1589.028404	326.4705228	0.205452918	-2.283120272	4.43E-14	3.05E-11
6720401G13Rik	491.3093791	172.3952163	1129.137705	6.549704391	2.711429795	1.89E-13	1.25E-10
Zfc3h1	1298.696003	594.3170947	2707.453819	4.555571163	2.187631946	2.63E-13	1.66E-10
A330076H08Rik	1758.528488	1051.033415	3173.518634	3.019426963	1.594274776	2.86E-13	1.73E-10
Rplp1	1578.464831	2091.323062	552.748368	0.264305586	-1.919721178	2.97E-13	1.73E-10
Cd63	2345.010105	3053.138983	928.7523494	0.304195896	-1.716927404	3.20E-13	1.80E-10
Clk1	1469.244663	846.7628045	2714.208381	3.205393962	1.680501684	6.57E-13	3.54E-10
Nktr	3576.811233	2171.434528	6387.564643	2.941633543	1.556617533	6.76E-13	3.54E-10
Pnn	1411.689954	816.7187615	2601.632339	3.185469052	1.671505821	1.48E-12	7.46E-10
Rpl32	2881.163807	3663.738743	1316.013935	0.359199721	-1.477141865	1.61E-12	7.87E-10
Atf4	7482.139646	9156.313331	4133.792275	0.451469071	-1.147300938	2.07E-12	9.80E-10
Rian	3597.354337	2481.437769	5829.187473	2.349116929	1.232118527	2.17E-12	1.00E-09
2810403A07Rik	1222.687467	688.0070889	2292.048222	3.331431114	1.736142062	3.57E-12	1.59E-09
Nfat5	2909.357259	1977.986671	4772.098435	2.412603939	1.270591098	9.11E-12	3.95E-09
Krtcap2	1391.465937	1836.717211	500.9633885	0.27274933	-1.874352443	1.01E-11	4.24E-09
Rgs9	823.2847727	416.4993312	1636.855656	3.930031895	1.974541021	1.97E-11	8.07E-09
Rps4x	1727.318491	2240.303365	701.3487439	0.31305972	-1.6754902	2.39E-11	9.56E-09

Rpl8	2402.659371	3049.552048	1108.874017	0.363618656	-1.459501874	4.04E-11	1.57E-08
Golgb1	8345.742428	6335.374515	12366.47825	1.951972724	0.964932894	4.45E-11	1.69E-08
Cst3	2615.619732	3293.566641	1259.725914	0.382480773	-1.386540869	1.02E-10	3.69E-08
Mt1	2089.802405	2664.698631	940.0099536	0.352764077	-1.50322444	9.99E-11	3.69E-08
Tbrg3	215.4640465	46.25900559	553.8741284	11.97332544	3.581751993	1.13E-10	3.99E-08
Cacna1a	843.2644231	448.7203277	1632.352614	3.637795111	1.863064289	1.36E-10	4.70E-08
Fnbp4	863.6837877	466.9660098	1657.119343	3.548693714	1.827288061	1.83E-10	6.18E-08
Rps3	2813.934375	3520.678579	1400.445967	0.397777285	-1.329967201	2.01E-10	6.61E-08
Kif12	1212.653456	725.8668125	2186.226742	3.011884143	1.590666275	2.12E-10	6.85E-08
Prrc2c	8966.535509	6914.764004	13070.07852	1.890169861	0.918515889	2.31E-10	7.15E-08
Ttr	4923.070751	6008.364008	2752.484236	0.458108768	-1.126237918	2.31E-10	7.15E-08
Egr1	7123.470171	5439.724564	10490.96139	1.928583196	0.947541383	3.31E-10	1.00E-07
Sfrs18	1114.682974	660.5287198	2022.991481	3.062685119	1.614797048	4.38E-10	1.30E-07
9530091C08Rik	193.7560951	40.71532863	499.837628	12.2763992	3.61781556	6.24E-10	1.82E-07
Dtx3	389.911961	151.4501784	866.8355261	5.723568868	2.516915004	7.06E-10	1.98E-07
Zbed6	2100.148856	1427.809836	3444.826896	2.412665054	1.270627643	6.94E-10	1.98E-07
Gm16907	275.9072982	84.01314317	659.6956082	7.852290526	2.973113552	7.40E-10	2.04E-07
Lamp1	7939.319946	9490.84653	4836.266779	0.509571698	-0.972642944	1.14E-09	3.09E-07
Itga4	720.4112402	379.2681164	1402.697488	3.698432394	1.886913904	1.17E-09	3.12E-07
Iapp	499863.3592	660135.5386	179319.0006	0.271639671	-1.880233904	1.49E-09	3.89E-07
Cox4i1	1299.685585	1686.100438	526.8558782	0.312470044	-1.678210207	1.82E-09	4.67E-07
Tet2	1379.552138	883.902481	2370.851452	2.682254551	1.423446158	2.92E-09	7.38E-07
Srp14	980.4443428	1323.754779	293.8234705	0.22196216	-2.171614345	3.61E-09	8.99E-07
Mir682	1380.799413	1777.938529	586.5211807	0.329888335	-1.599950332	3.67E-09	8.99E-07
Uqcrq	1081.424038	1415.559019	413.1540754	0.291866372	-1.776620097	4.12E-09	9.92E-07
Rps15	1831.08685	2313.775392	865.7097657	0.374154626	-1.418293484	4.51E-09	1.07E-06
Fosb	396.9418115	165.9351156	858.9552032	5.176452253	2.371963667	4.61E-09	1.08E-06
Cyp27b1	80.17526727	3.746697067	233.0324077	62.19675718	5.958767458	5.50E-09	1.27E-06
Zfp612	1138.63936	698.1519397	2019.6142	2.892800385	1.532466775	5.61E-09	1.27E-06
Rpl41	7227.255257	9232.17124	3217.42329	0.348501258	-1.520764231	6.15E-09	1.37E-06
Ddx26b	655.9202741	348.3886521	1270.983518	3.648177145	1.867175784	7.18E-09	1.58E-06
Hip1r	906.0685398	534.4832995	1649.23902	3.085669883	1.625583725	7.73E-09	1.68E-06
4930480K15Rik	65.07774389	1.364099629	192.5050324	141.1224139	7.140803333	9.01E-09	1.93E-06
Rps24	1568.410415	1994.060928	717.1093898	0.359622607	-1.475444378	9.24E-09	1.95E-06
Nme2	992.0190731	1300.026619	376.0039815	0.28922791	-1.78972132	1.07E-08	2.22E-06
D330022K07Rik	205.841139	55.46561314	506.5921906	9.133446147	3.191159307	1.17E-08	2.40E-06
Rbm26	1665.026644	1130.303931	2734.472069	2.419236095	1.27455157	1.19E-08	2.40E-06
Ndufa11	721.1160176	966.8464632	229.6551264	0.237530089	-2.073817818	1.31E-08	2.62E-06
Rps9	1562.017646	1981.657374	722.7381919	0.364714002	-1.455162505	1.44E-08	2.84E-06
Setd2	1870.046503	1298.239427	3013.660654	2.321344269	1.214960499	1.46E-08	2.84E-06
9430032N09Rik	244.0057524	77.25107994	577.5150973	7.475819079	2.902231655	1.50E-08	2.88E-06
Mat2a	1534.736793	1031.121671	2541.967036	2.465244508	1.301730743	1.53E-08	2.90E-06
6820431F20Rik	759.6451309	435.3045514	1408.32629	3.235266632	1.693884616	2.12E-08	3.97E-06

Zc3h7a	1224.332633	793.4819166	2086.034065	2.628962326	1.394493468	2.31E-08	4.28E-06
Park7	933.5962577	1222.52424	355.7402938	0.290988336	-1.780966771	2.83E-08	5.18E-06
Calr	5932.802875	7057.460259	3683.488106	0.521928282	-0.938076516	2.89E-08	5.22E-06
Zfp182	282.0122967	102.7396046	640.557681	6.234768798	2.640336062	3.03E-08	5.41E-06
Eid3	69.00371785	2.750018869	201.5111158	73.27626661	6.195274096	3.57E-08	6.23E-06
Gpr98	423.9359111	196.2944212	879.2188908	4.479082418	2.163203213	3.54E-08	6.23E-06
Ankrd12	1720.417204	1194.251845	2772.747923	2.321744727	1.215209358	3.61E-08	6.23E-06
Mbd5	1072.882873	680.0090795	1858.630459	2.733243592	1.450614041	4.12E-08	6.95E-06
Rpl14	1123.021808	1448.685903	471.6936175	0.325601027	-1.618822846	4.09E-08	6.95E-06
Mt2	3313.467797	4244.649102	1451.105186	0.341866937	-1.548493194	4.70E-08	7.84E-06
Psmb4	1784.130152	2229.83122	892.7280159	0.400356766	-1.320641907	5.25E-08	8.66E-06
Nav2	3628.636742	2764.208186	5357.493856	1.938165831	0.954692014	5.54E-08	8.94E-06
Pcsk4	206.2760136	61.18384699	496.4603468	8.114238826	3.020455766	5.52E-08	8.94E-06
Gm15421	1107.682334	1426.239572	470.567857	0.329936054	-1.599741655	6.31E-08	9.88E-06
Ogt	1260.427535	834.6780245	2111.926555	2.530229013	1.339267971	6.32E-08	9.88E-06
Rbm5	1920.132119	1362.673128	3035.050102	2.227276696	1.155280796	6.30E-08	9.88E-06
Bod1l	1671.043882	1163.533637	2686.064371	2.308540367	1.20698096	6.91E-08	1.07E-05
Ccdc56	634.6392382	850.6404192	202.6368762	0.238216844	-2.069652666	8.11E-08	1.24E-05
Snap25	6990.999772	5583.813014	9805.373289	1.756035395	0.812321924	8.69E-08	1.32E-05
Gabarap	2509.461602	3069.598222	1389.188363	0.452563581	-1.143807604	9.35E-08	1.40E-05
Pam	9800.780588	11442.09446	6518.152852	0.569664311	-0.81181607	1.19E-07	1.77E-05
Ppig	1518.226593	1051.386789	2451.906202	2.332068681	1.221610278	1.29E-07	1.90E-05
Cd44	1020.106568	655.4440025	1749.431698	2.66907881	1.416341905	1.43E-07	2.09E-05
Cox7a2	894.9022553	1190.375726	303.9553144	0.255344013	-1.969485864	1.45E-07	2.10E-05
Syp	4221.895934	3293.290757	6079.106287	1.84590634	0.884329353	1.79E-07	2.57E-05
Atxn7	851.8757747	528.6201002	1498.387124	2.834525443	1.503107219	2.17E-07	3.08E-05
Ccnl2	708.9787581	420.0960551	1286.744164	3.062976071	1.614934096	2.23E-07	3.13E-05
Psma6	1432.573254	1798.748389	700.2229834	0.389283453	-1.361107074	2.55E-07	3.54E-05
Dnahc17	42.40364262	0	127.2109279	Inf	Inf	2.76E-07	3.78E-05
Mdh1	2929.812363	3538.014862	1713.407365	0.484284954	-1.046071914	2.75E-07	3.78E-05
Fis1	377.4084833	522.7709486	86.68355261	0.16581555	-2.592348783	3.29E-07	4.45E-05
Prdx1	1903.520509	2344.748411	1021.064704	0.435468769	-1.19935884	3.72E-07	4.97E-05
Prpf39	518.560888	281.3809852	992.9206936	3.528741265	1.819153653	3.73E-07	4.97E-05
Col6a6	944.1553283	609.6256489	1613.214687	2.646238212	1.403942937	4.71E-07	6.22E-05
Gm17066	289.1343202	121.8658429	623.6712746	5.117687284	2.355491994	4.81E-07	6.29E-05
Swi5	890.0231708	1148.721406	372.6267002	0.324383874	-1.624225992	5.44E-07	7.05E-05
Cox17	561.6042762	750.0940595	184.6247095	0.246135411	-2.022475866	6.45E-07	8.30E-05
Clk4	823.3771513	521.3336184	1427.464217	2.738101221	1.45317578	7.31E-07	9.32E-05
Fth1	4264.9379	5041.991301	2710.8311	0.537650888	-0.895258401	8.28E-07	0.000104333
Rps27l	715.8016824	936.3597519	274.6855433	0.29335471	-1.76928194	8.32E-07	0.000104333
Rpl13a	1291.434964	1619.125126	636.0546393	0.392838471	-1.347991873	9.07E-07	0.000111832
Tmem181b-ps	285.4795395	122.5753542	611.28791	4.987037679	2.318183103	9.03E-07	0.000111832
Shfm1	284.5550301	400.3771753	52.91073991	0.132152238	-2.919727234	9.48E-07	0.000115947

Gm7694	38.65110787	0	115.9533236	Inf	Inf	1.05E-06	0.000127759
Setd5	4366.232198	3479.962503	6138.771589	1.764033832	0.81887823	1.07E-06	0.000128501
Dst	10942.1612	9094.673288	14637.13703	1.609418674	0.686539678	1.21E-06	0.000144648
Slc7a15	97.32465051	15.96164685	260.0506578	16.29221973	4.026111272	1.32E-06	0.000156516
Srrm2	15682.22232	11549.18273	23948.30149	2.073592742	1.052132574	1.36E-06	0.000159857
Ncoa6	3024.722863	2351.420432	4371.327725	1.859015795	0.894539028	1.40E-06	0.000162812
Ncor1	4756.034573	3821.501813	6625.100092	1.733637825	0.793802536	1.41E-06	0.000162812
Rev3l	2126.893097	1598.514407	3183.650478	1.991630769	0.993950209	1.43E-06	0.00016437
Rfx6	1900.222731	1409.360754	2881.946684	2.044860889	1.032002701	1.46E-06	0.000166394
A930012L18Rik	42.3315224	0.454699876	126.0851674	277.2931642	8.115268241	1.50E-06	0.000168352
Dhx57	473.5894326	260.6428597	899.4825784	3.451015614	1.787021001	1.51E-06	0.000168352
Gria3	306.6181575	134.0195937	651.8152852	4.863582014	2.282019246	1.49E-06	0.000168352
Bnip2	299.5960726	135.3069507	628.1743163	4.642587191	2.214929005	1.59E-06	0.000176512
Gcfc1	818.5148381	526.9863934	1401.571727	2.659597562	1.41120796	1.61E-06	0.000176894
Insrr	2924.26832	2273.913045	4224.978869	1.858021299	0.89376704	1.65E-06	0.000180243
Phc3	1515.320881	1094.310158	2357.342327	2.154181161	1.107139582	1.79E-06	0.000193945
Pkd1	1064.446332	726.4566899	1740.425615	2.395773401	1.260491461	1.96E-06	0.000210658
Ndufb9	951.5812564	1211.788764	431.1662422	0.355809738	-1.4908221	1.97E-06	0.000210659
Anxa5	3243.975719	3855.030718	2021.865721	0.524474607	-0.93105517	2.06E-06	0.000218601
D4Wsu53e	1461.268119	837.6123884	2708.579579	3.233690925	1.693181793	2.11E-06	0.000222815
Qdpr	917.8697902	1170.790528	412.028315	0.351923171	-1.506667589	2.13E-06	0.000222852
Rps20	880.677503	1126.822581	388.3873461	0.344674798	-1.536692278	2.17E-06	0.000225538
Mysm1	1421.579475	1019.555034	2225.628357	2.182940875	1.126273056	2.22E-06	0.000229527
B430010I23Rik	36.39958703	0	109.1987611	Inf	Inf	2.35E-06	0.000240834
5330434G04Rik	340.2046871	166.9501015	686.7138584	4.113288056	2.040292108	2.53E-06	0.000257955
Ndor1	400.7365395	209.903062	782.4034944	3.727451552	1.898189601	2.66E-06	0.000269397
Tia1	655.5193016	405.7638551	1155.030195	2.846557622	1.509218304	2.81E-06	0.000281964
Mycbp2	3956.556309	3169.403983	5530.860961	1.745079198	0.803292513	2.97E-06	0.000296631
Ssr2	1744.583094	2130.546138	972.6570059	0.456529426	-1.131220241	3.09E-06	0.000306238
Gdap10	159.7800565	49.41657315	380.5070232	7.699988058	2.944856208	3.24E-06	0.000318732
Gm10538	46.34416298	1.407738851	136.2170112	96.7629835	6.596383348	3.31E-06	0.000323552
Ubn2	2792.643118	2050.019873	4277.889609	2.08675519	1.061261359	3.49E-06	0.000339281
Gnb2l1	2891.811573	3663.386865	1348.660987	0.368145936	-1.441650318	3.65E-06	0.000352859
Cspp1	725.273222	464.2385584	1247.342549	2.686856847	1.425919458	4.32E-06	0.000415166
Rps11	1423.455085	1754.675605	761.0140463	0.433706404	-1.20520935	4.37E-06	0.000416959
Eef1a1	12058.09772	14428.42521	7317.442753	0.507154637	-0.979502386	4.83E-06	0.000457169
Ftl1	5236.563516	6257.954113	3193.782322	0.510355663	-0.970425094	4.87E-06	0.000457169
Gpr137b-ps	590.5667842	360.1200585	1051.460236	2.919749153	1.545844427	4.94E-06	0.000457169
Slc2a4rg-ps	127.0909647	33.59286789	314.0871582	9.349816728	3.224938086	4.91E-06	0.000457169
Zfp187	1394.277116	1012.937188	2156.956971	2.129408414	1.090452681	4.92E-06	0.000457169
Ubl5	669.8284767	868.5257038	272.4340225	0.313674105	-1.672661663	5.49E-06	0.000504996
Bach2	90.34029857	16.17984297	238.6612098	14.75052695	3.88269459	5.61E-06	0.000512996
Ndufc2	1196.019296	1486.696348	614.6651912	0.413443668	-1.274237321	5.86E-06	0.000532564

Tmem59	2694.538932	3203.679762	1676.257271	0.523228723	-0.934486354	6.40E-06	0.0005778
Uqcrh	902.7605125	1142.497809	423.2859192	0.370491668	-1.432486996	6.81E-06	0.000611076
Gm5148	1134.385222	1412.820284	577.5150973	0.408767558	-1.290647394	7.31E-06	0.000652729
Kcnmb1	213.780519	86.51261043	468.3163362	5.413272515	2.436501018	7.58E-06	0.000672217
Fermt1	33.02230576	0	99.06691727	Inf	Inf	7.84E-06	0.000691542
Tnrc6a	1225.919763	880.8575235	1916.044241	2.175203356	1.121150282	8.23E-06	0.000722211
Cttnbp2	1449.986654	1069.483246	2210.993472	2.06734746	1.047780884	8.50E-06	0.000736801
Gcc2	1836.373666	1394.079027	2720.962944	1.951799641	0.964804963	8.60E-06	0.000736801
Inha	270.3199716	125.1656119	560.6286909	4.479095197	2.163207329	8.57E-06	0.000736801
Tnfrsf18	119.1784898	30.73023901	296.0749914	9.634646555	3.268231743	8.48E-06	0.000736801
Cox7b	1380.800168	1696.322031	749.7564421	0.44198945	-1.17791616	8.68E-06	0.000739806
4930565N06Rik	123.6967957	33.56753632	303.9553144	9.055037923	3.178720683	9.65E-06	0.000818223
Rps15a	849.4370594	1076.584635	395.1419087	0.367032833	-1.446018969	9.87E-06	0.000831648
Gm5577	80.75459424	13.0588907	216.1460013	16.55163568	4.04890189	9.93E-06	0.00083258
Uqcr10	773.9672974	987.5838408	346.7342104	0.351093443	-1.510073044	1.00E-05	0.000836426
Nav1	2466.261041	1933.073458	3532.636209	1.827471271	0.869848726	1.02E-05	0.000848599
Rbm39	2895.10376	2299.963351	4085.384577	1.776282468	0.82886102	1.04E-05	0.00086138
Atp6v1e1	1154.079101	1431.666379	598.9045453	0.418326891	-1.257297354	1.06E-05	0.000867355
Wbp7	640.6860535	407.7178321	1106.622496	2.714187139	1.440520196	1.06E-05	0.000867355
4931430N09Rik	94.00393133	18.86089104	244.2900119	12.95219889	3.695125139	1.11E-05	0.000901606
Smap2	2257.314638	2694.192176	1383.559561	0.513534102	-0.96146801	1.23E-05	0.000994242
Ddost	2387.450381	2842.113854	1478.123436	0.520078896	-0.943197597	1.24E-05	0.000998838
Ncrna00085	163.6534571	58.04107512	374.878221	6.458843505	2.691275865	1.33E-05	0.001061652
Lpcat4	236.9220706	105.4642919	499.837628	4.739401547	2.244704899	1.35E-05	0.0010736
Scn8a	487.3484476	291.413226	879.2188908	3.017086434	1.593156028	1.44E-05	0.001137431
Tnfrsf11a	324.0356241	167.4632362	637.1803997	3.804897206	1.927857478	1.46E-05	0.001149413
Mga	3217.622249	2590.110292	4472.646163	1.726816876	0.788115097	1.53E-05	0.001193868
Atp1a1	8664.992101	9889.826502	6215.323298	0.628456252	-0.670115775	1.55E-05	0.001206084
Ndufs6	272.5132017	376.6856305	64.16834414	0.170349859	-2.553427339	1.64E-05	0.001253071
Nfasc	2402.874348	1891.467037	3425.688969	1.81112803	0.856888535	1.65E-05	0.001253071
Nrxn1	1556.885771	1171.292379	2328.072556	1.987610095	0.991034774	1.66E-05	0.001253071
Son	5191.689544	4300.491404	6974.085824	1.621695097	0.697502597	1.63E-05	0.001253071
Ssr4	1947.500427	2336.4181	1169.66508	0.500623189	-0.998202976	1.66E-05	0.001253071
Timm17a	544.0317757	710.2261838	211.6429596	0.297993744	-1.746646051	1.62E-05	0.001253071
Ghitm	6403.814598	7351.949529	4507.544736	0.61310877	-0.705785053	1.75E-05	0.001316353
Hook3	2507.831178	1984.733939	3554.025657	1.790681153	0.840508475	1.80E-05	0.001343701
Sumo3	1156.450233	1427.905634	613.5394308	0.429677855	-1.218672669	1.81E-05	0.001343701
BC006779	266.1946306	127.4208036	543.7422846	4.267295992	2.093322184	1.82E-05	0.001344302
Plekha6	2642.469704	2102.259695	3722.889721	1.770899061	0.824481983	1.85E-05	0.001365357
Smarcc2	3309.723387	2675.914139	4577.341882	1.710571283	0.774478226	1.91E-05	0.001398138
Mbd6	978.5069081	690.4227896	1554.675145	2.251772636	1.171061164	1.95E-05	0.001420211
Atat1	255.1542928	120.4292605	524.6043574	4.35612039	2.123043826	1.96E-05	0.001420678
2010107E04Rik	481.1938088	633.41852	176.7443865	0.279032553	-1.841494655	1.97E-05	0.001422433

Dmxl2	2854.792721	2285.652971	3993.072222	1.747015961	0.804892789	2.02E-05	0.00145584
Prdm2	2251.292642	1766.538677	3220.800572	1.823226751	0.866493997	2.06E-05	0.001472489
Eif2s3y	846.3030765	559.0997876	1420.709654	2.541066346	1.345434044	2.13E-05	0.001513936
Slc4a4	285.8319481	143.3676548	570.7605347	3.981096961	1.993166009	2.19E-05	0.001554403
A330023F24Rik	296.7012939	151.2284703	587.6469411	3.885822159	1.958219875	2.21E-05	0.001555815
Myeov2	273.2224108	376.6236836	66.41986499	0.176356049	-2.50343703	2.21E-05	0.001555815
Golt1b	2267.577427	2753.491017	1295.750247	0.47058452	-1.087474233	2.23E-05	0.001559353
Zfhx2	1479.767563	1113.028848	2213.244993	1.988488436	0.991672172	2.25E-05	0.001565715
Dync2h1	1311.461567	970.894375	1992.59595	2.052330306	1.03726294	2.26E-05	0.001566599
Ndufa5	160.643432	230.8333042	20.26368762	0.087784939	-3.509882741	2.27E-05	0.001566944
Ndufa2	456.2225583	602.1533266	164.3610218	0.272955433	-1.873262683	2.40E-05	0.001645658
Fam76b	409.2772988	235.6604459	756.5110046	3.210173865	1.682651437	2.46E-05	0.001678334
Tmem206	1779.102049	2137.294153	1062.71784	0.497225821	-1.008026878	2.49E-05	0.001696681
AF357425	42.11516172	1.818799505	122.7078862	67.46641718	6.076097644	2.51E-05	0.001698001
Ostc	2610.89929	3079.34606	1674.00575	0.543623782	-0.879319525	2.58E-05	0.001732547
St18	4812.606544	3989.666041	6458.48755	1.618804051	0.694928364	2.58E-05	0.001732547
Abcc10	255.5501049	123.8373797	518.9755552	4.190782755	2.067219736	2.84E-05	0.001899492
2410021H03Rik	192.0032945	79.73926347	416.5313567	5.223666969	2.385062922	2.87E-05	0.001907047
1500016L03Rik	69.7994055	10.69811288	188.0019907	17.57337886	4.135319702	2.98E-05	0.001962504
Cox6a1	1512.019127	1830.107885	875.8416095	0.478573759	-1.063186802	2.98E-05	0.001962504
Pfdn5	1055.656043	1305.984121	554.9998888	0.424966797	-1.234577968	3.00E-05	0.001962504
Rhoa	2341.72954	2772.406831	1480.374957	0.533967432	-0.905176343	3.00E-05	0.001962504
Gm3086	33.33998542	0.476519488	99.06691727	207.8968853	7.699724334	3.08E-05	0.001996247
Gnai2	5066.878258	5832.310642	3536.01349	0.606280033	-0.721943784	3.08E-05	0.001996247
Eif3d	986.0999174	1224.72802	508.8437114	0.415474867	-1.267166887	3.20E-05	0.002059309
Lamtor2	255.8089329	353.317868	60.79106287	0.172057709	-2.539035563	3.21E-05	0.002059309
Rabac1	643.9548192	824.6492957	282.5658663	0.342649739	-1.545193505	3.22E-05	0.002059309
Maged1	21055.50113	23654.52103	15857.46133	0.670377612	-0.576954125	3.32E-05	0.002115583
Luc7l2	1630.528674	1247.983921	2395.618181	1.919590583	0.94079864	3.41E-05	0.002163588
Eif3i	1555.789297	1876.625214	914.1174639	0.487107099	-1.037689086	3.73E-05	0.002355575
Rps14	1070.103394	1438.542548	333.2250854	0.231640757	-2.110038976	3.75E-05	0.002361914
Asns	4361.644749	5033.948156	3017.037935	0.599338301	-0.738557522	3.81E-05	0.002389791
Zfp445	1120.147309	821.2657603	1717.910406	2.091783792	1.064733742	4.00E-05	0.002498808
A1cf	260.0347413	128.8756936	522.3528365	4.053152474	2.019044449	4.10E-05	0.002547972
Nme1	1295.632852	1577.577141	731.7442753	0.463840567	-1.108299093	4.13E-05	0.002558093
Ppp4r1l-ps	377.6084717	216.3012158	700.2229834	3.237258658	1.694772642	4.16E-05	0.002565789
9530068E07Rik	2280.043972	2695.076245	1449.979426	0.53801054	-0.894293659	4.23E-05	0.002595107
Dpysl2	1732.685632	2074.98697	1048.082954	0.505103391	-0.985349368	4.24E-05	0.002595107
Hist1h4d	297.7060014	404.9058664	83.30627134	0.205742318	-2.281089534	4.28E-05	0.002607945
Zfp9	1483.363057	1127.991052	2194.107065	1.945145807	0.959878303	4.34E-05	0.002636423
Snrnp70	898.2326662	636.9941721	1420.709654	2.230333835	1.157259668	4.65E-05	0.002809923
1500032L24Rik	700.0585464	887.4154384	325.3447624	0.36662058	-1.447640324	4.88E-05	0.002923773
Surf4	5092.375572	5842.412602	3592.301511	0.61486611	-0.701655803	4.89E-05	0.002923773

Zcchc7	634.2945632	420.6458051	1061.592079	2.523719639	1.335551649	4.88E-05	0.002923773
Cct5	1236.78048	1507.87363	694.5941813	0.460644823	-1.118273296	4.96E-05	0.002952784
Vegfa	4479.943254	3733.272477	5973.284807	1.600013083	0.678083702	5.04E-05	0.002990147
2810407C02Rik	4677.363403	5376.375047	3279.340114	0.609953749	-0.713228244	5.11E-05	0.003016348
Prr22	34.7834255	0.953038975	102.4441985	107.4921396	6.748087356	5.25E-05	0.003089182
Unc80	4490.226046	3743.630744	5983.416651	1.598292423	0.676531387	5.58E-05	0.003267
Ctsb	7595.618513	8624.057127	5538.741284	0.642243112	-0.638808583	5.65E-05	0.003295369
Gcg	6437.569918	8148.96167	3014.786414	0.369959577	-1.434560448	5.72E-05	0.00332584
Gpc6	27.39350364	0	82.18051092	Inf	Inf	5.85E-05	0.003389026
Fbxl16	1315.452979	991.5163785	1963.326179	1.980124808	0.985591367	5.92E-05	0.003415321
Ttll10	114.7332338	34.75707906	274.6855433	7.903009999	2.982402234	5.99E-05	0.003442825
Zfp236	754.9879402	522.3197607	1220.324299	2.336354836	1.224259402	6.20E-05	0.00354946
2900005J15Rik	93.24143118	23.34594293	233.0324077	9.981708958	3.319286839	6.23E-05	0.003554073
Ikzf3	275.3225095	143.3641428	539.2392429	3.761325756	1.911241259	6.54E-05	0.003690809
Romo1	333.450785	447.2654376	105.8214798	0.236596595	-2.079498781	6.54E-05	0.003690809
Rpl38	216.5089342	301.1224324	47.28193779	0.157018982	-2.670989118	6.52E-05	0.003690809
Cox6b1	812.71709	1089.613186	258.9248974	0.237630106	-2.073210466	6.69E-05	0.003759072
Kdelr2	4334.712904	4985.107186	3033.924341	0.608597615	-0.716439416	6.92E-05	0.003877218
Akap9	4850.95605	3886.769439	6779.32927	1.744206693	0.802571013	7.04E-05	0.003926638
9130011J15Rik	1587.60084	1901.82732	959.1478808	0.504329636	-0.987561088	7.09E-05	0.003939596
B3gat2	36.22686558	1.429558463	105.8214798	74.02389099	6.209919066	7.39E-05	0.004091923
Chgb	45331.8843	39831.30066	56333.05159	1.414291039	0.500079035	7.47E-05	0.0041114
Zfp280d	723.1304097	498.737314	1171.916601	2.349767238	1.232517854	7.48E-05	0.0041114
Ndufb11	752.7061421	944.4345038	369.2494189	0.390974088	-1.354855099	8.00E-05	0.004381781
Cacna1c	928.328995	670.3181807	1444.350623	2.154723928	1.107503037	8.03E-05	0.004383741
Kcnc3	255.7027975	130.2581009	506.5921906	3.889141535	1.959451739	8.11E-05	0.004408328
Gpr116	123.2672393	41.36640501	287.068908	6.939662944	2.794865594	8.20E-05	0.004414367
Polr2j	269.621827	367.2826465	74.30018795	0.202297028	-2.305452967	8.20E-05	0.004414367
Sall1	62.90341223	94.35511834	0	0	#NAME?	8.19E-05	0.004414367
Rfx7	1219.369321	916.0622785	1825.983407	1.993296144	0.995156067	8.29E-05	0.004444825
Atxn2l	3564.834393	2700.588835	5293.325511	1.960063466	0.970900369	8.35E-05	0.004459771
Gm10804	33.2824116	0.953038975	97.94115685	102.7672104	6.683236211	8.44E-05	0.004495045
D17Wsu104e	550.2777199	707.7746155	235.2839285	0.332427758	-1.588887242	8.67E-05	0.004601629
Csf2ra	201.7046763	93.1655756	418.7828776	4.495038804	2.16833357	9.49E-05	0.005018856
Syne1	1098.962409	817.632421	1661.622385	2.032236421	1.023068248	9.56E-05	0.005035259
Ccdc162	193.2416733	87.22563368	405.2737525	4.646268939	2.216072664	0.000102502	0.00536655
Mrps14	803.9980748	1001.108715	409.7767942	0.409322972	-1.28868846	0.00010257	0.00536655
Ankrd16	223.7081564	109.8472696	451.4299298	4.10961448	2.039003062	0.000104526	0.005431431
BC005561	366.944317	216.62851	667.5759312	3.081662387	1.623708816	0.000104484	0.005431431
Glul	4214.51327	4834.640385	2974.259039	0.615197575	-0.700878279	0.000106553	0.00551785
Zfp92	935.5919879	681.2126701	1444.350623	2.120263886	1.084243833	0.000107485	0.005547198
Dclre1c	353.0553379	205.9268851	647.3122435	3.143408124	1.652329596	0.000107868	0.005548063
Dhdh	625.778481	423.6323278	1030.070788	2.431520732	1.281858892	0.000108577	0.005565664

Lphn1	1582.849503	1235.567587	2277.413337	1.84321227	0.882222226	0.000112148	0.005715499
Lrrcc1	409.9598862	250.7563322	728.366994	2.904680362	1.538379415	0.00011263	0.005715499
Pisd-ps2	140.4864015	53.12314296	315.2129186	5.93362706	2.568914254	0.000112495	0.005715499
Gm15708	25.51723627	0	76.5517088	Inf	Inf	0.000114407	0.005786301
Tpr	5227.129035	4429.639469	6822.108167	1.54010461	0.623028348	0.00011692	0.005893761
D14Abb1e	1112.692813	833.7249856	1670.628469	2.003812405	1.002747451	0.000121379	0.006098309
BC024479	133.2115213	48.96538524	301.7037935	6.161572957	2.623298696	0.000122933	0.00615596
Yod1	246.9572198	127.2715783	486.328503	3.82118702	1.934020869	0.000126799	0.006328696
Cmpk1	2132.430309	2504.614163	1388.062602	0.554202169	-0.851515737	0.00012749	0.006342321
B230206F22Rik	295.58024	164.1817749	558.3771701	3.400969263	1.765945967	0.000128505	0.006371898
Atp6v0e	981.0203647	1202.473806	538.1134824	0.447505368	-1.160023108	0.000130094	0.006429695
Evi2b	31.7813977	0.953038975	93.43811515	98.04228115	6.615332147	0.000135467	0.006673499
Rlf	689.7138915	479.5709484	1109.999778	2.314568431	1.210743217	0.000138427	0.006797283
Slc25a5	2246.645561	2630.906623	1478.123436	0.561830444	-0.831793293	0.000140396	0.0068717
Med12	1386.685294	1071.909482	2016.236919	1.880976847	0.911482091	0.000143211	0.006986941
A2ld1	278.3589763	375.8853288	83.30627134	0.221626823	-2.173795595	0.000143993	0.007002571
E030024N20Rik	108.9182815	35.04073401	256.6733766	7.324999999	2.872828759	0.000149319	0.007215341
Ggt7	234.4704889	119.2362058	464.9390549	3.89931105	1.963219244	0.000149026	0.007215341
Dad1	1893.443774	2233.380793	1213.569737	0.543377887	-0.879972239	0.000152901	0.007364973
2310016M24Rik	395.7665988	518.2239499	150.8518968	0.291094028	-1.780442855	0.000154054	0.007397015
Tmem66	2746.161887	3396.504638	1445.476384	0.425577627	-1.232505786	0.000156668	0.007498825
Kifc2	93.789618	26.41974401	228.529366	8.649946264	3.11269117	0.000157365	0.007508507
2010015L04Rik	123.0352146	43.83276893	281.4401059	6.4207695	2.682746208	0.000161306	0.007646262
Atp1b3	1025.848352	1251.14074	575.2635764	0.459791259	-1.120949055	0.000162268	0.007646262
Itsn2	905.7570125	662.915577	1391.439883	2.098969962	1.069681521	0.000161859	0.007646262
Pcf11	1280.394084	982.8326938	1875.516866	1.908276838	0.932270482	0.000161837	0.007646262
Slc25a23	1401.828709	1086.744281	2031.997564	1.869802859	0.902886169	0.00016525	0.007738718
Dvl3	934.4881156	688.0000649	1427.464217	2.074802445	1.052973975	0.000165776	0.007739431
1700110K17Rik	145.6273557	58.58305344	319.7159603	5.457482011	2.44823547	0.000166609	0.007754458
Abcc8	9727.155573	7912.159646	13357.14743	1.688179716	0.755468495	0.000169805	0.007855446
Zfp788	524.2312206	346.7373855	879.2188908	2.535691066	1.342378987	0.000169814	0.007855446
Atp5o	691.1031771	865.5391812	342.2311688	0.395396507	-1.33862797	0.000171152	0.007869345
Rpl22l1	585.9680708	742.735095	272.4340225	0.36679837	-1.446940865	0.000170912	0.007869345
6430550D23Rik	31.00179794	0.909399752	91.18659431	100.2711888	6.647763322	0.000172453	0.007905241
Scai	482.182625	313.4971434	819.5535883	2.614229844	1.386385989	0.000179788	0.008216614
Txn1	1003.886864	1224.39019	562.8802118	0.459722902	-1.121163555	0.000183309	0.008352378
Tkt	429.4814108	557.5385636	173.3671052	0.310950877	-1.685241409	0.000185044	0.008406196
Hsp90ab1	14057.50101	15688.23028	10796.04246	0.688161907	-0.539180061	0.000185824	0.00841643
Psmb1	745.4608295	927.3748524	381.6327836	0.411519444	-1.280967497	0.000186695	0.008430712
Gpatch8	1140.099341	792.6542671	1834.98949	2.314993518	1.211008154	0.000192498	0.008666998
Rps28	337.9538267	447.2654376	119.3306049	0.266800416	-1.906167178	0.000196341	0.008813862
Atp5c1	2235.448076	2636.625604	1433.093019	0.543532998	-0.879560471	0.000205123	0.009162496
Baz2b	2131.53271	1727.618832	2939.360466	1.701394087	0.766717345	0.000205315	0.009162496

Nr1d1	877.8330844	642.9820131	1347.535227	2.095758823	1.067472703	0.000210042	0.00934596
P4hb	9332.699297	10463.59834	7070.90122	0.675761912	-0.565413056	0.000211959	0.009403657
Whsc1l1	2403.408931	1964.317579	3281.591635	1.670601368	0.740367524	0.00021287	0.009416544
Ankrd11	2818.034821	2197.869067	4058.366327	1.846500498	0.884793651	0.000217595	0.009562485
D10Bwg1379e	4853.095619	4063.908779	6431.4693	1.582582103	0.662280347	0.000217575	0.009562485
Fam195b	465.3717136	624.3202627	147.4746155	0.236216289	-2.081819642	0.00021869	0.009562485
Iffo1	235.2368718	122.6373011	460.4360132	3.754453247	1.908602826	0.000218097	0.009562485
Churc1	396.3338028	516.823235	155.3549384	0.300595886	-1.734102828	0.000220284	0.009604495
Evi2a	23.64096889	0	70.92290668	Inf	Inf	0.000223675	0.009669026
Runx2	23.64096889	0	70.92290668	Inf	Inf	0.000223675	0.009669026
Srxn1	1490.202843	1773.17961	924.2493077	0.521238403	-0.939984715	0.000223131	0.009669026
Arhgap12	744.5430173	532.5448661	1168.53932	2.194255159	1.133731299	0.000227086	0.009788583
Wdr6	659.1747904	871.1202213	235.2839285	0.270093522	-1.888469058	0.000228419	0.009818127
Mtap2	1914.758485	1539.237385	2665.800683	1.731897047	0.792353172	0.000230391	0.009874919
Sfi1	282.3582662	158.9836998	529.1073991	3.328060674	1.734681735	0.000231786	0.009906714
Bclaf1	3363.903138	2673.314614	4745.080185	1.774980079	0.827802833	0.000232541	0.009911081
Eif3l	1206.838133	1456.768427	706.977546	0.485305374	-1.04303526	0.000234793	0.009979014
Solh	149.0769691	62.6317131	321.9674811	5.140646251	2.361949738	0.000238913	0.010125792
Bax	280.8048949	376.7398056	88.93507346	0.236064977	-2.08274408	0.000240528	0.01013757
Grik5	302.6832224	174.8362486	558.3771701	3.193715117	1.675235628	0.000240213	0.01013757
Cpe	32594.00207	36064.65172	25652.70277	0.711297671	-0.491474655	0.000247654	0.010380277
Cxxc1	738.9059749	529.7181047	1157.281715	2.184712407	1.127443377	0.000246992	0.010380277
Gipr	281.4715472	158.2165014	527.9816386	3.337083261	1.738587683	0.000249629	0.010434218
4932413F04Rik	52.9559206	7.94809401	142.9715738	17.98815837	4.168975586	0.000253429	0.010535006
Agrn	127.0032042	48.09611275	284.8173872	5.92183798	2.566045019	0.000253288	0.010535006
Nog	23.26571542	0	69.79714626	Inf	Inf	0.000255771	0.01060331
0610007C21Rik	973.5953856	1223.983389	472.8193779	0.386295584	-1.372222911	0.000256474	0.010603504
Airn	169.3754678	72.25289326	363.6206168	5.032609774	2.331306736	0.000258725	0.010609813
Ccdc88b	50.48793314	7.060513868	137.3427717	19.45223453	4.281863986	0.000258339	0.010609813
Ptpn13	398.474222	250.4142424	694.5941813	2.773780655	1.471853707	0.000258105	0.010609813
Zfp618	131.9119218	51.51902761	292.6977101	5.681351604	2.50623419	0.000261347	0.010688469
BC051142	53.87864313	8.206417383	145.2230946	17.69628424	4.145374559	0.000262993	0.010726877
Pax6	3069.499802	2560.431654	4087.636098	1.596463663	0.674879716	0.000270125	0.010988238
Cep350	1645.041635	1310.843618	2313.43767	1.764846423	0.819542646	0.000279156	0.011325235
Prdx3	501.9419165	640.3368325	225.1520847	0.351615077	-1.507931161	0.000283536	0.011472228
Psmb6	835.4554358	1026.342428	453.6814507	0.44203712	-1.177760572	0.00028478	0.011491939
Rps21	833.0553918	1023.305243	452.5556903	0.442248971	-1.17706931	0.000289137	0.011636817
Mll2	2536.130805	1914.607336	3779.177742	1.973865696	0.981023831	0.000292053	0.011692119
Zfp398	447.3137015	289.9006489	762.1398067	2.628968958	1.394497107	0.000291306	0.011692119
Npc2	1378.54365	1641.715155	852.2006406	0.519091657	-0.945938793	0.00029784	0.011892448
Ankhd1	3201.155129	2681.925816	4239.613755	1.58080948	0.660663504	0.000298759	0.011897826
Prss53	7565.5501	8841.256686	5014.136926	0.567129437	-0.818250053	0.000299823	0.011908933
Cadps	3166.584731	2649.771026	4200.21214	1.585122676	0.664594498	0.000302501	0.011983947

Tanc2	1532.331003	1213.263457	2170.466097	1.78894871	0.839112026	0.000314776	0.012437759
Cox6c	713.1898575	884.5971966	370.3751793	0.418693594	-1.25603325	0.000315848	0.012447694
Uimc1	422.0585706	271.1558798	723.8639523	2.669549164	1.416596119	0.000317777	0.012491252
Gabarapl1	1363.391882	1623.490544	843.1945572	0.5193714	-0.945161522	0.00032391	0.012699433
Tomm7	217.8532907	297.510165	58.53954202	0.196764847	-2.345455597	0.000326048	0.012732379
Vwa5b2	1230.383394	954.5357154	1782.07875	1.866958692	0.900690007	0.000326428	0.012732379
Map3k14	140.1265181	58.21212004	303.9553144	5.221512532	2.384467777	0.000329725	0.012827977
Prpf38b	913.3280259	680.4637794	1379.056519	2.026642065	1.01909131	0.000330997	0.012844556
Psmb10	220.0374798	300.2235685	59.66530245	0.198736238	-2.331073137	0.000333138	0.012894632
Arpc5l	917.8574896	1118.987097	515.598274	0.460772314	-1.117874062	0.00033628	0.012950202
Spcs1	1377.401026	1654.636104	822.9308696	0.497348552	-1.00767082	0.000335711	0.012950202
Rapgef6	972.4477975	731.9933429	1453.356707	1.985478039	0.989486403	0.000339769	0.013051447
Golga4	6427.309589	5558.394208	8165.140352	1.46897468	0.554809529	0.000350347	0.01342376
Bcl9	1008.891503	765.2694525	1496.135603	1.955044198	0.967201223	0.000365463	0.013905205
Psmd8	712.1409072	882.460891	371.5009398	0.420982894	-1.248166482	0.000365661	0.013905205
Smg1	2186.936473	1791.586549	2977.63632	1.66201087	0.732929818	0.0003652	0.013905205
Atox1	346.7473263	454.2640046	131.7139696	0.289950267	-1.786122627	0.000376609	0.014250102
Celsr3	858.9289187	638.2667335	1300.253289	2.037162868	1.026561326	0.000375867	0.014250102
Atp6v1b2	3451.60437	3943.01025	2468.792609	0.62611874	-0.675491813	0.000378227	0.014275718
Pion	157.448117	70.6853932	330.9735645	4.682347364	2.227231966	0.000379817	0.014300162
Adi1	4677.014425	5295.35971	3440.323854	0.649686526	-0.62218431	0.000382109	0.01435084
Mdn1	1031.257631	785.8724005	1522.028093	1.93673692	0.953627996	0.000384277	0.014368374
Ndufa13	656.2767273	817.239668	334.3508458	0.409122145	-1.289396466	0.00038447	0.014368374
Slc25a39	1090.792858	1313.096046	646.1864831	0.492109077	-1.022949967	0.00038818	0.014471404
Dstn	3447.707503	3938.290711	2466.541088	0.626297363	-0.67508029	0.000397218	0.014772025
Srsf5	1754.435378	1413.017408	2437.271317	1.724869986	0.786487621	0.000398645	0.014788857
AW549877	1289.376304	1008.689388	1850.750136	1.834806789	0.87562815	0.000410103	0.015176796
Nbea	2801.377968	2340.059211	3724.015481	1.591419338	0.670314035	0.000414807	0.015276388
Prpf4b	1951.959458	1589.972923	2675.932527	1.683005092	0.751039542	0.000413996	0.015276388
Laptm4a	1618.280495	1905.067906	1044.705673	0.54838238	-0.866745877	0.000417648	0.015343752
Appl2	673.7196046	483.1606484	1054.837517	2.183202461	1.126445926	0.000422857	0.015434151
Fam178a	1708.307217	1375.909339	2373.102973	1.724752427	0.786389291	0.000422743	0.015434151
Pex5l	639.6020937	453.9367104	1010.93286	2.22703482	1.155124115	0.00042316	0.015434151
Manf	1808.547651	2116.731333	1192.180289	0.563217575	-0.828235741	0.000434955	0.015819649
Prnd	37.42485217	3.226538356	105.8214798	32.79721737	5.035501511	0.000435815	0.015819649
1700029J07Rik	161.8634803	74.49403714	336.6023666	4.518514227	2.175848465	0.000448383	0.016209421
Rps6kb2	246.8180858	137.194721	466.0648153	3.397104581	1.764305633	0.000448689	0.016209421
Chchd2	658.3810546	817.5817579	339.9796479	0.415835658	-1.265914621	0.000469585	0.016924027
Mll5	3155.695394	2659.329511	4148.427161	1.55995229	0.641501906	0.000477682	0.017141462
Sdf2l1	1237.150686	1475.219005	761.0140463	0.515865132	-0.95493416	0.000477878	0.017141462
Mrfap1	5077.086838	6082.90744	3065.445633	0.503944152	-0.988664234	0.000482878	0.017279968
Rpl22	1128.919362	1352.836516	681.0850562	0.50344964	-0.990080623	0.000488305	0.017393076
Uba7	135.9806186	57.0591926	293.8234705	5.149450196	2.364418405	0.000488331	0.017393076

Ica1l	356.9559138	223.5982334	623.6712746	2.789249562	1.479877022	0.000493727	0.017544058
Hivep1	1405.956039	1114.324725	1989.218668	1.785133745	0.836032167	0.000498858	0.017684984
Dus3l	209.0219959	109.207477	408.6510337	3.741969369	1.903797749	0.000505869	0.017880046
Rer1	948.1427097	1148.091401	548.2453263	0.477527595	-1.066343989	0.00050744	0.017880046
Whamm	273.1799672	158.7253764	502.0891489	3.163256942	1.661410747	0.000507896	0.017880046
Myt1	910.884993	687.4939541	1357.667071	1.974805833	0.981710812	0.000511283	0.017957622
1810046J19Rik	1171.629764	1442.794608	629.3000768	0.436167472	-1.197045913	0.00051498	0.01802971
BC031181	952.4015143	1152.228087	552.748368	0.47972131	-1.059731566	0.000515712	0.01802971
Fryl	2040.770487	1673.656008	2774.999444	1.658046475	0.729484446	0.000518771	0.018036776
Hap1	1443.756477	1120.929043	2089.411346	1.863999652	0.898401591	0.000518773	0.018036776
Maml3	948.419958	719.5925525	1406.074769	1.953987384	0.966421152	0.000520616	0.018036776
Znf512b	799.2369739	592.6334727	1212.443976	2.045858076	1.032706067	0.000520669	0.018036776
Epcam	1621.133607	1904.281652	1054.837517	0.55392936	-0.852226087	0.000522356	0.018054016
Itgb2l	31.66625007	1.90607795	91.18659431	47.83990827	5.580142718	0.000524893	0.018059418
Mir665	41.0628511	4.743375265	113.7018028	23.97065305	4.583197309	0.000523976	0.018059418
Nedd8	530.5532954	668.6190152	254.4218557	0.380518427	-1.393961777	0.00053164	0.018250166
Ush2a	927.4775346	701.1251623	1380.182279	1.968524813	0.977114898	0.000535073	0.018326555
Crip1	197.5033945	270.362602	51.78497948	0.191538989	-2.384290002	0.000537396	0.018364651
Cep290	1085.302475	838.795655	1578.316114	1.881645553	0.911994892	0.000541629	0.018385085
Runx1t1	1614.302253	1299.633118	2243.640524	1.726364535	0.787737133	0.000541607	0.018385085
Tmed3	1715.759039	2009.632586	1128.011944	0.561302574	-0.833149418	0.000539456	0.018385085
BC065397	43.45810653	5.521857349	119.3306049	21.61059175	4.433666672	0.000545739	0.018483266
Luc7l3	1473.959255	1178.053694	2065.770377	1.75354518	0.810274602	0.00054705	0.018486393
F8a	227.3689221	307.2805704	67.54562541	0.21981743	-2.185622306	0.000551724	0.018602912
Tbca	825.4011635	1006.757978	462.6875341	0.459581691	-1.12160677	0.000557131	0.018743566
Chd3	522.4277092	359.7927643	847.6975989	2.356071836	1.236383528	0.000559521	0.018782335
Rcbtb2	307.0383767	185.3091414	550.4968471	2.970694499	1.570800249	0.000564548	0.018909246
Khdrbs2	21.01419457	0	63.04258372	Inf	Inf	0.000571753	0.019108376
Ndufb8	615.7630674	813.8829599	219.5232826	0.269723404	-1.890447383	0.000579175	0.019313885
Alkbh7	147.8778481	207.1818867	29.26977101	0.141275724	-2.823414514	0.000584317	0.019400077
Ptpn2	652.1793741	469.4253498	1017.687423	2.167943046	1.116326856	0.00058336	0.019400077
Rufy3	1153.91926	900.6305784	1660.496625	1.843704472	0.882607424	0.00059804	0.019791029
Tspan31	1191.429362	1420.146145	733.9957961	0.516845255	-0.952195698	0.0005987	0.019791029
Lrrc4	194.1739491	79.05508376	424.4116797	5.368556448	2.424534214	0.000610591	0.020140223
Kcnab1	41.6830522	5.110796695	114.8275632	22.46764449	4.489776975	0.000615647	0.020206063
Mrpl46	172.2068286	238.0465553	40.52737525	0.170249787	-2.554275105	0.000617935	0.020206063
Pdia6	3177.050946	3629.684151	2271.784535	0.625890419	-0.676018003	0.000617577	0.020206063
Rpl36al	1398.515829	1651.972616	891.6022554	0.539719755	-0.8897176	0.000614025	0.020206063
Spag9	1168.436595	879.0648034	1747.180177	1.98754423	0.990986966	0.000619246	0.020206063
Ampd3	26.88855612	0.931219364	78.80322965	84.62370168	6.40298989	0.000622387	0.020264985
Cstb	763.4571514	935.2314082	419.908638	0.448989025	-1.155247915	0.000627917	0.020401256
Rps25	1104.27209	1320.93153	670.9532124	0.507939433	-0.977271615	0.000635685	0.020609513
Cd300lf	20.6389411	0	61.91682329	Inf	Inf	0.000653768	0.021105565

Sema6d	330.946817	205.9740363	580.8923785	2.820221369	1.495808409	0.000652498	0.021105565
Aldoa	5192.717537	6099.872789	3378.407031	0.553848768	-0.852436001	0.000656669	0.021124089
Gabbr1	146.1321152	66.09475522	306.2068352	4.632846195	2.211898787	0.000657126	0.021124089
Aak1	3365.0818	2623.297627	4848.650144	1.848303484	0.886201661	0.000660847	0.021198797
Krba1	259.9112952	150.6428528	478.44818	3.176043013	1.667230451	0.000679616	0.021754887
Akap8	779.2412833	578.4005828	1180.922684	2.041703828	1.029773603	0.000681348	0.021764404
Tnrc6b	2366.995621	1969.362917	3162.26103	1.605727925	0.683227462	0.000684216	0.021810089
Lman2	3657.653258	4151.328025	2670.303725	0.64324084	-0.636569088	0.000687244	0.021860689
Cox8a	1518.438619	1783.449103	988.4176519	0.554216911	-0.851477363	0.000706031	0.022364515
Ccnt2	596.4629487	426.3780868	936.6326724	2.196718596	1.13535007	0.000743902	0.023515057
Fkbp14	184.7473733	94.74787135	364.7463772	3.849652473	1.944728212	0.000756266	0.023757389
Ptbp2	184.4326468	94.83866176	363.6206168	3.834096876	1.93888679	0.000754277	0.023757389
Ssh3	144.1534112	65.37822001	301.7037935	4.614744688	2.206250833	0.000755994	0.023757389
Safb2	559.1832936	394.6624534	888.2249742	2.250594062	1.170305863	0.000758023	0.0237634
Senp7	663.766432	462.6020875	1066.095121	2.304561847	1.204492485	0.000761311	0.02381727
Zmym5	762.9349799	566.8873725	1155.030195	2.037495013	1.026796528	0.000766089	0.023917416
BC018242	242.7182727	138.3624442	451.4299298	3.262662296	1.706049668	0.000769702	0.023980865
Sdcbp	2121.570664	2452.300362	1460.111269	0.595404744	-0.748057378	0.000773121	0.024038045
Atp6v0d1	2125.116808	2455.930937	1463.488551	0.595899717	-0.746858532	0.00077602	0.024052844
D8Ertd738e	277.4724453	366.6752093	99.06691727	0.270176207	-1.888027465	0.00077747	0.024052844
Nsd1	3903.239117	3346.664452	5016.388447	1.498921843	0.58392516	0.000778353	0.024052844
Hirip3	303.8164233	185.5421332	540.3650033	2.912357392	1.542187408	0.000786068	0.024241879
Sil1	442.923922	563.0674449	202.6368762	0.359880292	-1.474410998	0.000794428	0.024450007
Mapk15	219.9567632	121.6694664	416.5313567	3.423466618	1.775457945	0.000803466	0.024648391
Tmem86b	76.26652275	22.0874294	184.6247095	8.358813792	3.063298222	0.000804123	0.024648391
4833439L19Rik	4037.965552	4560.812725	2992.271206	0.656082893	-0.608049991	0.000813269	0.02487848
Stard8	87.35222925	28.58414533	204.8883971	7.16790356	2.841551227	0.000816388	0.024923663
Sec61b	930.1985269	1173.522987	443.5496069	0.37796414	-1.403678731	0.000818464	0.024936869
Vldlr	3406.633258	2906.836738	4406.226298	1.515814851	0.600093547	0.000831882	0.025294889
Psma2	1095.405043	1307.068077	672.0789728	0.514188193	-0.959631612	0.000842252	0.025558978
Nkrf	285.73374	376.2527502	104.6957194	0.278259014	-1.84549967	0.00084659	0.025639333
Atp5a1	5804.75574	6810.790177	3792.686867	0.556864441	-0.844601923	0.000857639	0.025870698
Pcdhgc5	37.64131879	4.114118497	104.6957194	25.44791052	4.669475299	0.000856268	0.025870698
Ptpmt1	145.9927287	203.7913273	30.39553144	0.14915027	-2.745161508	0.000863764	0.026003753
E130307A14Rik	40.22567752	5.176255529	110.3245215	21.31357714	4.413700841	0.000872731	0.026221675
Spnb3	525.63554	368.544672	839.817276	2.278739431	1.188235965	0.000876622	0.026286546
Slc8a1	3483.224866	2976.693734	4496.287132	1.51049706	0.595023376	0.000886071	0.026517478
C430048L16Rik	209.0390955	113.7361681	399.6449504	3.513789475	1.813027756	0.000898035	0.026822616
1700034H15Rik	29.73179707	1.818799505	85.55779219	47.04080464	5.555840833	0.000901572	0.026875333
Zfml	1533.150333	1240.38494	2118.681117	1.708083554	0.772378549	0.000910068	0.027075409
Ndufb2	319.5876039	416.3388221	126.0851674	0.302842687	-1.723359523	0.000918555	0.027274428
2900010M23Rik	485.9971948	611.9167081	234.1581681	0.382663465	-1.385851928	0.000923456	0.027366411
Ins1	2373536.279	2590036.277	1940536.285	0.749231315	-0.416516896	0.000930546	0.027522748

BC018507	976.3485853	754.1680507	1420.709654	1.883810449	0.913653807	0.000937625	0.027644699
Tmem146	67.0886237	17.88954441	165.4867823	9.250474938	3.209527438	0.000938313	0.027644699
Atp6v1f	476.8921888	634.2835327	162.109501	0.255578921	-1.96815924	0.000947641	0.027859186
Zfp692	106.0492187	41.43186385	235.2839285	5.678815932	2.50559015	0.000949265	0.027859186
C030046E11Rik	457.2330913	312.0971763	747.5049212	2.395103122	1.260087773	0.000962917	0.028150931
Etl4	1203.492025	918.7017036	1773.072667	1.929976466	0.948583255	0.000961991	0.028150931
Dusp3	1062.966636	1267.979431	652.9410456	0.514946086	-0.957506702	0.000967131	0.028219777
Hint1	679.9808002	834.7836106	370.3751793	0.443678068	-1.172414856	0.0009723	0.028316135
Rplp0	897.1549895	1081.178785	529.1073991	0.489380116	-1.03097261	0.000975463	0.028353845
Klhl20	287.2233684	175.2874365	511.0952323	2.915755073	1.543869537	0.000980898	0.028402975
Mll1	3636.861026	2663.968569	5582.64594	2.09561254	1.067372	0.00097906	0.028402975
Hnrnph1	2592.480979	2183.757307	3409.928323	1.56149601	0.642928883	0.00098806	0.028555889
Fkbp1a	3277.474013	3755.552023	2321.317993	0.618103006	-0.694080815	0.00099458	0.028580993
Gnb2	373.5084715	479.7708369	160.9837406	0.335542989	-1.575430483	0.000991953	0.028580993
Map4k3	764.0883155	571.9946573	1148.275632	2.007493632	1.005395411	0.000994401	0.028580993
Akr1a1	2549.077547	2916.816299	1813.600042	0.62177383	-0.685538198	0.001002252	0.028730874
Phldb2	1375.275568	1104.328351	1917.170001	1.736050695	0.795809077	0.001003583	0.028730874
Zfp169	359.6128166	233.2123896	612.4136704	2.625991146	1.392862052	0.001012249	0.028924395
9330159F19Rik	211.4513446	116.7916615	400.7707108	3.431501064	1.778839802	0.001031272	0.029357397
Cblb	864.1363227	659.5869645	1273.235039	1.930352035	0.948863973	0.001029859	0.029357397
Kidins220	4387.981643	3795.152536	5573.639857	1.468620775	0.554461913	0.001035547	0.029423896
Atxn7l3b	1088.126778	1295.02492	674.3304937	0.520708508	-0.941452116	0.001040059	0.029496843
Akap13	6731.80557	5909.316699	8376.783311	1.417555318	0.503405035	0.001043121	0.029528505
2410015M20Rik	416.9733796	530.3333136	190.2535116	0.358743278	-1.478976294	0.001047285	0.029591151
Snx3	891.0935481	1073.212383	526.8558782	0.490914833	-1.026455335	0.001064229	0.029958341
Srrm1	2324.150683	1947.874405	3076.703237	1.579518284	0.659484637	0.001062643	0.029958341
Mipep	197.8278789	268.0349275	57.4137816	0.214202612	-2.222952022	0.001067653	0.029999085
Lair1	40.52881077	5.630955406	110.3245215	19.59250492	4.292229954	0.001083877	0.030398642
Lpp	4942.364466	4296.316086	6234.461225	1.451117911	0.537164751	0.001086948	0.030428524
Dcp1a	806.0521858	609.6108532	1198.934851	1.96672163	0.975792773	0.001090804	0.030480247
Nemf	1210.220006	961.4407278	1707.778562	1.776270251	0.828851097	0.001095803	0.030546626
Polr2g	628.1014976	773.8510631	336.6023666	0.434970478	-1.201010609	0.001097206	0.030546626
Atxn1	935.8649823	721.5866567	1364.421633	1.89086317	0.919044968	0.001104759	0.03059486
Cobra1	405.9622397	271.7781126	674.3304937	2.48118028	1.311026564	0.001104988	0.03059486
Rpl29	806.9978195	977.4643216	466.0648153	0.476810053	-1.06851344	0.001104889	0.03059486
Spred3	210.5553736	116.5734654	398.5191899	3.418609789	1.773409758	0.0011111	0.030708046
Trpm7	590.398978	426.8510944	917.4947452	2.149449204	1.103967017	0.001113671	0.030723154
Smarca2	2289.935025	1920.191888	3029.4213	1.577665919	0.657791738	0.001127939	0.031060295
Tspan7	2176.658952	2502.285741	1525.405374	0.60960479	-0.714053856	0.001138541	0.031238854
Usp34	3289.756093	2813.701501	4241.865276	1.507574728	0.592229516	0.00113656	0.031238854
Gm561	224.1385166	300.1834413	72.04866711	0.240015461	-2.058800751	0.001146883	0.031410943
Ift52	384.1849683	492.9711812	166.6125427	0.337976233	-1.565006298	0.00115777	0.031595054
Mgat4b	103.8348367	148.4348122	14.63488551	0.098594698	-3.342346121	0.001155947	0.031595054

Phip	2403.192998	2020.844582	3167.889832	1.567606861	0.648563793	0.001162033	0.03165444
Hn1l	1455.556295	1703.760502	959.1478808	0.562959336	-0.828897379	0.001168744	0.031729216
Rsrc2	1469.670975	1192.447841	2024.117242	1.697447194	0.763366694	0.00116896	0.031729216
Mrpl51	286.8565473	375.6854403	109.1987611	0.290665406	-1.782568717	0.001175368	0.031846167
Gpx3	827.2588918	630.7261882	1220.324299	1.934792501	0.952178851	0.001188227	0.032080015
Nop10	581.1241079	719.1456244	305.0810748	0.424227117	-1.237091253	0.001186629	0.032080015
Eef2	7586.148988	8423.53949	5911.367984	0.701767706	-0.510934536	0.001202423	0.032405609
Chd9	3161.922	2702.442233	4080.881535	1.510071699	0.594617051	0.001216801	0.032447306
Dennd2d	293.7170253	182.2135207	516.7240344	2.835816092	1.503763974	0.001214369	0.032447306
Eno3	121.6242442	52.97391764	258.9248974	4.887780797	2.289179586	0.001216493	0.032447306
Sfswap	335.3083834	215.8936671	574.137816	2.659354597	1.411076158	0.001210717	0.032447306
Snx32	198.5067643	108.6323953	378.2555023	3.481977003	1.799906675	0.001207447	0.032447306
Tmem14c	278.6988069	365.7003507	104.6957194	0.286288266	-1.804459553	0.001213963	0.032447306
Chdh	24.65158169	0.953038975	72.04866711	75.59886739	6.240292715	0.001241935	0.033059441
Ndufb10	601.8131553	742.8617529	319.7159603	0.430384199	-1.216302984	0.001246519	0.033123357
Polr3a	462.1414089	319.4596528	747.5049212	2.339904006	1.226449345	0.001252734	0.033230315
Fam135b	612.4502668	446.9817827	943.3872349	2.110572	1.077634046	0.001277371	0.033824692
Fam193b	286.8389689	178.0881185	504.3406697	2.831972587	1.5018073	0.001286606	0.033971282
Nenf	402.6908311	512.2867721	183.498949	0.358195759	-1.48117984	0.001287385	0.033971282
Rnaseh2a	74.13317115	108.3853557	5.628802118	0.051933235	-4.267198101	0.001310536	0.034462324
Vps35	2635.83217	3004.169338	1899.157834	0.632174029	-0.661606327	0.001308706	0.034462324
LOC626693	30.06402313	2.317138604	85.55779219	36.92389918	5.206483005	0.001323688	0.034696897
Mtch1	1311.408201	1557.898388	818.4278279	0.525340956	-0.928674033	0.001325731	0.034696897
Tmtc3	2131.232339	1784.019177	2825.658663	1.58387236	0.663456077	0.001326316	0.034696897
Ngfrap1	3107.619285	3521.596499	2279.664858	0.647338461	-0.627407872	0.001333375	0.0348215
Trim46	253.6908789	152.0069524	457.058732	3.006827811	1.588242253	0.001374555	0.035835275
Lrrc27	243.4702152	143.9933996	442.4238464	3.072528656	1.619426466	0.001380863	0.035937978
Mir344c	24.2472354	0.909399752	70.92290668	77.98870243	6.285193243	0.001400975	0.03639895
Chd8	1166.661756	929.3132859	1641.358698	1.766205996	0.820653617	0.001404461	0.036427145
Eef1g	913.5236553	1094.474179	551.6226075	0.504006963	-0.988484429	0.001407257	0.036437394
Pard6b	158.7677526	218.4508214	39.40161482	0.180368353	-2.470981868	0.001414164	0.036531508
Unc50	1010.872604	1203.910388	624.7970351	0.518973041	-0.946268498	0.001415707	0.036531508
Atp5g3	1559.828839	1859.606438	960.2736413	0.516385414	-0.953479843	0.001441708	0.037046485
Ids	11227.51458	9903.773266	13874.99722	1.400980904	0.486437291	0.001442989	0.037046485
Rimbp2	1171.893947	933.7842899	1648.11326	1.764982853	0.819654168	0.001439192	0.037046485
Odf2	706.4916651	530.0672184	1059.340559	1.998502306	0.998919238	0.001448095	0.037114767
Svil	769.9844281	566.2039406	1177.545403	2.079719547	1.056388992	0.001479557	0.0378572
Jmjd1c	2977.502157	2545.705953	3841.094565	1.508852411	0.593451695	0.001485792	0.037952743
Samm50	495.3736683	618.6639757	248.7930536	0.402145694	-1.314209824	0.001502712	0.038320421
Fam126b	864.6066949	666.4842052	1260.851674	1.891795281	0.919755977	0.001519757	0.038690066
Ext2	1701.16078	1404.591299	2294.299743	1.633428702	0.707903484	0.001526657	0.038800624
Idh3a	443.6596706	558.5422657	213.8944805	0.382951289	-1.384767201	0.001536353	0.038975129
Luc7l	671.11157	499.5122842	1014.310142	2.030600996	1.021906784	0.001539187	0.038975129

Vamp8	677.6090253	826.7229066	379.3812627	0.458897727	-1.123755434	0.00154123	0.038975129
Lrrc45	116.2105792	50.48222215	247.6672932	4.906029938	2.294556038	0.00154585	0.039026925
Fyco1	1147.410893	915.0718764	1612.088926	1.761707433	0.816974356	0.001551281	0.039080843
Hspe1	564.5596161	697.676168	298.3265122	0.427600262	-1.225665358	0.001553137	0.039080843
Atn1	998.8781013	784.5850434	1427.464217	1.819387495	0.863452843	0.001563207	0.0392691
Lsmd1	181.4157619	246.2311531	51.78497948	0.210310429	-2.249407701	0.001576995	0.039549983
Herpud1	2303.723151	2633.216737	1644.735979	0.624610939	-0.678970259	0.001587192	0.039674569
Tpi1	662.8254121	817.4937316	353.488773	0.432405484	-1.209543273	0.001586422	0.039674569
Trim39	425.0291134	293.0609805	688.9653792	2.35092839	1.233230595	0.001594373	0.039788522
Carhsp1	326.492525	421.0674017	137.3427717	0.326177641	-1.616270205	0.001606091	0.040015129
Chchd10	1104.786266	1306.505027	701.3487439	0.536812893	-0.897508772	0.00162463	0.040362195
Rock1	1863.231807	1549.756682	2490.182057	1.606821307	0.684209497	0.001625341	0.040362195
BC005764	202.3457299	90.74987483	425.5374401	4.689124265	2.229318512	0.001630711	0.040379748
Ccnd2	10390.013	11455.16739	8259.704227	0.72104614	-0.471836515	0.001631371	0.040379748
Smek2	1360.989577	1102.037292	1878.894147	1.704927919	0.769710746	0.001646505	0.04065768
Zfp706	790.4602164	969.5443233	432.2920026	0.445871315	-1.165300707	0.001647958	0.04065768
Sec63	2767.114371	2359.586722	3582.169668	1.518134356	0.602299476	0.001654953	0.040763976
Snap23	1201.307606	962.7070131	1678.508791	1.743530242	0.802011388	0.001688332	0.041518744
0610011F06Rik	559.4268035	691.1027095	296.0749914	0.428409536	-1.2229375	0.001695439	0.041626049
Lmna	405.1610466	512.6148141	190.2535116	0.371143218	-1.429952089	0.001705454	0.041804294
Atp6v1c1	1493.012841	1737.430113	1004.178298	0.577967592	-0.790939496	0.001718365	0.042052834
Zfp407	418.6472512	288.554109	678.8335354	2.352534634	1.234215963	0.001721622	0.042064679
Bcar1	550.0477768	679.8485705	290.4461893	0.427221887	-1.226942535	0.001730303	0.042073535
Cisd1	674.0296119	821.3537865	379.3812627	0.461897503	-1.114355349	0.00172703	0.042073535
Nipbl	2474.650814	2098.761534	3226.429374	1.537301557	0.620400192	0.001729335	0.042073535
Mettl9	1050.404815	1245.196539	660.8213686	0.530696439	-0.914041227	0.001734631	0.0421113
Cltb	813.4388791	978.6827078	482.9512217	0.49347068	-1.018963726	0.001767974	0.042852185
Cdc42	2305.889123	2632.525534	1652.616302	0.627768385	-0.671695721	0.001772629	0.042896485
Hivep3	513.4139153	368.2244017	803.7929424	2.182888854	1.126238675	0.001776069	0.042911301
Ezh1	738.7828394	559.9289328	1096.490653	1.958267538	0.969577879	0.00177979	0.04293283
Araf	1842.659448	1532.970149	2462.038046	1.606057396	0.683523452	0.001789203	0.043091403
Plekhn1	109.3958896	46.45187013	235.2839285	5.065112079	2.340594193	0.001794346	0.043146773
Ndufb3	306.075184	396.0701922	126.0851674	0.318340461	-1.651357562	0.001797277	0.043148871
Hspa8	2514.577212	2861.688516	1820.354605	0.63611207	-0.652647134	0.001804456	0.043252782
Dido1	1115.78254	890.7074356	1565.932749	1.758077553	0.813998713	0.001819422	0.043542733
Rps17	572.1499406	704.5586131	307.3325956	0.43620586	-1.196918944	0.001832148	0.043778235
Psmd13	337.3323781	432.8241396	146.3488551	0.338125446	-1.564369505	0.00185253	0.044195664
Uqcr11	324.4188008	417.3939352	138.4685321	0.331745434	-1.591851487	0.001861272	0.0443345
Ift46	209.769694	280.3188481	68.67138584	0.244975985	-2.029287768	0.001868454	0.044435827
Hadh	3364.63427	3790.602773	2512.697265	0.662875383	-0.593190418	0.00187335	0.044482545
Commd1	246.102127	324.1227735	90.06083388	0.277860247	-1.84756865	0.001894176	0.044697239
Mtss1l	1466.189951	1201.298311	1995.973231	1.661513392	0.732497921	0.00189235	0.044697239
Pitpnm2	732.7104238	551.3831897	1095.364892	1.986576509	0.990284357	0.001887363	0.044697239

Zfp760	272.1681547	169.5910223	477.3224196	2.814550046	1.492904301	0.001893888	0.044697239
6330407A03Rik	17.6369133	0	52.91073991	Inf	Inf	0.001909502	0.044919193
Cib1	138.222341	191.5728656	31.52129186	0.164539439	-2.603494664	0.001907048	0.044919193
Sec16b	455.0268644	320.6083204	723.8639523	2.257782803	1.174906707	0.001933959	0.045424091
Hyou1	8555.516144	9591.084196	6484.38004	0.676084153	-0.564725262	0.001959345	0.045949212
Hic2	125.6225503	58.40849655	260.0506578	4.452274467	2.154542532	0.001977283	0.046291597
Trappc3	590.0193263	724.0452489	321.9674811	0.444678674	-1.169164878	0.001980047	0.046291597
Tspyl2	1340.339019	1089.636502	1841.744053	1.690237111	0.757225646	0.001990742	0.04647005
Tmco1	777.8762881	937.1593057	459.3102528	0.490109045	-1.028825321	0.002012185	0.046898446
Cenpt	58.28313094	15.37602931	144.0973342	9.371556942	3.22828875	0.002021683	0.047047546
Atp5g1	920.4261292	1096.947567	567.3832535	0.517238262	-0.951099093	0.00203175	0.047209409
0610011L14Rik	291.1386589	377.6055661	118.2048445	0.313037876	-1.675590867	0.002064051	0.047644865
AI450353	51.84430667	11.90947523	131.7139696	11.05959474	3.467226616	0.002066191	0.047644865
Gm3414	221.2714389	130.3960425	403.0222316	3.090755086	1.627959339	0.002062854	0.047644865
Nfkb1	575.1478759	390.4653162	944.5129953	2.418942109	1.274376242	0.002064607	0.047644865
Tm4sf4	1992.097086	2395.432765	1185.425726	0.494869129	-1.014881049	0.002057845	0.047644865
Tprgl	559.7711287	689.3676765	300.5780331	0.436019911	-1.197534077	0.00207039	0.047669245
Hscb	164.2187507	223.8129176	45.03041694	0.201196684	-2.313321564	0.002074216	0.047684969
2700038G22Rik	26.74431568	1.840619116	76.5517088	41.59019545	5.37817156	0.002081286	0.047775112
1300002E11Rik	629.4849262	469.1564905	950.1417975	2.025212944	1.01807361	0.002099617	0.048123093
Cfp	57.09857307	14.72495293	141.8458134	9.633023212	3.267988642	0.002109852	0.048212019
Dhrs7b	116.7658002	163.8910961	22.51520847	0.137379083	-2.863765736	0.002109282	0.048212019
Ccdc47	4859.92733	5418.31429	3743.153408	0.690833571	-0.533589903	0.002118424	0.04833512
Atp5j	1260.646689	1475.001557	831.936953	0.564024458	-0.826170372	0.002127738	0.048474728
Wapal	2088.982965	1761.735371	2743.478152	1.557258938	0.639008853	0.002134971	0.04856659
Cltc	9046.104038	9968.411342	7201.489429	0.722431006	-0.469068283	0.00213941	0.04859472
Ahsa1	570.5917528	701.0955709	309.5841165	0.441571919	-1.179279666	0.002154408	0.048852832
Ndufv3	682.7556739	828.8140773	390.638867	0.471322674	-1.085213009	0.002157213	0.048852832
Ccdc39	17.26165983	0	51.78497948	Inf	Inf	0.002183067	0.049364638
Dmtf1	418.6072828	291.8714378	672.0789728	2.302654134	1.20329773	0.002203911	0.049761809
Sec61a1	4296.264659	4801.91253	3284.968916	0.68409595	-0.547729405	0.002217321	0.049990207

This table lists a complete set of genes differentially expressed between $ALDH^{-}$ and $ALDH^{+}$ cells, arranged by p-value.

Supplementary Table 2 Curated list of differentially expressed transcripts in wild-type ALDH⁻ vs. ALDH⁺ cells

Gene	ALDH⁻	ALDH⁺	Fold change	Log_2 Fold Change	p-value	Adjusted p-value	Function
Aldh1a3	43.89	1016.56	23.16	4.53	6.71E-24	1.13E-20	Biomarker
Ins1	2590036.28	1940536.28	0.75	-0.42	0.000930546	0.027522748	Hormone production
Gcg	8148.96	3014.79	0.37	-1.43	5.72E-05	0.00332584	
Ppy	2650.81	496.46	0.19	-2.42	1.23E-21	1.55E-18	
Pyy	5362.39	860.08	0.16	-2.64	3.61E-37	1.10E-33	
Gipr	158.22	527.98	3.34	1.74	0.000249629	0.010434218	
Malat1	28383.66	317323.72	11.18	3.48	3.39E-60	5.14E-56	LncRNA
Meg3	1394.28	14866.79	10.66	3.41	3.32E-52	2.52E-48	
Neat1	1293.07	8685.24	6.72	2.75	1.34E-30	2.91E-27	
Peg3	10847.19	26432.85	2.44	1.29	6.41E-22	9.35E-19	
Snhg11	142.49	2397.86	16.82	4.07	4.52E-43	1.71E-39	
Kcnq1ot1	775.61	5750.38	7.41	2.89	1.90E-30	3.61E-27	
Bach2	16.18	238.66	14.75	3.88	5.61E-06	0.000512996	Cellular differentiation
Mlxipl	1521.05	6819.86	4.48	2.16	1.35E-33	3.42E-30	
Hic2	58.41	260.05	4.45	2.15	0.001977283	0.046291597	
Ncor1	3821.50	6625.10	1.73	0.79	1.41E-06	0.000162812	
Pax6	2560.43	4087.64	1.60	0.67	0.000270125	0.010988238	
Rfx6	1409.36	2881.95	2.04	1.03	1.46E-06	0.000166394	
Rfx7	916.06	1825.98	1.99	1.00	8.29E-05	0.004444825	
Sall1	94.36	0.00	0.00	INF	8.19E-05	0.004414367	
Atp1a1	9889.83	6215.32	0.63	-0.67	1.55E-05	0.001206084	Complex V
Atp1b3	1251.14	575.26	0.46	-1.12	0.000162268	0.007646262	
Atp5a1	6810.79	3792.69	0.56	-0.84	0.000857639	0.025870698	
Atp5c1	2636.63	1433.09	0.54	-0.88	0.000205123	0.009162496	
Atp5e	1589.03	326.47	0.21	-2.28	4.43E-14	3.05E-11	
Atp5g1	1096.95	567.38	0.52	-0.95	0.00203175	0.047209409	
Atp5g3	1859.61	960.27	0.52	-0.95	0.001441708	0.037046485	
Atp5j	1475.00	831.94	0.56	-0.83	0.002127738	0.048474728	
Atp5o	865.54	342.23	0.40	-1.34	0.000171152	0.007869345	
Atp6v0d1	2455.93	1463.49	0.60	-0.75	0.00077602	0.024052844	
Atp6v0e	1202.47	538.11	0.45	-1.16	0.000130094	0.006429695	
Atp6v1b2	3943.01	2468.79	0.63	-0.68	0.000378227	0.014275718	
Atp6v1c1	1737.43	1004.18	0.58	-0.79	0.001718365	0.042052834	
Atp6v1e1	1431.67	598.90	0.42	-1.26	1.06E-05	0.000867355	
Atp6v1f	634.28	162.11	0.26	-1.97	0.000947641	0.027859186	
Cox17	750.09	184.62	0.25	-2.02	6.45E-07	8.30E-05	Complex IV

Cox4i1	1686.10	526.86	0.31	-1.68	1.82E-09	4.67E-07	
Cox6a1	1830.11	875.84	0.48	-1.06	2.98E-05	0.001962504	
Cox6b1	1089.61	258.92	0.24	-2.07	6.69E-05	0.003759072	
Cox6c	884.60	370.38	0.42	-1.26	0.000315848	0.012447694	
Cox7a2	1190.38	303.96	0.26	-1.97	1.45E-07	2.10E-05	
Cox7b	1696.32	749.76	0.44	-1.18	8.68E-06	0.000739806	
Cox8a	1783.45	988.42	0.55	-0.85	0.000706031	0.022364515	
Cyp27b1	3.75	233.03	62.20	5.96	5.50E-09	1.27E-06	
Ndufa11	966.85	229.66	0.24	-2.07	1.31E-08	2.62E-06	Complex I
Ndufa13	817.24	334.35	0.41	-1.29	0.00038447	0.014368374	
Ndufa2	602.15	164.36	0.27	-1.87	2.40E-05	0.001645658	
Ndufa5	230.83	20.26	0.09	-3.51	2.27E-05	0.001566944	
Ndufb10	742.86	319.72	0.43	-1.22	0.001246519	0.033123357	
Ndufb11	944.43	369.25	0.39	-1.35	8.00E-05	0.004381781	
Ndufb2	416.34	126.09	0.30	-1.72	0.000918555	0.027274428	
Ndufb3	396.07	126.09	0.32	-1.65	0.001797277	0.043148871	
Ndufb8	813.88	219.52	0.27	-1.89	0.000579175	0.019313885	
Ndufb9	1211.79	431.17	0.36	-1.49	1.97E-06	0.000210659	
Ndufc2	1486.70	614.67	0.41	-1.27	5.86E-06	0.000532564	
Ndufs6	376.69	64.17	0.17	-2.55	1.64E-05	0.001253071	
Ndufv3	828.81	390.64	0.47	-1.09	0.002157213	0.048852832	
Ndor1	209.90	782.40	3.73	1.90	2.66E-06	0.000269397	
Rpl13a	1619.13	636.05	0.39	-1.35	9.07E-07	0.000111832	Ribosomal subunits
Rpl14	1448.69	471.69	0.33	-1.62	4.09E-08	6.95E-06	
Rpl22	1352.84	681.09	0.50	-0.99	0.000488305	0.017393076	
Rpl22l1	742.74	272.43	0.37	-1.45	0.000170912	0.007869345	
Rpl29	977.46	466.06	0.48	-1.07	0.001104889	0.03059486	
Rpl32	3663.74	1316.01	0.36	-1.48	1.61E-12	7.87E-10	
Rpl36al	1651.97	891.60	0.54	-0.89	0.000614025	0.020206063	
Rpl38	301.12	47.28	0.16	-2.67	6.52E-05	0.003690809	
Rpl41	9232.17	3217.42	0.35	-1.52	6.15E-09	1.37E-06	
Rpl8	3049.55	1108.87	0.36	-1.46	4.04E-11	1.57E-08	
Rplp0	1081.18	529.11	0.49	-1.03	0.000975463	0.028353845	
Rplp1	2091.32	552.75	0.26	-1.92	2.97E-13	1.73E-10	
Rps11	1754.68	761.01	0.43	-1.21	4.37E-06	0.000416959	
Rps14	1438.54	333.23	0.23	-2.11	3.75E-05	0.002361914	
Rps15	2313.78	865.71	0.37	-1.42	4.51E-09	1.07E-06	
Rps15a	1076.58	395.14	0.37	-1.45	9.87E-06	0.000831648	
Rps17	704.56	307.33	0.44	-1.20	0.001832148	0.043778235	
Rps20	1126.82	388.39	0.34	-1.54	2.17E-06	0.000225538	

Rps21	1023.31	452.56	0.44	-1.18	0.000289137	0.011636817
Rps24	1994.06	717.11	0.36	-1.48	9.24E-09	1.95E-06
Rps25	1320.93	670.95	0.51	-0.98	0.000635685	0.020609513
Rps27l	936.36	274.69	0.29	-1.77	8.32E-07	0.000104333
Rps28	447.27	119.33	0.27	-1.91	0.000196341	0.008813862
Rps3	3520.68	1400.45	0.40	-1.33	2.01E-10	6.61E-08
Rps4x	2240.30	701.35	0.31	-1.68	2.39E-11	9.56E-09
Rps5	4075.84	1368.92	0.34	-1.57	1.36E-14	9.85E-12
Rps6kb2	137.19	466.06	3.40	1.76	0.000448689	0.016209421
Rps9	1981.66	722.74	0.36	-1.46	1.44E-08	2.84E-06

This table lists a subset of genes differentially expressed between $ALDH^{-}$ and $ALDH^{+}$ cells, arranged by functional category.

Supplementary Table 3 Ingenuity analyses of differentially activated or suppressed functional networks in ALDH^{-} vs. ALDH^{+} cells isolated from wild-type mice

**Transcription Factor ACTIVATION**	**Wild-type ALDH+**	**Foxo KO ALDH+**
CREB1	z= **2.020,** p=5.66E-03	
CTNNB1		z= **2.028,** p=4.66E-02
GLI1	z= **2.779,** p=1.00E00	
IRF4		z= **2.236,** p=2.23E-01
MITF		z= **2.000,** p=4.72E-01
MYOCD	z= **2.200,** p=9.37E-02	
NEUROG3	z= 0.479, p=3.72E-02	
NFKB1	z= **2.178,** p=3.70E-01	z= **2.219,** p=4.03E-01
STAT1		z= **2.207,** p=1.00E-00
STAT3	z= 1.817, p=1.00E00	
STAT4	z= **2.006,** p=1.46E-02	
SPIB	z= **2.000,** p=4.92E-01	
WT1	z= 1.967, p=2.61E-01	
XBP1	z= 0.204, p=1.58E-02	
**Transcription Factor INHIBITION**	**Wild-type ALDH+**	**Foxo KO ALDH+**
ATF4	z= **-**1.772, p=1.59E-01	
Esrra	z= **-3.148**, p=6.84E-04	
NFE2L2	z= **-2.778**, p=2.68E-04	
NRF1	z=**-2.345**, p=3.43E-05	
MYC	z= **-**1.777, p=2.62E-03	
MYCN	z= **-4.932**, p=1.78E-09	
RBPJ	z= **-**1.622, p=2.56E-02	
TP53	z= **-2.428**, p=1.88E-02	z= **-2.008**, p=7.59E-02
**Other ACTIVATION**	**Wild-type ALDH+**	**Foxo KO ALDH+**
RICTOR	z=**8.699**, p=2.47E-48	z= **4.359** p=5.93E-06
CD24	z=**3.308**, p=5.91E-04	
TSC2	z=**2.000**, p=2.90E-02	
INS	z=**2.499**, p=4.12E-03	z= **2.618** p=1.67E-02
MYD88	z=**2.359**, p=1.00E00	z= 1.961 p=0.00E-00
TICAM1	z=**2.155**, p=2.55E-01	
IFRD1	z=**2.219**, p=8.97E-03	

CUL4B	z=**2.236**, p=1.92E-02	
HIST1H1T		z= **2.000**, p=1.38E-01
**Other INHIBITION**	**Wild-type ALDH+**	**Foxo KO ALDH+**
SOCS1		z= -1.969 p=1.51E-01
HIST1H1T	z= 0.632 p=4.19E-03	z= **2.000** p=1.38E-01
**Biological and chemical drug (random cut)**	**Wild-type ALDH+**	**Foxo KO ALDH+**
CD437	z=**4.562**, p=7.15E-13	z= **2.496** p=9.66E-04
sirolimus	z=**3.748**, p=4.48E-11	
5-fluorouracil	z=**3.115**, p=3.14E-09	
**Biological and chemical drug (random cut)**	**Wild-type ALDH+**	**Foxo KO ALDH+**
mono-2-ethyl-phthlate	z**=-2.626**, p=1.98E-07	z= **-2.138** p=9.33E-03
2-amino-1-methyl-6 phenylimidazo(4,5-b)pyridine	z**=-3.000**, p=9.83E-05	
1,2-dithiol-3-thione	z**=-3.393**, p=3.17E-04	
curcumin		z= **-2.143** p=5.42E-01
**Nuclear receptor (NR) ACTIVATION**	**Wild-type ALDH+**	
ESR2 (NR)	z=**2.613**, p=1.00E00	
NR5A2 (NR)	z=**2.433**, p=1.66E-01	
AR (NR)	z=**2.260**, p=3.50E-01	
TLR7 (TM)	z=**2.219**, p=5.35E-01	
ADORA2A (GPCR)		z= 0.412 p=1.03E-03
**Transmembrane receptor (TM), GPCR, Nuclear receptor (NR) INHIBITION**	**Wild-type ALDH+**	**Foxo KO ALDH+**
IGF1R (TM)	z**=-2.399**, p=2.23E-06	z= -**2.496** p=1.43E-03
NR4A3 (NR)	z**=-2.213**, p=2.50E-02	
**Enzyme (EZ), Kinase (KN), Peptidase (PD), Phosphatase (PPT) ACTIVATION**	**Wild-type ALDH+**	**Foxo KO ALDH+**
PSEN1 (PD)	z=**2.064**, p=5.05E-06	
SRC (KN)	z=**2.131**, p=2.43E-01	
PTGS2 (EZ)	z=**2.737**, p=1.02E-01	
NOS2 (EZ)	z=**2.180**, p=1.00E00	
SURF1 (EZ)	z=**2.000**, p=2.10E-02	
EGFR (KN)	z=1.971, p=2.42E-01	z=**2.076**, p=3.21E-01
MET (KN)		z=**2.153**, p=3.44E-02
F2 (PD)		z=**2.205**, p=2.17E-01
**Enzyme (EZ), Kinase (KN), Peptidase (PD), Phosphatase (PPT) INHIBITION**	**Wild-type ALDH+**	**Foxo KO ALDH+**
PRKAA1 (KN)	z**=-2.236**, p=3.47E-01	

SOCS3 (PPT)	z**=-2.236**, p=2.29E-01	
INSR (KN)	z**=-3.637**, p=5.01E-04	
EIF2AK4 (KN)	z**=-2.630**, p=4.28E-04	
**Growth factor ACTIVATION**	**Wild-type ALDH+**	**Foxo KO ALDH+**
HGF	z=**3.30**, p=4.71E-01	
EGF	z=**2.090**, p=1.00E00	z=**2.186**, p=1.00E00
AGT		z=**2.014**, p=1.85E-01
**Cytokine ACTIVATION**	**Wild-type ALDH+**	**Foxo KO ALDH+**
CXCL12	z=**2.773**, p=5.03E-01	
EDN1	z=**2.239**, p=1.31E-01	
IL6	z=**2.310**, p=1.00E00	
OSM	z=**2.294**, p=1.00E00	
LIF		z=**2.364**, p=8.55E-02
WNT3A		z=**2.342**, p=2.342E-01

A partial list of different Ingenuity Pathway analyses to identify trends in gene expression in the different islet cell types.

Supplementary Table 4 List of differentially expressed transcripts in $ALDH^+$ cells from wild-type and Foxo knockout mice

Gene	baseMean_all	baseMean_WT ALDH+	baseMean_Rip-Foxo KO ALDH+	Fold-Change	log2 Fold-Change	p-value	Adjusted p-value
Foxo1	349.7794248	1005.012999	131.3682334	0.130712969	-2.935525802	2.23E-11	3.47E-07
Cyb5r3	2576.053292	5076.014959	1742.732737	0.343326951	-1.542344984	8.29E-09	6.46E-05
Cyp27b1	55.32317138	206.7969094	4.831925389	0.023365559	-5.419472636	5.10E-07	0.002649712
Elovl7	124.8599548	384.622271	38.27251604	0.09950676	-3.32906165	1.46E-06	0.005684329
Hip1r	716.4685825	1463.562668	467.4372208	0.319383127	-1.646639999	2.98E-06	0.009288892
Bach2	61.74301035	211.7920038	11.7266792	0.055368848	-4.174781692	2.45E-05	0.052959279
Ptprt	324.3808944	753.2602399	181.4211126	0.240847854	-2.053806023	2.71E-05	0.052959279
Etl4	836.462433	1573.454745	590.7983289	0.375478437	-1.413198035	3.21E-05	0.052959279
Muc4	1555.856509	3932.138334	763.7625667	0.194235935	-2.364117959	3.32E-05	0.052959279
Ctsl	4496.474551	2272.767965	5237.71008	2.304551173	1.204485803	3.39E-05	0.052959279
Dnahc17	29.21450191	112.8891341	1.322957853	0.011719089	-6.414995718	3.87E-05	0.054903167
Spp1	2405.635018	3933.137353	1896.467572	0.482176797	-1.052365866	0.000104638	0.136037989
Gpc6	18.23209467	72.92837866	0	0	#NAME?	0.000119737	0.14369393
Cxcl13	17.98233994	71.92935978	0	0	#NAME?	0.000135081	0.150528447
Prnd	24.27000451	93.90777526	1.057414255	0.011260135	-6.472632027	0.000149671	0.155667934
Ncam1	1479.626588	2471.572723	1148.977876	0.464877228	-1.105078338	0.000183578	0.173360099
2010015L04Rik	87.00878742	249.7547214	32.76014275	0.131169263	-2.930498403	0.000188906	0.173360099
Jam2	182.4268457	437.570272	97.37903698	0.22254491	-2.167831588	0.000207049	0.179454216
Galntl4	119.5275192	316.6889868	53.80702993	0.169904961	-2.557200117	0.000328609	0.269822705
Nog	15.48479273	61.93917092	0	0	#NAME?	0.000451442	0.351638321
D0H4S114	296.5293792	613.3975959	190.9066403	0.311228217	-1.683955227	0.000492415	0.351638321
Hcn1	19.67871965	76.92445421	0.596808134	0.007758367	-7.010031294	0.000495868	0.351638321
Cox6b1	579.2995025	229.7743437	695.8078888	3.028222723	1.598471318	0.000553818	0.362501015
Krba1	187.7400796	424.5830265	108.7924307	0.256233584	-1.964468514	0.000565259	0.362501015
Zfp618	95.90087591	259.7449103	41.28619777	0.158949015	-2.653364014	0.000580894	0.362501015
Pygo1	28.58340272	104.896983	3.145542627	0.029986969	-5.059520461	0.000612413	0.367471323
Anpep	1218.657663	1993.042677	960.5293244	0.481941172	-1.053071039	0.000641507	0.370672475
Arhgef6	37.83659814	130.871474	6.824972837	0.052150195	-4.261183554	0.000673245	0.375117954
D330022K07Rik	201.7932795	449.5584986	119.2048731	0.26515987	-1.915065643	0.000707598	0.380663317
Eml5	4561.421402	6798.323518	3815.787363	0.56128358	-0.83319824	0.000836787	0.404802152
5730508B09Rik	59.66791457	180.8224183	19.28307998	0.10664098	-3.229166145	0.000839858	0.404802152
Lair1	26.73296816	97.90385081	3.009340615	0.030737715	-5.023846292	0.000840268	0.404802152
Gpr98	418.6612241	780.2337498	298.1370488	0.382112474	-1.387930738	0.00087865	0.404802152
Slc2a4rg-ps	109.1071855	278.7262691	52.56749097	0.188598983	-2.406606196	0.000882205	0.404802152
Cd44	839.3634842	1552.475349	601.6595295	0.387548524	-1.367551138	0.000984629	0.438891384
Tspan2	697.327608	1209.811871	526.4995204	0.435191234	-1.200278599	0.001036288	0.449086763
Abcc10	216.7436511	460.5477064	135.4756327	0.294161996	-1.765317222	0.001092283	0.460559503
Rfx6	1632.655799	2557.488348	1324.378282	0.51784333	-0.949412409	0.001140404	0.46767398

Ckb	1406.377612	743.270051	1627.413466	2.189531872	1.13062245	0.00116911	0.46767398
Dtx4	18.65374909	71.92935978	0.895212201	0.012445713	-6.328207254	0.001389083	0.541776915
Fat1	769.095564	1287.735344	596.2156374	0.462995475	-1.110930001	0.001469204	0.55904989
Hapln4	886.6905817	1460.565611	695.3989052	0.47611617	-1.070614467	0.001546453	0.560290004
Uchl1	54.26867086	161.8410595	18.41120798	0.113761045	-3.135921478	0.00156609	0.560290004
Park7	683.5129877	315.6899679	806.120661	2.553520045	1.352487384	0.001638868	0.560290004
Szt2	354.4530757	666.3455968	250.488902	0.375914395	-1.411523932	0.001650792	0.560290004
Nnt	632.9867799	285.7194013	748.7425727	2.620552084	1.389870783	0.001652031	0.560290004
Rsph4a	105.4461812	260.7439292	53.68026525	0.2058735	-2.280169958	0.001811435	0.586371216
Insrr	2490.680039	3749.317878	2071.134093	0.552402906	-0.856207185	0.001840231	0.586371216
Adora3	701.0062234	328.6772134	825.1158934	2.510414047	1.32792533	0.001841689	0.586371216
Spnb3	409.8191874	745.2680888	298.0028869	0.399859985	-1.32243318	0.002037133	0.617919778
BC021891	122.4157883	291.7135147	65.98321278	0.226191827	-2.144381295	0.002050793	0.617919778
Mansc1	159.7771067	357.6487611	93.81988857	0.262324098	-1.930577752	0.002059601	0.617919778
Cenpt	40.15175484	127.8744174	10.91086732	0.085324864	-3.550889973	0.002145816	0.624257214
Dock5	818.1777035	1343.680401	643.0101375	0.478543958	-1.063276641	0.002160752	0.624257214
Reln	183.3020628	392.6144221	113.5312764	0.289167361	-1.790023373	0.002232299	0.633201798
Zc3hav1l	404.6971005	733.2798622	295.1695133	0.402533233	-1.3128202	0.002353068	0.655063124
Cdc14a	37.8092999	121.8803041	9.78563184	0.08028887	-3.638656188	0.002393346	0.655063124
Hscb	174.7209022	39.96075543	219.6409512	5.496416391	2.458491302	0.002469258	0.664187958
Mgat4b	102.9269687	12.98724552	132.9068765	10.23364626	3.355248365	0.002741143	0.674279216
Zcchc12	103.516591	253.750797	53.43852234	0.2105945	-2.247460333	0.00275132	0.674279216
Rnf150	441.1221262	1061.957076	234.1771431	0.220514697	-2.18105328	0.00275409	0.674279216
Senp7	553.1422547	946.0708848	422.1660446	0.446230881	-1.164137737	0.00275645	0.674279216
Zfp9	1258.235545	1947.087808	1028.618124	0.528285432	-0.920610468	0.002778284	0.674279216
Acp1	99.30841826	241.7625704	51.8237009	0.214357834	-2.221906952	0.002798093	0.674279216
Pik3c2b	51.65012096	153.8489084	17.58385848	0.114293034	-3.129190622	0.002916368	0.674279216
Gabarap	2085.90723	1232.789305	2370.279871	1.922696653	0.943131165	0.002949357	0.674279216
Map3k14	114.3877259	269.7350992	62.60526811	0.232099079	-2.107187297	0.003014706	0.674279216
Gm7694	30.29055789	102.8989452	6.087762111	0.059162532	-4.079172399	0.003016649	0.674279216
Shfm1	184.1675899	46.95388763	229.9054907	4.896410123	2.291724403	0.003036205	0.674279216
0610011L14Rik	300.2149029	104.896983	365.3208762	3.482663331	1.800191014	0.003043961	0.674279216
Syp	3752.589483	5394.701983	3205.21865	0.59414193	-0.751120487	0.003095261	0.674279216
Asb4	52.43751126	153.8489084	18.63371221	0.121116961	-3.045527186	0.003179074	0.674279216
Rcbtb2	247.1147663	488.5202352	166.6462767	0.341124614	-1.551629237	0.003190525	0.674279216
Abca8b	103.2835729	249.7547214	54.45985672	0.218053362	-2.19724686	0.003198299	0.674279216
Sema5a	27.99619274	95.90581304	5.359652642	0.055884544	-4.161406854	0.003271185	0.680450126
Ampd3	18.65008631	69.93132201	1.556341079	0.022255279	-5.489708605	0.00338768	0.690269968
Mical3	188.2327279	388.6183466	121.4375217	0.312485303	-1.678139758	0.003406883	0.690269968
Odz4	476.6943391	816.1984297	363.5263089	0.445389621	-1.166860157	0.003464682	0.692980829
Csn3	52.78916132	153.8489084	19.10257895	0.12416454	-3.009674879	0.003573554	0.705709148
Acsm3	20.419877	74.92641643	2.251030523	0.030043216	-5.056816928	0.003634916	0.70885403
Gm3086	25.42427708	87.91366195	4.594482123	0.052261298	-4.258113221	0.003720521	0.71659077

Fermt1	25.17729849	87.91366195	4.265177338	0.048515524	-4.365409748	0.003871923	0.727248507
Mapk15	176.4894495	369.6369877	112.1069367	0.303289282	-1.721233578	0.003882527	0.727248507
Rcn2	824.8674612	417.5898943	960.6266501	2.300406843	1.201889034	0.003915702	0.727248507
9130014G24Rik	147.2601177	317.6880057	90.45082167	0.284715885	-1.812405108	0.003965223	0.727781765
Acin1	768.9366229	1229.792248	615.318081	0.500343112	-0.999010327	0.004137312	0.749799363
Lrrc4	181.9183183	376.6301199	117.0143844	0.310687803	-1.686462491	0.004181305	0.749799363
Ccdc9	133.6720912	292.7125335	80.65861048	0.275555712	-1.859584064	0.004240864	0.751837728
Pappa2	1472.075132	3048.006621	946.7646354	0.310617644	-1.686788313	0.004443404	0.778893754
Gpr116	106.85621	254.7498159	57.55834141	0.22594066	-2.145984178	0.004499145	0.779901746
Kcnh6	865.9945329	1455.570517	669.469205	0.459935948	-1.120495134	0.004563555	0.782373896
Wscd1	35.48836532	111.8901152	10.02111536	0.089562115	-3.480967593	0.004787026	0.786649589
Scly	1152.834569	1749.282069	954.018736	0.545377302	-0.874673436	0.004797405	0.786649589
Ifi27l1	965.323022	528.4809906	1110.937032	2.102132437	1.071853563	0.004848354	0.786649589
Osbpl6	484.7301871	825.1895997	371.2437163	0.449888991	-1.152359031	0.004894949	0.786649589
Akap12	40.55993293	0	54.07991057	Inf	Inf	0.004985366	0.786649589
Zfp41	40.63394807	124.8773607	12.55281052	0.100521107	-3.314429632	0.004990415	0.786649589
Atox1	305.017597	116.8852096	367.7283929	3.146064365	1.653548187	0.004999643	0.786649589
Pkib	219.5890818	434.5732153	147.9277039	0.340397656	-1.554706991	0.005030792	0.786649589
Slurp1	10.4896983	41.9587932	0	0	#NAME?	0.005042302	0.786649589
Slc7a15	98.88376209	230.7733626	54.92056191	0.237984841	-2.071058417	0.005158246	0.796770209
Adcy9	659.3747747	1059.959038	525.846687	0.496100951	-1.011294371	0.005378564	0.822656606
Rbm11	20.02251907	72.92837866	2.387232536	0.032733931	-4.933069312	0.005433998	0.823066024
Rhob	156.6510851	329.6762323	98.97603606	0.300221934	-1.735898712	0.005527738	0.829213818
3930402G23Rik	19.04945185	69.93132201	2.088828469	0.029869712	-5.065172848	0.005639765	0.837079344
Kctd19	10.23994358	40.95977432	0	0	#NAME?	0.005687482	0.837079344
Syce1	12.21202968	47.95290652	0.298404067	0.006222857	-7.328207254	0.005802659	0.838903494
Rps19bp1	80.55654805	8.991169972	104.4116741	11.6126905	3.537630358	0.005807421	0.838903494
Clip3	717.6030719	1135.884473	578.1759382	0.509009456	-0.974235636	0.0059695	0.854405229
Mios	292.2013619	537.4721606	210.444429	0.391544799	-1.352750711	0.006025654	0.854602107
Srek1	1569.599895	2303.737551	1324.887343	0.575103419	-0.79810668	0.006215742	0.861142883
4931430N09Rik	92.53208695	216.7870982	51.11374987	0.23577856	-2.084495558	0.006327141	0.861142883
Khdrbs2	14.77932509	55.9450576	1.057414255	0.018900941	-5.725398097	0.006343136	0.861142883
Znf512b	676.9945567	1075.94334	544.011629	0.50561364	-0.98389271	0.0063632	0.861142883
Nubp1	108.9000387	249.7547214	61.94847775	0.248037264	-2.011371215	0.006370092	0.861142883
Syt17	9.990188858	39.96075543	0	0	#NAME?	0.00641488	0.861142883
Tnfrsf9	170.8533096	26.97350992	218.8132428	8.112153128	3.020084885	0.006458158	0.861142883
Dbpht2	645.9488111	1044.973755	512.9404966	0.490864478	-1.026603327	0.006669144	0.88174001
Lpcat4	234.4182031	443.5643853	164.7028091	0.371316577	-1.429278372	0.006750219	0.884959356
Mir665	32.38846859	100.9009075	9.550988961	0.094657117	-3.401145214	0.006916038	0.896346774
Cldn2	43.89976497	127.8744174	15.90821417	0.124404979	-3.006883871	0.007076861	0.896346774
Zfp760	221.2580919	423.5840076	153.81612	0.36313014	-1.461441415	0.007149886	0.896346774
2310069G16Rik	11.73664052	45.95486875	0.330564439	0.007193241	-7.119142374	0.007152067	0.896346774
BC060267	11.71252024	45.95486875	0.298404067	0.006493416	-7.266806709	0.0072036	0.896346774

Gm5577	79.00028063	191.8116261	41.39649881	0.215818507	-2.212109508	0.007208116	0.896346774
Fosl2	396.6341474	175.8273239	470.2364219	2.674421765	1.419227001	0.00723926	0.896346774
Pclo	14878.82559	20227.13538	13096.05566	0.647449844	-0.627159658	0.007401541	0.909019303
Golgb1	8026.93756	10974.22246	7044.50926	0.641914203	-0.639547613	0.007458142	0.909019303
Mll3	5224.039285	7185.942846	4570.071432	0.635973808	-0.652960745	0.007897916	0.929055999
Sv2b	225.7449488	431.5761587	157.1345455	0.364094592	-1.457614781	0.007928029	0.929055999
Gm15421	768.1652633	417.5898943	885.0237197	2.119360961	1.083629323	0.007988908	0.929055999
Fubp3	1061.260638	615.3956336	1209.882306	1.966023546	0.9752806	0.008003981	0.929055999
Atf4	5748.836747	3668.397349	6442.316547	1.756166504	0.812429635	0.008029915	0.929055999
Peg3	16357.60053	23456.96344	13991.14623	0.596460248	-0.745502104	0.008035593	0.929055999
Glipr2	15.22771244	56.94407649	1.322257757	0.023220286	-5.428470432	0.008039392	0.929055999
Pnn	1589.388383	2308.732645	1349.606963	0.584566154	-0.774561795	0.008102446	0.929457757
F8	232.2122903	435.5722342	164.4256423	0.377493397	-1.405476685	0.008359615	0.951958836
Mllt11	607.8142128	315.6899679	705.1889611	2.233802252	1.159501476	0.008520225	0.957282449
Pak6	44.03293285	0.999018886	58.37757083	58.43490215	5.868758418	0.008529085	0.957282449
1700029J07Rik	132.3054307	298.7066469	76.83835866	0.257236856	-1.958830734	0.008667624	0.965882827
E030024N20Rik	103.1366902	227.776306	61.59015165	0.270397535	-1.886846095	0.008858574	0.974747057
Slc6a17	2184.263117	1386.638213	2450.138085	1.76696276	0.821271635	0.008915421	0.974747057
Hmgn2	56.97685044	148.853814	26.35119593	0.177027348	-2.497955843	0.008934609	0.974747057
Akr1c12	746.2705201	411.5957809	857.8287665	2.084153449	1.059461502	0.009132903	0.979040782
Carhsp1	297.093596	121.8803041	355.4980267	2.916779946	1.544376547	0.009172763	0.979040782
Mst1	26.09401833	85.91562418	6.153483049	0.071622398	-3.803445363	0.009197497	0.979040782
Ephx1	102.9091246	229.7743437	60.62071828	0.263827185	-1.922334866	0.009298081	0.979040782
Spata17	19.9620638	69.93132201	3.305644392	0.047269868	-4.40293534	0.009341102	0.979040782
Smad9	15.8239719	57.94309538	1.784264072	0.030793385	-5.021235701	0.009366325	0.979040782
Cd300lf	14.8267844	54.94603872	1.453699632	0.02645686	-5.240214364	0.009416237	0.979040782
Gpx1	823.1807359	280.7243069	1003.999546	3.576461036	1.838532724	0.009476005	0.979040782
St8sia3	759.3557811	421.5859698	871.9457183	2.068251272	1.048411469	0.009583968	0.983680829
Mef2c	91.95814881	14.98528329	117.6157707	7.848751899	2.972463256	0.009739732	0.99313438
Gtpbp3	64.26162857	5.994113315	83.68413366	13.961053	3.803335854	0.009876353	0.998148762
Gipr	259.1740891	468.5398574	189.3854996	0.404203605	-1.306845905	0.009950358	0.998148762
Mterfd2	80.0678122	189.8135883	43.48588684	0.22909786	-2.125964114	0.009980848	0.998148762
Dscr3	258.4056264	467.5408386	188.693889	0.403588037	-1.309044685	0.010359423	1
Rsad1	71.46109951	176.8263428	36.33935175	0.2055087	-2.282728628	0.010417955	1
Spin2	43.45642461	120.8812852	17.64813775	0.145995616	-2.776003042	0.010460312	1
A030009H04Rik	139.1546292	287.7174391	89.63369253	0.311533749	-1.682539632	0.010546558	1
Ttll10	114.9002011	243.7606081	71.94673212	0.295153235	-1.760463939	0.010556016	1
Zfp839	176.4527049	341.6644589	121.3821202	0.355267038	-1.493024256	0.01056986	1
Unc119b	236.6692258	88.91268084	285.9214074	3.215755107	1.685157543	0.010806077	1
Fis1	258.3436797	76.92445421	318.8167549	4.144543607	2.051213242	0.010858969	1
Serinc2	240.9717851	73.92739755	296.6532476	4.012764651	2.004596544	0.010892107	1
Mtap6	184.4186426	355.6507233	127.3412823	0.358051521	-1.4817609	0.010939889	1
Ush1g	44.14456331	123.8783418	17.56663714	0.141805556	-2.818014036	0.011079634	1

Ppig	1507.947172	2175.863133	1285.308518	0.590712025	-0.759473113	0.011183814	1
Rala	319.743029	139.862644	379.7031573	2.714828967	1.440861312	0.011239188	1
Ush2a	804.4036655	1224.797154	664.2725026	0.542353075	-0.882695735	0.01138955	1
4933411K20Rik	989.5923819	1473.552857	828.2722237	0.562091967	-0.831121898	0.011434747	1
Gpm6b	14.22869355	52.94800095	1.322257757	0.024972761	-5.323500872	0.01151728	1
Arc	111.847008	239.7645326	69.20783318	0.288649169	-1.792611021	0.011612183	1
Sema6d	293.8280289	515.4937451	219.9394569	0.426657858	-1.228848477	0.01164791	1
Dusp26	11.98456385	45.95486875	0.661128878	0.014386482	-6.119142374	0.011654718	1
Krtcap2	783.3223359	444.5634042	896.2419798	2.016004852	1.011499111	0.011717538	1
2610037D02Rik	30.80442562	95.90581304	9.103963153	0.094926083	-3.397051639	0.011760204	1
Ift46	186.0249008	60.94015203	227.7198171	3.736777962	1.901794842	0.011839368	1
Bcar1	505.6767748	257.7468725	588.3200755	2.282549812	1.190646345	0.011896315	1
Aaas	168.5382744	325.6801568	116.157647	0.356661726	-1.487371687	0.011915945	1
Sepw1	372.2169467	171.8312484	439.0121795	2.554903044	1.353268543	0.012082112	1
Eif6	465.2566994	232.7714004	542.751799	2.331694521	1.221378791	0.012166414	1
Fam43a	79.91294912	187.8155505	43.94541532	0.233981772	-2.095531952	0.012262902	1
Fam129a	127.2652859	263.7409858	81.77338598	0.31005187	-1.689418502	0.012263041	1
Cox6a2	575.4274722	302.7027224	666.3357221	2.20128751	1.138347588	0.012286981	1
BC016495	45.85322289	124.8773607	19.51184361	0.156248046	-2.678089946	0.012291213	1
Grem2	405.7759667	193.8096638	476.431401	2.458243782	1.297627993	0.012414536	1
Gpr158	2694.466721	3770.297275	2335.856536	0.61954174	-0.69072661	0.012461845	1
Fnbp4	976.3796444	1470.5558	811.6542593	0.55193707	-0.85742431	0.012475549	1
Ubl5	475.8358909	241.7625704	553.8603311	2.290926715	1.195931309	0.012564173	1
Slc23a2	1325.685089	758.2553343	1514.828341	1.997781318	0.998398671	0.012588027	1
Ndor1	429.8267035	694.3181256	341.6628962	0.49208408	-1.023023252	0.012807815	1
2210019I11Rik	170.6620464	53.94701983	209.5670553	3.884682712	1.957796769	0.012886208	1
2900010M23Rik	478.4758487	207.7959282	568.7024888	2.736831725	1.452506733	0.01313826	1
Wfikkn2	8.491660529	33.96664212	0	0	#NAME?	0.01318776	1
BC052040	190.6770446	358.64778	134.6867994	0.375540592	-1.41295924	0.013195464	1
Ptpmt1	114.7723648	26.97350992	144.0386497	5.340003958	2.416840811	0.013204016	1
Iqsec2	104.9185068	228.7753248	63.63290076	0.278145822	-1.84608666	0.013511336	1
2810454H06Rik	34.53331918	101.8999264	12.07778345	0.118525929	-3.076725392	0.013743684	1
Gm5506	84.67478541	13.9862644	108.2376257	7.738851679	2.952119509	0.013807261	1
Dom3z	143.9441357	289.7154769	95.35368862	0.329128736	-1.603276104	0.013949348	1
Txlna	664.0022971	367.63895	762.7900795	2.074834779	1.052996458	0.014025151	1
Ndufa10	511.1454786	262.741967	593.9466492	2.260570156	1.176686692	0.014052469	1
Pak3	1453.175941	2088.94849	1241.251758	0.594199313	-0.750981157	0.014133127	1
Il6ra	1311.573815	1883.1506	1121.04822	0.595304603	-0.748300046	0.014234892	1
Arl6ip4	248.3668594	97.90385081	298.5211956	3.04912619	1.608395858	0.01423705	1
Odf2	604.7560908	940.0767715	492.9825306	0.524406671	-0.931242056	0.014665467	1
Cd93	39.77321847	0.999018886	52.69795167	52.74970516	5.721091125	0.014691258	1
Capg	8.241905808	32.96762323	0	0	#NAME?	0.014866189	1
Ptprk	42.49711273	117.8842285	17.36807413	0.147331618	-2.762861022	0.014894166	1

Spin4	52.11335849	132.8695118	25.19464071	0.189619427	-2.398821314	0.015285803	1
Ndufa5	113.7351011	17.98233994	145.6526881	8.099762799	3.017879659	0.015306297	1
Nkrf	234.2204936	92.90875638	281.324406	3.027964392	1.59834824	0.015617042	1
Mtap1a	82.48288773	187.8155505	47.37200013	0.252226187	-1.987210027	0.015769673	1
Cep290	947.7852289	1400.624478	796.8388126	0.568916812	-0.813710381	0.015778122	1
Banp	272.0150785	473.5349519	204.8417873	0.43258008	-1.208960863	0.016068764	1
Hibch	206.3062244	381.6252144	147.8665611	0.387465386	-1.36786066	0.016076633	1
2810407C02Rik	4228.601729	2910.142014	4668.0883	1.604075773	0.681742293	0.016268856	1
Dock3	153.7289053	46.95388763	189.3205779	4.032053306	2.011514712	0.016461182	1
Gpr119	94.23999086	225.7782682	50.39389842	0.223200837	-2.163585658	0.016539453	1
Pla2g16	110.447192	229.7743437	70.67147471	0.307569042	-1.701017799	0.016629251	1
Cnn3	422.1118975	210.7929849	492.551535	2.336659995	1.224447824	0.016638581	1
9330162B11Rik	9.763248096	37.96271766	0.363424908	0.009573206	-6.706782164	0.016728787	1
Fgfbp1	7.992151086	31.96860435	0	0	#NAME?	0.016756337	1
Pdgfrb	7.992151086	31.96860435	0	0	#NAME?	0.016756337	1
Zbtb34	361.2109228	596.4142748	282.8098055	0.474183495	-1.076482646	0.016821389	1
Syne1	1014.715628	1474.551875	861.4368793	0.584202491	-0.775459584	0.016840019	1
D4Ertd22e	322.8578862	146.8557762	381.5252563	2.597958801	1.377378552	0.016931136	1
Epdr1	39.62283772	109.8920774	16.19975781	0.147415157	-2.76204323	0.016941233	1
Rfxank	60.27130093	148.853814	30.74379657	0.206536841	-2.275528947	0.016955392	1
D930020B18Rik	9.714482465	37.96271766	0.298404067	0.007860451	-6.991172267	0.016966585	1
Aasdh	131.4892047	261.7429481	88.07129019	0.336480088	-1.571406963	0.017045033	1
Baiap2	82.92154062	188.8145694	47.62386435	0.25222558	-1.987213496	0.017220534	1
Iqgap2	1283.687248	809.2052975	1441.847898	1.78180729	0.833341312	0.017360371	1
Glce	1752.844627	1143.876624	1955.833962	1.70982947	0.773852445	0.01739395	1
Bmyc	334.4996982	559.450576	259.5160723	0.463876674	-1.108186794	0.017396077	1
Copz2	557.0892452	867.1483929	453.736196	0.523250922	-0.934425147	0.017820698	1
Ube3c	1279.887696	808.2062786	1437.114835	1.778153515	0.830379883	0.017940742	1
Dad1	1659.430627	1076.942359	1853.593383	1.721163039	0.783383764	0.01797145	1
Dusp10	553.8401637	254.7498159	653.5369463	2.565406943	1.359187694	0.017972515	1
Snx32	181.9506436	335.6703456	130.7107429	0.389402116	-1.360667371	0.018027568	1
Marcksl1-ps4	91.3628106	17.98233994	115.8229675	6.440928591	2.687268697	0.018069794	1
Rnaseh2a	54.90515919	4.995094429	71.54184745	14.32242142	3.840203517	0.018086716	1
Memo1	418.0341871	211.7920038	486.7815815	2.298394523	1.20062646	0.018152736	1
Tapbp	1501.738693	976.0414514	1676.971107	1.718135131	0.780843509	0.018667325	1
1500032L24Rik	528.5438465	288.716458	608.4863094	2.107556714	1.075571454	0.018712234	1
Tal2	7.742396365	30.96958546	0	0	#NAME?	0.018884459	1
Adora2a	41.43438415	0	55.24584553	Inf	Inf	0.018964243	1
Bmpr1b	47.48659278	123.8783418	22.02267642	0.177776648	-2.491862261	0.018975736	1
Bcl9	908.3311787	1327.696099	768.5428719	0.578854508	-0.788727316	0.018978064	1
Pygb	69.71760726	162.8400784	38.67678356	0.237513909	-2.073916094	0.019118353	1
Znhit6	230.5982163	408.5987243	171.2647136	0.419151366	-1.254456763	0.019301745	1
5930403L14Rik	14.5240762	51.94898206	2.049107573	0.039444615	-4.66402785	0.019336696	1

Smap2	1861.735094	1227.794211	2073.048722	1.688433375	0.755685252	0.019465447	1
Itgb2l	26.79894609	80.92052975	8.758418207	0.108234811	-3.207763521	0.019515285	1
Vipr2	44.55664112	117.8842285	20.11411198	0.17062598	-2.551090765	0.019581177	1
Ret	820.0320619	1206.814814	691.1044779	0.572668209	-0.804228579	0.019785268	1
Cox8a	1367.305127	877.1385817	1530.693976	1.745099358	0.803309179	0.019916456	1
Ndufaf4	250.6262618	106.8950208	298.5366754	2.792802445	1.481713525	0.01995474	1
6430550D23Rik	26.55144243	80.92052975	8.428413325	0.104156675	-3.263172795	0.020009078	1
Ccdc14	30.50015492	90.91071861	10.36330036	0.113994263	-3.132966876	0.02006723	1
Zfp398	426.7795099	676.3357857	343.5940846	0.508022926	-0.977034492	0.020073849	1
Map4k3	685.0945005	1018.999264	573.7929128	0.563094531	-0.828550955	0.020333643	1
Alox8	66.75013396	153.8489084	37.71720914	0.24515747	-2.028219374	0.020444607	1
Ssx2ip	347.0500948	167.8351728	406.7884022	2.4237375	1.277233458	0.020455457	1
Mysm1	1401.573355	1975.060337	1210.411028	0.61284762	-0.706399691	0.020733276	1
Sdhd	975.6134685	589.4211426	1104.344244	1.873608128	0.90581924	0.021091553	1
B3galt5	266.8893354	456.5516308	203.6685703	0.446101945	-1.164554658	0.021099703	1
Elovl2	788.391855	469.5388763	894.6761812	1.905435793	0.930120995	0.021135918	1
Gm15441	7.492641643	29.97056657	0	0	#NAME?	0.02127996	1
Cth	75.89145944	2.997056657	100.1895937	33.42932923	5.063042502	0.021294003	1
Dcx	1426.898542	924.0924694	1594.500566	1.725477286	0.786995483	0.021627857	1
Slbp	156.4603153	296.7086091	109.710884	0.369759692	-1.43534013	0.021715085	1
Gdap10	185.2931442	337.6683834	134.5013977	0.398323931	-1.327985938	0.021958042	1
Tfb1m	72.62239557	162.8400784	42.54983463	0.261298294	-1.936230391	0.022036294	1
1110059E24Rik	418.1061239	209.793966	487.5435099	2.323915788	1.216557791	0.022037973	1
Plekhb1	107.348896	227.776306	67.20642604	0.295054509	-1.760946591	0.022066734	1
2310061I04Rik	261.1635838	114.8871719	309.9223878	2.697623962	1.431689257	0.022137274	1
Zfp579	66.13002312	151.8508706	37.55640728	0.24732428	-2.015524217	0.022141795	1
B3gnt3	247.637691	425.5820453	188.3229062	0.442506699	-1.176228798	0.022143372	1
Immp1l	520.4829057	288.716458	597.7383883	2.070330152	1.04986085	0.022144122	1
Ptpn3	126.2489432	250.7537403	84.74734422	0.337970409	-1.565031157	0.022226921	1
C130083M11Rik	63.14303411	151.8508706	33.57375527	0.22109689	-2.177249362	0.022231796	1
Setd2	1946.923343	2674.373557	1704.439939	0.63732306	-0.649903233	0.022252345	1
Myadm	890.5377794	1288.734363	757.805585	0.588023108	-0.766055245	0.022304872	1
Ccdc15	126.8734966	252.7517781	84.91406941	0.335958346	-1.573645723	0.022370481	1
Solh	150.3518724	285.7194013	105.2293628	0.368296175	-1.441061679	0.022444392	1
Slc4a10	1431.823019	2000.035809	1242.418756	0.621198255	-0.686874317	0.022526013	1
Lamtor2	157.2458949	53.94701983	191.6788533	3.553094386	1.829076013	0.022616951	1
Tmem14c	224.1378358	92.90875638	267.8808623	2.883268195	1.527705039	0.022711628	1
Tmem59l	84.48357577	181.8214372	52.03762196	0.286201796	-1.804895367	0.02297123	1
Fastkd1	138.5876809	42.95781209	170.4643038	3.968179372	1.988477241	0.023069975	1
Bmf	75.63764245	170.8322295	43.90611344	0.257013056	-1.960086444	0.023102674	1
Sox5	72.00388398	166.8361539	40.39312733	0.242112554	-2.046250207	0.023103723	1
Tomm7	163.8590756	51.94898206	201.16244	3.872307638	1.953193573	0.023156681	1
Eral1	155.2795384	292.7125335	109.4685401	0.373979681	-1.418968205	0.02319876	1

Pitrm1	801.5865022	1173.847191	677.4996059	0.577161671	-0.792952601	0.023217157	1
Ndufa7	320.6824502	154.8479273	375.9606245	2.427934497	1.2797295	0.023217328	1
Eif4e3	434.0089226	680.3318612	351.9012764	0.517249443	-0.951067908	0.023217663	1
Lrrcc1	406.9579795	646.3652191	327.1555663	0.506146613	-0.982372752	0.023226367	1
Ap1m2	213.9275801	86.91464306	256.2652257	2.948470093	1.559966561	0.023230587	1
Ttc14	1455.658675	2026.0103	1265.541467	0.624647104	-0.678886729	0.02327008	1
Dagla	832.5529908	1205.815795	708.1320561	0.587263875	-0.767919201	0.023343629	1
Dennd2d	271.998636	458.5496686	209.8149585	0.45756212	-1.127960473	0.023475251	1
Ssfa2	698.0331979	1030.98749	587.0484338	0.569404032	-0.812475386	0.023502307	1
C85492	121.5672368	243.7606081	80.83611299	0.331620903	-1.592393148	0.023506791	1
Prr22	32.22595806	90.91071861	12.66437122	0.139305589	-2.843674953	0.023549884	1
Plac8	7.242886922	28.97154769	0	0	#NAME?	0.02397574	1
Pcdhac2	29.79267968	86.91464306	10.75202521	0.123707868	-3.014990831	0.02420802	1
Ica1l	339.6830231	553.4564627	268.4252099	0.484997878	-1.04394966	0.024415935	1
Kcnab1	37.79784248	101.8999264	16.43048119	0.161241345	-2.632706371	0.024555687	1
Pfdn4	111.4133587	29.97056657	138.5609561	4.623234458	2.208902528	0.024566917	1
Rtkn2	299.1525382	141.8606818	351.583157	2.478369289	1.309391172	0.024646379	1
Herpud1	2138.228711	1459.566592	2364.449417	1.619966797	0.695964244	0.024736279	1
Zfyve16	566.5830139	861.1542796	468.392592	0.543912517	-0.878553467	0.024737945	1
Ift172	546.1697799	829.1856752	451.8311482	0.544909496	-0.875911463	0.024775454	1
Cep192	490.5600442	753.2602399	402.9933123	0.534998784	-0.902392483	0.025040339	1
A630089N07Rik	40.00943649	107.8940397	17.38123543	0.161095418	-2.634012634	0.025077324	1
Dock1	36.99080138	100.9009075	15.68743268	0.155473653	-2.685257976	0.025156386	1
Parp3	28.02734574	82.91856752	9.730271812	0.117347322	-3.091143181	0.025293984	1
Gm2447	17.26274624	57.94309538	3.702629866	0.06390114	-3.968014514	0.025321564	1
Pfdn2	149.1519432	49.95094429	182.2189428	3.64795792	1.867089088	0.025427446	1
Gca	572.4603531	881.1346573	469.5689184	0.532913913	-0.908025595	0.025460495	1
Aff2	1624.552151	2394.648269	1367.853445	0.571212676	-0.8079001	0.025638213	1
Dcp1a	727.8656801	1063.955113	615.835869	0.578817528	-0.788819483	0.02570339	1
Mettl2	45.67435005	116.8852096	21.93739685	0.187683257	-2.413628139	0.025791204	1
Ggt7	241.5319897	412.5947998	184.511053	0.447196749	-1.161018396	0.025850659	1
Synj2	155.6621914	301.7037035	106.9816873	0.354591893	-1.495768543	0.025893939	1
Ovol2	96.96239052	22.97743437	121.6240426	5.293195079	2.404138825	0.02592148	1
Msln	22.04430074	68.93230312	6.414966607	0.093061835	-3.425666558	0.026166599	1
Dstyk	267.1632343	450.5575175	206.0318066	0.45728192	-1.128844216	0.026268885	1
Itsn2	858.7273155	1234.787343	733.373973	0.593927349	-0.751641628	0.026298917	1
Pcdhb19	41.82605036	107.8940397	19.80338725	0.183544775	-2.445796048	0.026480725	1
Wrnip1	262.8043306	453.5545742	199.2209161	0.439243539	-1.186907031	0.026562434	1
Zfp608	548.6504977	827.1876374	455.8047845	0.551029493	-0.859798556	0.026615913	1
Sowahb	170.238797	62.93818981	206.005666	3.273142534	1.710676428	0.02670498	1
Fam188b	66.02052471	152.8498895	37.07740311	0.242573961	-2.043503403	0.026778779	1
Cckar	6.993132201	27.9725288	0	0	#NAME?	0.027008581	1
Yod1	256.1502226	431.5761587	197.6749106	0.458030191	-1.126485397	0.027013217	1

Tkt	315.2427625	153.8489084	369.0407139	2.398721692	1.262265781	0.027014901	1
Rab25	426.8763696	229.7743437	492.5770449	2.143742582	1.100131679	0.027268315	1
Large	34.35443361	95.90581304	13.83730714	0.144280171	-2.793055054	0.027492468	1
Akr1c13	636.4737264	357.6487611	729.4153814	2.039474089	1.028197179	0.02752572	1
Gm15708	21.76937559	67.93328423	6.381406042	0.093936369	-3.412172364	0.027893013	1
Rasd2	295.4985845	139.862644	347.3772313	2.483702734	1.312492512	0.027978198	1
Rtl1	412.9101585	836.1788074	271.8206089	0.325074741	-1.621156637	0.028036621	1
Sh3bgrl	928.3369581	1321.701986	797.2152822	0.60317325	-0.729355647	0.028074381	1
Rpl14	719.7073424	418.5889131	820.0801521	1.95915402	0.970230821	0.028192572	1
H2-T23	395.3397492	80.92052975	500.1461557	6.180707878	2.62777208	0.028241097	1
Tmem132b	644.3293757	1097.921755	493.1319157	0.449150327	-1.15472971	0.028242648	1
Tsc22d3	148.4601677	50.94996318	180.9635692	3.55178999	1.828546281	0.02824553	1
Wapal	1783.682518	2434.609025	1566.707016	0.643514831	-0.635954696	0.028248159	1
Zbtb40	236.4597414	398.6085354	182.4101434	0.457617254	-1.127786646	0.028249357	1
Srsf9	220.4653512	93.90777526	262.6512099	2.796905892	1.483831712	0.02852641	1
Krt19	67.78111902	0	90.37482536	Inf	Inf	0.028557019	1
Thnsl2	28.78678948	81.91954864	11.07586976	0.135204233	-2.886787771	0.028643527	1
Dpp6	96.82691703	14.98528329	124.1074616	8.281956319	3.049971594	0.02882012	1
2410002I01Rik	60.90539575	142.8597007	33.58729411	0.235106849	-2.088611526	0.028865017	1
Stk38l	392.1372441	206.7969094	453.9173557	2.194991004	1.134215027	0.028876988	1
Tmem63a	825.3331167	1188.832474	704.1666643	0.592317824	-0.755556594	0.02889527	1
A930012L18Rik	44.49184722	111.8901152	22.02575789	0.196851687	-2.344819022	0.028922733	1
Slc2a5	1521.912328	1024.993377	1687.551978	1.646402812	0.719317352	0.029177951	1
1190007F08Rik	134.9711639	21.97841549	172.6354133	7.85477067	2.973569155	0.02932525	1
Caml	174.9386334	65.93524646	211.2730958	3.204251248	1.679987275	0.029372569	1
Tnfrsf11a	354.1594337	565.4446894	283.7310152	0.501783853	-0.994862047	0.029662032	1
BC018242	236.6350391	400.6065732	181.9778611	0.454255804	-1.138423146	0.029738824	1
Arhgap12	707.9075024	1036.981603	598.2161354	0.57688211	-0.793651571	0.02995507	1
Dclk2	52.16636091	126.8753985	27.26334839	0.214882859	-2.218377689	0.030022336	1
Commd6	423.2479066	228.7753248	488.0721005	2.133412337	1.093162831	0.030087306	1
Itgal	13.8046027	48.9519254	2.088828469	0.042671018	-4.550599675	0.030090052	1
Rhou	405.9221835	213.7900416	469.9662308	2.198260627	1.136362443	0.030096161	1
Anln	38.92398305	1.998037772	51.23263147	25.64147295	4.680407233	0.030273602	1
Ptch2	17.23784472	56.94407649	4.002434126	0.070287102	-3.830596212	0.030378232	1
Glt25d1	243.3967555	411.5957809	187.3304137	0.455132007	-1.135643049	0.030443736	1
Nr1d1	616.3618841	1195.825606	423.20731	0.35390387	-1.498570557	0.030488026	1
Ermp1	418.6148837	652.3593324	340.7000675	0.522258287	-0.937164615	0.030535576	1
Hdac10	81.66601894	174.828305	50.61192359	0.28949502	-1.788389566	0.030666006	1
Alg2	1949.894421	2632.414764	1722.38764	0.654299491	-0.611976946	0.030709216	1
Sh3pxd2a	1779.440319	2424.618836	1564.380814	0.645206904	-0.632166218	0.030796904	1
Has3	34.04248491	92.90875638	14.42039442	0.155210283	-2.687703948	0.030826702	1
Nrcam	961.1015519	610.4005392	1078.001889	1.766056581	0.820531565	0.030885609	1
Trappc3	593.7421697	285.7194013	696.4164258	2.437413849	1.285351227	0.03090873	1

Mipep	146.5737872	50.94996318	178.4483953	3.502424421	1.808353919	0.030956174	1
Lancl3	15.96268705	52.94800095	3.634249081	0.068638079	-3.86484701	0.031151145	1
Evi2a	19.38828216	62.93818981	4.871646285	0.077403661	-3.691454393	0.031161183	1
Nalcn	447.88373	694.3181256	365.7389314	0.526759878	-0.924782631	0.031538617	1
Bclaf1	3199.986761	4210.864604	2863.027481	0.6799144	-0.55657497	0.031545426	1
Mlxipl	3852.496031	6052.05641	3119.309237	0.515413114	-0.956198851	0.031551185	1
Tmem192	172.1285733	65.93524646	207.5263489	3.147426604	1.654172736	0.031670356	1
Brd1	549.1344206	317.6880057	626.2832255	1.971378253	0.979204616	0.031826872	1
Mrpl51	221.125411	96.90483192	262.5322707	2.709176266	1.437854262	0.031860927	1
Arpc5l	743.0002587	457.5506497	838.1501283	1.831819338	0.873277226	0.031881238	1
B2m	5682.826718	2994.059601	6579.082424	2.197378577	1.135783447	0.031911501	1
Pard6b	117.4158451	34.965661	144.8992398	4.144044062	2.051039343	0.032123969	1
Sc4mol	682.8242991	415.5918565	771.90178	1.857355403	0.8932499	0.03216798	1
Gnai3	1826.282195	1251.770664	2017.786039	1.61194546	0.688802932	0.032249902	1
E2f1	46.15162445	3.996075543	60.20347408	15.06564964	3.913190979	0.032263735	1
Gga3	339.2905317	534.4751039	274.2290076	0.513080975	-0.962741562	0.032270123	1
Hspa1a	33.81245367	0.999018886	44.75026526	44.7942135	5.485240472	0.032284916	1
Exoc4	765.3837048	1098.920774	654.2046815	0.595315601	-0.748273392	0.032318314	1
4930402H24Rik	616.7516914	910.106205	518.9668535	0.570226695	-0.810392514	0.03239363	1
Cnih	392.3348983	210.7929849	452.8488695	2.148310911	1.1032028	0.032409997	1
Cerkl	230.5959702	388.6183466	177.9218447	0.45783182	-1.127110361	0.032466749	1
Atp6v1g2	50.5544457	120.8812852	27.11216587	0.224287538	-2.156578631	0.032501943	1
Ppt2	30.03311394	83.91758641	12.07162312	0.143850933	-2.797353514	0.032503681	1
D730039F16Rik	62.77939741	4.995094429	82.04083173	16.42428044	4.037758261	0.032520251	1
Ung	101.1233926	26.97350992	125.8400202	4.665318699	2.221975639	0.032719314	1
Rapgef6	913.0266584	1289.733382	787.4577507	0.610558556	-0.711798431	0.03280829	1
Lpl	1984.053266	2691.356878	1748.285396	0.649592557	-0.622392993	0.032845209	1
Alkbh5	1211.417307	802.2121653	1347.81902	1.680127875	0.748571041	0.032927002	1
Tmem188	525.5916088	300.7046846	600.5539168	1.997155174	0.997946431	0.032967932	1
Zfp182	361.5518692	568.441746	292.5885769	0.514720425	-0.958139063	0.033167456	1
Prom1	1821.978073	3339.720135	1316.064053	0.394064173	-1.343497505	0.0331934	1
Cacna1b	24.90148461	72.92837866	8.89251993	0.121934974	-3.035816113	0.033319288	1
Cobra1	378.0698209	598.4123126	304.6223237	0.509050896	-0.974118189	0.033416803	1
Cadps	2814.514829	3727.339463	2510.239952	0.673466953	-0.57032094	0.033430775	1
Ccdc19	21.95060847	67.93328423	6.623049887	0.097493445	-3.358550973	0.033438865	1
Inha	285.7623448	497.5114051	215.1793246	0.43251134	-1.209190135	0.033452024	1
Oxa1l	303.1103817	150.8518518	353.863225	2.345766531	1.230059432	0.033485152	1
Lrrc27	210.8203696	392.6144221	150.2223521	0.382620565	-1.386013677	0.033511554	1
Agrn	135.7166843	252.7517781	96.70498641	0.382608531	-1.386059055	0.033542895	1
Calca	11.13515578	40.95977432	1.193616268	0.029141183	-5.100796758	0.033579798	1
D430042O09Rik	289.4284907	474.5339708	227.726664	0.479895388	-1.059208147	0.033708755	1
Cdyl	99.48693763	198.8047583	66.38099742	0.333900446	-1.582510075	0.033755761	1
Ripk2	352.9073756	134.8675496	425.5873175	3.155594647	1.657911895	0.033757085	1

Psmb10	199.4034123	52.94800095	248.2218827	4.688031243	2.228982185	0.033790506	1
1700016K19Rik	53.7693116	125.8763796	29.73362227	0.236212881	-2.081840457	0.033876797	1
Fgl2	618.6196473	369.6369877	701.6138671	1.898115964	0.924568135	0.033909888	1
Ocrl	1252.952063	829.1856752	1394.207526	1.681417767	0.749678222	0.033930018	1
Bid	42.01933958	105.8960019	20.7271188	0.195730891	-2.353056629	0.034110874	1
Gatsl2	1141.572364	1590.438066	991.9504629	0.623696379	-0.681084212	0.034194451	1
C130021I20Rik	20.80197877	64.93622758	6.090562498	0.093792983	-3.414376194	0.034254065	1
Acsf2	6.493622758	25.97449103	0	0	#NAME?	0.034254505	1
4930480K15Rik	81.30357862	170.8322295	51.46069501	0.301235283	-1.731037335	0.034323662	1
Dync2h1	1295.078004	1768.263428	1137.34953	0.64320141	-0.636657526	0.034375352	1
Gp5	12.67826188	44.95584986	1.919065891	0.04268779	-4.550032706	0.034458043	1
Pafah2	87.70866508	180.8224183	56.67074733	0.313405538	-1.673897421	0.034608395	1
Mrpl40	128.7491532	41.9587932	157.6792733	3.757955394	1.909947943	0.034674305	1
Hes1	65.1935205	10.98920774	83.26162476	7.576672195	2.921564331	0.034770781	1
A2ld1	181.8912083	73.92739755	217.8791452	2.947204317	1.55934708	0.035052855	1
Bcl11a	85.9760757	177.8253617	55.35964705	0.311314688	-1.683554448	0.035114355	1
Itpr1	328.7101682	592.4181993	240.8074912	0.406482265	-1.298735688	0.035143064	1
Star	754.0175236	467.5408386	849.509752	1.816974437	0.861538123	0.035164396	1
Pcbd1	1649.467277	1029.988471	1855.960212	1.801923288	0.849537593	0.035275485	1
1500016L03Rik	81.02638404	166.8361539	52.42312741	0.314219228	-1.670156631	0.035326321	1
Vapa	633.6795854	384.622271	716.6986902	1.863383231	0.897924415	0.035348524	1
Gm3414	209.6468415	357.6487611	160.3128683	0.448241084	-1.157653209	0.035404818	1
Crip1	134.8524397	45.95486875	164.4849633	3.579271747	1.839666081	0.035514852	1
Tmem9	424.0414256	655.3563891	346.9364378	0.529385909	-0.9176083	0.03561348	1
4930420K17Rik	240.8600804	398.6085354	188.277262	0.472336253	-1.082113825	0.035785087	1
Gtdc1	232.9892508	387.6193277	181.4458918	0.468103314	-1.095101116	0.035793743	1
Dph1	32.9602313	0.999018886	43.61396877	43.65680108	5.448134517	0.036122306	1
Rit2	86.8842696	180.8224183	55.57155336	0.307326679	-1.702155081	0.036205907	1
Cpox	594.6332651	875.140544	501.1308388	0.572628982	-0.804327405	0.036347645	1
Fbxl21	57.03708368	131.8704929	32.09261393	0.243364632	-2.03880858	0.036536019	1
Gm15910	18.18958051	58.94211426	4.60540259	0.07813433	-3.677899628	0.036651517	1
Fgfr1	191.6973399	333.6723079	144.3723506	0.432677052	-1.208637487	0.03669769	1
Rpl29	175.4051724	413.5938187	96.00895697	0.232133443	-2.106973714	0.036809129	1
Fyco1	1037.00701	1430.595044	905.8109987	0.633170793	-0.659333387	0.037022356	1
Bccip	904.2765193	582.4280104	1011.559356	1.736797231	0.796429331	0.037227595	1
Eya3	640.1676353	929.0875638	543.8609925	0.585371082	-0.772576618	0.037316446	1
Itch	1067.259551	1478.547951	930.1634177	0.629106021	-0.668624926	0.037331564	1
Dlgap3	16.43771463	53.94701983	3.934612898	0.072934759	-3.777249654	0.037453099	1
Npat	721.5221567	1033.984547	617.3680266	0.597076647	-0.744011951	0.037490012	1
Gadd45g	386.5939077	209.793966	445.5272216	2.123641733	1.086540398	0.037501014	1
Col6a6	1037.413981	1431.594063	906.0206201	0.632875368	-0.660006676	0.0376085	1
Mpp7	281.7987681	453.5545742	224.5468328	0.49508228	-1.014259781	0.037672452	1
Myeov2	172.9072993	58.94211426	210.8956943	3.578013733	1.839158925	0.037889192	1

Ssr4	1518.537204	1037.980622	1678.722732	1.617296793	0.693584455	0.037941938	1
Csnk1g3	475.8952089	718.2945789	395.0954189	0.5500465	-0.862374509	0.037982856	1
Ndufa3	197.8399194	84.91660529	235.4810242	2.773085704	1.471492204	0.038012431	1
Slco1a6	290.5032127	466.5418197	231.8236771	0.496897957	-1.008978484	0.03801675	1
Fam125a	165.735943	61.93917092	200.3348671	3.23438083	1.693489558	0.038030874	1
Pgap2	82.54651485	173.8292861	52.11892443	0.299828214	-1.737791946	0.038100274	1
Fut1	116.3612426	225.7782682	79.8889007	0.353837866	-1.498839647	0.038100393	1
Lars2	34968.2868	11333.86926	42846.42598	3.780388233	1.918534402	0.03841367	1
1700008F21Rik	6.243868036	24.97547214	0	0	#NAME?	0.038564458	1
1700111N16Rik	6.243868036	24.97547214	0	0	#NAME?	0.038564458	1
Rabl5	116.5071338	35.96467989	143.3546184	3.985983438	1.994935716	0.038599525	1
Cbx6	370.1692715	198.8047583	427.2907759	2.149298536	1.103865886	0.038608303	1
Runx1t1	1464.918492	1991.044639	1289.543109	0.647671621	-0.626665564	0.038777165	1
Scml4	198.6281158	83.91758641	236.8649589	2.822590223	1.497019697	0.038814046	1
Lig3	557.3417161	814.2003919	471.7221575	0.57936862	-0.787446548	0.038824344	1
Slc41a2	83.6882676	171.8312484	54.30727401	0.316050046	-1.661775068	0.038977361	1
Xrcc4	144.6674702	51.94898206	175.573633	3.379731922	1.756908817	0.038984727	1
Pak1	231.7750814	82.91856752	281.3939194	3.393617712	1.762824056	0.039401327	1
Pcyt1b	382.9908946	590.4201615	313.8478056	0.531566884	-0.911676865	0.039416055	1
Pdk1	225.2780886	380.6261955	173.4953863	0.455815675	-1.133477556	0.03951726	1
Gpr25	14.44134476	47.95290652	3.270824173	0.068209091	-3.87389215	0.039679673	1
Gm13003	19.72230329	60.94015203	5.983020375	0.098178626	-3.348447218	0.039741345	1
Ssh3	148.1395135	267.7370614	108.2736641	0.404402975	-1.306134484	0.039762189	1
Sestd1	325.6545684	512.4966884	263.3738617	0.513903539	-0.960430508	0.039828053	1
Aga	492.929818	759.2543532	404.154973	0.532305111	-0.909674675	0.039843085	1
1200011M11Rik	150.047541	56.94407649	181.0820292	3.179997646	1.669025697	0.039997709	1
Kcng3	75.9120461	162.8400784	46.93603533	0.288233927	-1.794687933	0.040154196	1
Swi5	603.5524478	330.6752512	694.5115133	2.100282712	1.070583537	0.040359929	1
Alkbh7	94.82702799	25.97449103	117.7778736	4.534366949	2.180901148	0.040401824	1
1700034H15Rik	26.89656089	75.92543532	10.55360275	0.138999568	-2.846847691	0.040481069	1
Arhgap44	556.8291563	813.201373	471.3717507	0.579649477	-0.78674735	0.040828083	1
Sobp	291.885678	468.5398574	233.0009515	0.497291634	-1.007835933	0.040831174	1
Klhl20	276.1761088	453.5545742	217.0499537	0.478553114	-1.063249039	0.041054093	1
BC018507	904.719862	1260.761834	786.0392047	0.623463674	-0.681622591	0.041181934	1
Gtf2e2	251.973752	412.5947998	198.4334027	0.480940145	-1.056070741	0.041205402	1
Fnbp1	166.4321352	296.7086091	123.006644	0.414570525	-1.270310546	0.041309189	1
B430010I23Rik	38.87539014	96.90483192	19.53224288	0.201561083	-2.310710981	0.041504332	1
Ttll4	174.5304066	303.7017413	131.4732951	0.432902671	-1.207885392	0.041724952	1
Rasgrf2	698.9043068	1006.012018	596.5350697	0.592970123	-0.75396868	0.04174535	1
Kif3c	153.6858138	316.6889868	99.35142281	0.313719223	-1.672454163	0.041970796	1
Fam135b	493.3356969	837.1778263	378.7216538	0.452378983	-1.144396189	0.041999055	1
1700096K18Rik	55.56817067	7.992151086	71.42684387	8.937123823	3.159810613	0.042049654	1
Mycbp	378.7954382	187.8155505	442.4554007	2.355797481	1.236215521	0.042160556	1

Pcdha5	15.29633054	50.94996318	3.411786322	0.06696347	-3.900481904	0.042320964	1
Zfp239	49.86660699	116.8852096	27.52707277	0.235505184	-2.086169279	0.042388394	1
Vps36	129.2122279	44.95584986	157.2976873	3.498937019	1.806916696	0.042448637	1
Mpzl3	31.62413353	0.999018886	41.83250508	41.87358786	5.387968633	0.042498392	1
Bpgm	325.2342884	172.8302672	376.0356287	2.175751011	1.121513467	0.042542248	1
Gm13315	19.35404746	59.94113315	5.8250189	0.097178992	-3.363211721	0.042748193	1
Cib1	98.59785558	27.9725288	122.1396312	4.366413635	2.126448805	0.042816885	1
Lss	444.3737144	665.3465779	370.7160933	0.557177425	-0.843791288	0.042846109	1
Cgn	341.2534966	161.8410595	401.0576423	2.478095753	1.309231934	0.043029662	1
6720401G13Rik	708.2921565	1002.015942	610.3842278	0.609156204	-0.715115874	0.043153154	1
Cck	23.46199983	68.93230312	8.305232069	0.120483891	-3.053087832	0.043176819	1
4831426I19Rik	5.994113315	23.97645326	0	0	#NAME?	0.043406197	1
Naip5	5.994113315	23.97645326	0	0	#NAME?	0.043406197	1
Slc19a1	117.2730291	35.96467989	144.3758122	4.014377792	2.005176395	0.04342534	1
Tlcd2	19.80295988	60.94015203	6.090562498	0.099943343	-3.322745719	0.043468713	1
Tmem146	69.69604457	146.8557762	43.97613402	0.29945117	-1.739607325	0.043651597	1
Zfp599	38.48616447	95.90581304	19.34628161	0.201721679	-2.309561958	0.04368355	1
Cyp51	703.8108844	444.5634042	790.2267111	1.777534326	0.82987742	0.043703261	1
Atp1b3	802.7466679	510.4986506	900.1626737	1.763300789	0.818278595	0.043853654	1
Tanc1	280.0979358	448.5594797	223.9440878	0.499251711	-1.002160723	0.043854075	1
Mpnd	164.6233829	65.93524646	197.5194284	2.995657694	1.58287278	0.043856981	1
Bod1l	1785.863717	2383.659062	1586.598602	0.665614738	-0.587240717	0.043869134	1
Nt5c3	1750.578747	1228.79323	1924.507252	1.566176641	0.647246936	0.043883072	1
Tmem68	341.1179256	183.819475	393.5507424	2.14096326	1.098260039	0.043961585	1
Snap23	1074.988393	1489.537159	936.8054707	0.628923868	-0.669042708	0.043977628	1
Myo1b	356.8714936	192.810645	411.5584432	2.134521376	1.09391261	0.044240825	1
Ralgds	611.1018047	374.6320822	689.9250455	1.841606948	0.880965182	0.044280008	1
Nfkb2	103.9989484	26.97350992	129.6740945	4.807460911	2.265275127	0.044428948	1
Rai1	590.7922607	850.1650718	504.3346571	0.593219686	-0.75336162	0.044470426	1
Scaf1	218.0458849	364.6418933	169.1805488	0.463963554	-1.107916614	0.044621603	1
Ankrd11	2750.923663	3601.463083	2467.410523	0.685113374	-0.545585348	0.044947955	1
Abhd6	352.2892857	188.8145694	406.7808578	2.154393377	1.1072817	0.044967168	1
Mrpl54	138.046439	49.95094429	167.4116039	3.351520302	1.744815673	0.045236104	1
Atxn2l	3583.795672	4697.386801	3212.598629	0.683911878	-0.548117649	0.045346061	1
Ctsz	215.7447522	99.90188858	254.3590401	2.546088404	1.348282513	0.045424565	1
Polr3a	446.700437	663.3485402	374.4844026	0.564536409	-0.824861467	0.045556353	1
Rps18	325.3831979	174.828305	375.5681622	2.148211425	1.103135989	0.04588636	1
Mob3b	56.62923702	127.8744174	32.88084356	0.257133868	-1.95940845	0.045965808	1
She	12.47491633	42.95781209	2.313951075	0.053865664	-4.21449025	0.046093604	1
Irak1bp1	220.6384159	365.6409122	172.3042505	0.47123898	-1.085469214	0.046171027	1
Pkd1	1134.637486	1544.483197	998.0222489	0.646185242	-0.629980293	0.046217842	1
Ano7	27.81179819	75.92543532	11.77391915	0.155072132	-2.688988649	0.046283956	1
Csf1	212.7565966	358.64778	164.1262021	0.457625033	-1.127762123	0.046407393	1

Ccdc39	13.84325391	45.95486875	3.139382297	0.068314466	-3.871665089	0.046425822	1
Kcnq1ot1	3601.321759	5102.988469	3100.76619	0.607637311	-0.718717637	0.046496321	1
Smad1	568.2360619	345.6605345	642.4279044	1.858551499	0.894178664	0.046607471	1
Pitpnm2	689.733323	972.0453759	595.6293054	0.612758746	-0.706608924	0.046757373	1
Taf13	199.7815131	72.92837866	242.0658913	3.319227655	1.730847583	0.0469122	1
Fam167a	118.0057203	218.785136	84.4125818	0.385824117	-1.373984771	0.046942003	1
Nme2	652.8173891	333.6723079	759.1990829	2.275283459	1.18604629	0.047036827	1
Jam3	75.74918314	158.8440028	48.05090991	0.302503771	-1.724974966	0.047098099	1
Srsf11	1719.835871	2270.769927	1536.191185	0.676506751	-0.563823764	0.047169267	1
Ogg1	39.73988531	98.90286969	20.01889052	0.202409602	-2.304650362	0.047256778	1
Cd164l2	38.20558731	96.90483192	18.63917244	0.192345129	-2.378230796	0.047258518	1
Cryba2	53.81001578	121.8803041	31.11991969	0.255331819	-1.969554762	0.047285347	1
D3Ertd254e	510.427111	748.2651455	431.1477662	0.576196511	-0.79536717	0.047299613	1
Cirbp	514.7914101	303.7017413	585.154633	1.926741119	0.946162741	0.047542364	1
Prkch	11.60688216	40.95977432	1.822584774	0.044496944	-4.490149941	0.047663063	1
Rpap1	322.700142	499.5094429	263.7637083	0.52804549	-0.921265876	0.047685407	1
Btbd3	345.2249917	188.8145694	397.3617991	2.104508144	1.073483093	0.047694919	1
Asb8	485.2959285	286.7184202	551.4884313	1.923449602	0.943696029	0.047756184	1
Chaf1a	27.79717458	76.92445421	11.42141471	0.148475733	-2.751700942	0.04781994	1
Ubqln4	360.5163351	551.458425	296.8689718	0.538334276	-0.89342581	0.047822747	1
Mga	3051.20602	3969.102033	2745.240682	0.691652837	-0.53188001	0.047893624	1
Rgs7	671.0251807	418.5889131	755.1706032	1.80408649	0.851268505	0.047915687	1
Sema3d	7.24105553	27.9725288	0.330564439	0.011817467	-6.40293534	0.047935971	1
A930005I04Rik	24.25730846	0	32.34307795	Inf	Inf	0.048090068	1
Nfkbib	211.3600686	96.90483192	249.5118142	2.57481293	1.364467619	0.048264453	1
Lrch2	30.65251435	81.91954864	13.56350292	0.165571016	-2.594477949	0.048266533	1
Tshz2	1019.413196	1397.627421	893.3417874	0.639184502	-0.645695666	0.048432881	1
3110040N11Rik	151.2004415	58.94211426	181.9532172	3.086981515	1.626196844	0.048434727	1
Nav1	2383.79572	3134.921264	2133.420538	0.68053401	-0.555260832	0.04844276	1
2610044O15Rik	83.85312195	172.8302672	54.19407352	0.313568187	-1.673148897	0.048462428	1
4932415G12Rik	328.2725082	510.4986506	267.5304608	0.524057136	-0.932203983	0.0485336	1
Dapl1	278.3141171	137.8646062	325.1306208	2.358332785	1.237767312	0.048738643	1
2410007B07Rik	5.744358593	22.97743437	0	0	#NAME?	0.048842764	1
Rgs2	346.7690603	157.844984	409.7437524	2.595861725	1.376213537	0.049010372	1
Prpf39	619.0659424	881.1346573	531.7097041	0.603437511	-0.728723715	0.049190275	1
Hsf4	44.59043157	3.996075543	58.12188359	14.54474095	3.862425696	0.049457707	1
Adcy7	22.1276535	65.93524646	7.525122515	0.114128981	-3.131262906	0.049465555	1
Lsm3	83.66690046	21.97841549	104.2297288	4.742367749	2.245607542	0.049537204	1
Srp14	702.3585545	260.7439292	849.5634296	3.258228992	1.704088002	0.049796704	1
Ascc1	121.8551864	225.7782682	87.21415909	0.386282346	-1.372272351	0.049940491	1
Serac1	270.499227	425.5820453	218.8049543	0.514131075	-0.959791882	0.049965211	1

This table lists all genes differentially expressed between wild-type and triple Foxo-deficient ALDH^{+} cells, arranged by p-value

Supplementary Table 5 Overview of key differential changes in transcript profile of wild-type and Foxo knockout ALDH$^+$ cells

Change	*Gene or network*	*Wild-type*	*Foxo KO*
↑↑↑	ALDH1A3		
↑	Differentiation Factors	Bach2<br>Pax6<br>Rfx6<br>Rfx7<br>Hic2<br>NcoR	↓
	LncRNA	Malat1<br>Meg3<br>Peg3<br>Neat1<br>KcQ1ot1<br>Sngh11	↓
↓	Gpcrs	Gipr<br>Gpr116<br>Gpr137<br>Gpr98	
	Cytochrome	Cyp27b1<br>Ndor	↓↓↓<br>↓↓<br>Cyb5r3<br>Elovl6
	Ribosomes	40S Subunit<br>60S Subunit	
	Mitochondria	Complex I<br>Complex IV<br>Complex V	Complex III
	Differentiation markers	Insulin<br>IAPP<br>Cpe<br>ChgB<br>Gcg<br>Pyy<br>Npy	

Category list of principal genes altered in ALDH$^+$ cells as a function of Foxo genotype. Upward arrows indicate genes with increased expression, downward arrows indicate genes with decreased expression. Arrows in the Foxo column indicate that the change is specific to Foxo knockout ALDH$^+$ cells.
